# A critical comparison of topology-based pathway analysis methods

**DOI:** 10.1371/journal.pone.0191154

**Published:** 2018-01-25

**Authors:** Ivana Ihnatova, Vlad Popovici, Eva Budinska

**Affiliations:** 1 RECETOX, Faculty of Science, Masarykova Univerzita, Brno, Czech Republic; 2 Institute of Biostatistics and Analyses, Faculty of Medicine, Masarykova Univerzita, Brno, Czech Republic; College of Bioinformatics Science and Technology, CHINA

## Abstract

One of the aims of high-throughput gene/protein profiling experiments is the identification of biological processes altered between two or more conditions. Pathway analysis is an umbrella term for a multitude of computational approaches used for this purpose. While in the beginning pathway analysis relied on enrichment-based approaches, a newer generation of methods is now available, exploiting pathway topologies in addition to gene/protein expression levels. However, little effort has been invested in their critical assessment with respect to their performance in different experimental setups. Here, we assessed the performance of seven representative methods identifying differentially expressed pathways between two groups of interest based on gene expression data with prior knowledge of pathway topologies: SPIA, PRS, CePa, TAPPA, TopologyGSA, Clipper and DEGraph. We performed a number of controlled experiments that investigated their sensitivity to sample and pathway size, threshold-based filtering of differentially expressed genes, ability to detect target pathways, ability to exploit the topological information and the sensitivity to different pre-processing strategies. We also verified type I error rates and described the influence of overexpression of single genes, gene sets and topological motifs of various sizes on the detection of a pathway as differentially expressed. The results of our experiments demonstrate a wide variability of the tested methods. We provide a set of recommendations for an informed selection of the proper method for a given data analysis task.

## Introduction

High-throughput gene expression technologies (microarrays or next-generation sequencing) allow the estimation of the expression levels of thousands of genes in a single experiment. Often these experiments are just a first step in a broader biological investigation and serve generating hypotheses based on identified differentially expressed genes and pathways. A biological pathway is a collection of genes or molecules that act synergistically by means of chemical reactions, molecule modifications or signal transduction to execute a biological function. Thus, from a computational analysis perspective, a pathway is a set of genes (proteins) and their associated pairwise interactions. Pathway analysis aims to discover those pathways whose activation/inactivation is associated with a group of interest. This type of analysis requires integrating information about gene ontology and pathway structure.

Generally, there are two main approaches: one that relies only on the expression levels of the constituent genes (of the pathway)—and is epitomised by the GSEA family of methods—and a second one that additionally exploits the pathway topology. The second group of methods represents a more recent evolution of pathway analysis methods that try to improve both specificity and sensitivity of the findings.

The application of topology-based methods is facilitated by the existence of public databases which gather information about gene/protein interactions, such as the well-known Kyoto Encyclopedia of Genes and Genomes (KEGG) database which provides access to hundreds of pathways representing state-of-the-art knowledge about molecular interactions. Prior to performing a topology-based pathway analysis, the pathway of interest must be pre-processed into a simple interaction network.

Each new topology-based pathway method usually compares its performance to an enrichment-based method (most often GSEA [1]) on a set of benchmark datasets. Sometimes, the underlying mathematical model is verified by simulations. The reviews that include topology-based pathway analysis methods either examine their algorithms from mathematical perspective [2–4] or their performance on both real and simulated data [5, 6]. The latter approach revealed that topology-based methods outperform enrichment-based methods in accuracy and sensitivity only for non-overlapping pathways [5] and that the FCS variant of CePa [7] method exhibits the best cross-study concordance [6]. However, there are multiple limitations to the existing comparisons which hamper the identification of actionable information about the most appropriate method for a given analytical problem. First, the comparison of a topology-based method with enrichment-based methods is oversimplistic as it does not investigate the topological aspects of pathway deregulation (position and biological importance of a gene in a pathway, deregulation of topological motifs etc.). Second, the existing reviews do not examine the effect of pathway topology pre-processing strategy or whether the inclusion of the pathway topology information in the analysis has actually any effect at all. Third, multiple other effects, such as sample size (crucial aspect in biological experiments) or the effect of a deregulation of a single or very few genes, are not explored either.

Given the proliferation of methods (see [8] for a review of 22 methods) and with limited insight into their performance, data analysts are confronted with the difficult task of selecting the best-suited method for analysing the data at hand. We propose a systematic investigation of several prominent recently proposed methods and provide a simple guideline for decision-making.

In this work, we consider a number of parameters that influence the quality of the results obtained by topology-based pathway analysis. These parameters are varied in controlled experiments in order to study the sensitivity of the methods and—when possible—to quantify it. These experiments are performed on both artificial and real-world data, thus resulting in a comprehensive characterisation of the behaviour of each considered method. From the beginning, we did not expect to identify a single method that would fit all possible applications, thus, in our investigations, we tried to capture most of the standard scenarios. The methods under investigation were selected based on the following criteria: (i) the aim is to detect differentially expressed pathways (DEPs) between two groups of interest based on gene expression data; (ii) the pathway topology is a priori known and is modeled as simple interaction network or graph *G* = (*V*, *E*), where *V* is a set of vertices/nodes represented by products of genes and *E* is a set of edges representing interactions between them; (iii) the pathways are modeled and analyzed individually (without cross-pathway interactions). The typical input data for these methods consists of a gene expression data matrix (log2-transformed normalised expression profiles from a high-throughput technology after standard pre-processing), group membership labels (as a vector) and the list of pathway topologies. Based on these criteria we selected the following methods: SPIA [9], PRS [10], CePa [7], TAPPA [11], TopologyGSA [12], Clipper [13] and DEGraph [14]. Each method assigns a test-statistic and a *p*-value to each pathway (possibly other parameters like the number of differentially expressed genes, pathway size etc.) and pathways with extreme test-statistic or low *p*-value are called‘differentially expressed’.

## Materials and methods

**Fig 1:**
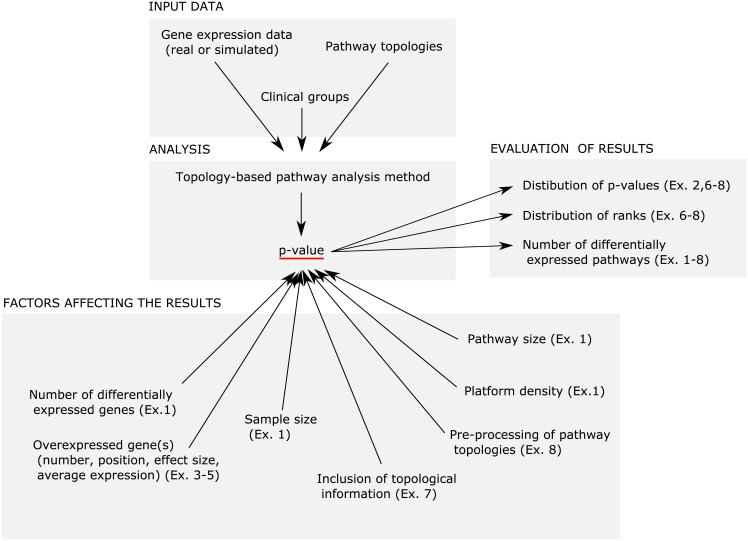
Overview of the eight controlled experiments (Ex. 1-8) performed.

We performed eight distinct experiments to provide comprehensive insight into the topology-based pathway analysis methods (Fig 1, Table 1, S1 Text). In these experiments, we examined the influence of the number of parameters on the results obtained by topology-based pathway analysis methods. A detailed description of the experiments can be found in the S1 Text.

**Table 1:** Overview of the experiments performed to evaluate methods’ performance.

Experiment	Parameter(s) under study	Varied parameter(s)*	Datasets	Pathway topologies	Evaluation criterion †
1	Effect of sample size, pathway size and significance thresholds for DEGs	*n*_1_, *n*_2_, |*V*|, *θ*	Simulated, Real	graphite	Prop. DEPs
2	Type I error rate	*y*	Simulated	graphite	Prop. DEPs, histogram
3	Single gene overexpression	*X*_*ij*_, *i* ∈ *I* ⊂ *V*, |*I*| = 1, *j* ∈ 1, 2, 3, …, *n* such that *y*_*j*_ = 1	Simulated	graphite	Prop. DEPs
4	Multiple genes overexpression	*X*_*ij*_, *i* ∈ *I* ⊂ *V*, |*I*| ∈ 2, 3, 4, 5, *j* ∈ 1, 2, 3, …, *n* such that *y*_*j*_ = 1	Simulated	graphite	Prop. DEPs
5	Topological motif overexpression	*X*_*ij*_, *i* ∈ *I* ⊂ *V*, |*I*| ∈ 3, 4, 5, *j* ∈ 1, 2, 3, …, *n* such that *y*_*j*_ = 1	Simulated	graphite	Prop. DEPs
6	Target pathway detection	*X*_*ij*_, *i* ∈ *I* ⊂ *V*, |*I*| = 1, *j* ∈ 1, 2, 3, …, *n* such that *y*_*j*_ = 1	Simulated, Real	graphite	Median *p*-value, rank
7	Inclusion of topological information	PT	Simulated, Real	graphite ‡	Prop. DEPs
8	Pre-processing of pathway topologies	PT	Simulated, Real	ToPASeq	Prop. DEPs

*X is a normalized log_2_-transformed gene expression data matrix of expression profiles of *p* genes (rows) and *n*_1_ + *n*_2_ samples (columns), *n*_1_ and *n*_2_ denote number of samples in two compared groups, *y* is a vector of 1’s and 2’s assigning samples into the groups, *PT* is a set of pathway topologies (graphs) *G* = (*V*, *E*), where *V* is a set of vertices/nodes represented by products of genes and *E* is a set of edges representing interactions between them, *θ* is the threshold used for detection of DEGs;

^†^Prop. DEPs denotes Proportion of Differentially Expressed Pathways;

^‡^without interactions

The first group of parameters are data set-centric (sample size, pathway size, number of DEGs in the dataset and thresholds used to detect DEGs; Experiment 1) and helped us to describe the performance of a method under various conditions and to guide the selection of the optimal method for a specific dataset. The methods’ ability to control type I error was studied in Experiment 2. The influence of overexpression of particular gene(s) (Experiments 3-5), the influence of discarding the topological information (Experiment 7) and the effect of the pre-processing of pathway topologies (Experiment 8) tested the topology-based nature of the methods. If no effects were observed, the method should not be considered as topology-based pathway analysis method. The increased sensitivity and specificity expected from the incorporation of the topological information were examined by the identification of biologically relevant pathways (Experiments 6-8) since no proper method for identifying truly differentially expressed pathways is known.

Following the categorization of GSEA methods, the topology-based pathway analysis methods can be grouped based on three main criteria: (i) the null hypothesis (*competitive* and *self-contained*); (ii) the (non)identification of differentially expressed genes (DEGs) prior pathway analysis (*over-representation analysis (ORA)* and *functional class scoring (FCS)*) and (iii) the number of variables in the model (*univariable* and *multivariable*) (see S1 Text for the details). We will use these categories in methods evaluation.

For each experiment we applied selected methods (Table 2) on gene expression datasets, looking for differentially expressed pathway(s) between two groups of interest from a collection of pathways. In ORA methods we detected differentially expressed genes with moderated t-test [15] and significance level *α* = 0.05, unless stated otherwise. For all methods estimating significance threshold using permutations, the number of permutations was set to 1000. The pathways were considered differentially expressed if their *p*-value was below the significance threshold *α* = 0.05. All the analyses were performed in R statistical framework [16] and Bioconductor [17]. There are multiple freely-available implementations of the selected topology-based pathway analysis methods: (i) original implementation (all but TAPPA), (ii) graphite package (SPIA, TopologyGSA, Clipper, DEGraph) [18] and (iii) ToPASeq package [19] (all methods). ToPASeq package is our previous work in which we either de novo implemented or optimised existing implementations of a number of existing topology-based pathway analysis methods. For the sake of access uniformity for method application and access to method-specific pre-processing, we chose to use the ToPASeq package in our work.

**Table 2:** Overview of the selected methods.

	SPIA	PRS	CePa	TAPPA	TopologyGSA	Clipper	DEGraph
Reference	[9, 20, 21]	[10]	[7]	[11]	[12]	[13]	[14]
Null hypothesis	C	C	C	*	SC	SC	SC
ORA/FCS	ORA	ORA	ORA	FCS	FCS	FCS	FCS
Type	U	U	U	U	M	M	M
Pathway model	DG	DG	UG, DG	UG	DAG	DAG	UG
Node statistic	Log FC	Log FC	Log FC	-	-	-	-
Topology usage	Perturbation factor	Downstream DEG	Centrality	PCI	GGM, IPS	GGM, IPS	GL, FT
Pathway statistic	Impact factor	Sum	Sum	*	*T*^2^	*T*^2^	*T*^2^
Statistical significance	Gene perm.	Gene perm.	Gene perm.	*	Sample perm.	Sample perm.	F-distribution

SC = self-contained, C = competitive, ORA = over-representation analysis, FCS = functional class scoring, M = multivariable, U = univariable, DAG = directed acyclic graph, UG = undirected graph, DG = directed graph, PCI = Pathway Connectivity Index, GGM = Graphical Gaussian Models, IPS = Iterative Proportional Scaling, GL = Graph Laplacian, * = various statistics are possible, for detection of differentially expressed pathways between two conditions authors suggests Mann-Whitney test.

The following section describes gene expression data matrices and pathway topologies used in each experiment. We do not define the basic terms from graph theory, since they are explained in many textbooks, for example [22]. Statistical details of individual experiments and key properties of the compared methods are described in the S1 Text.

### Real datasets

In our study we used real gene expression microarray datasets from three public collections: Gene Overexpression Data Collection [23, 24], Breast Cancer Data Collection [25] and Disease Control Data Collection [26, 27]. These collections were obtained and pre-processed as described in the S1 Text. For each real dataset, we can anticipate one or several pathways that are expected to be differentially expressed or their identification is of particular interest due to experimental design. However, those pathways cannot be called ‘true positive’. The Gene Overexpression Data Collection was selected because it allows us to study the effect of one perturbed gene. The Breast Cancer Data Collection represents a collection of datasets related to the same biological problem, and we focus on the reproducibility of the results. In the Disease-Control Data Collection, datasets cover various biological problems (cancer, metabolic, neurodegenerative diseases etc.) in a unified experimental design in which expression profiles of patients are compared to healthy controls. Additionally, we can identify a single pathway (*target pathway*) which is directly related to the particular disease and hence very likely to be differentially expressed. These datasets were used in Experiments 1, 6, 7 and 8.

### Simulated datasets

Since the proper statistical distribution of the pathway expression data is unknown, we decided to use a real dataset (a dataset from Breast Cancer Data Collection denoted as VDX) as a base for the generation of simulated data. It contains 344 expression profiles of breast tumours obtained on an Affymetrix Human Genome U133A Array platform with 22 283 probesets corresponding to 13 091 unique Entrez IDs. We used estrogen receptor status as the main parameter dividing samples into two clinical groups. The simulated datasets were used in all experiments. The datasets for particular experiments were generated as shown in S1 Text.

### Pathways and their topologies

We used human pathways from the KEGG database as the source of pathway topologies. For our comparison we used graphite’s pre-processed pathways as a default set of pathway topologies for the following reasons: (i) they are claimed to be superior to original implementation [28]; (ii) they allowed us to compare only the methods’ algorithms regardless of the pre-processing strategy; (iii) the details of the pre-processing strategy are rarely described in the corresponding publication; (iv) the graphite implementation is readily available and widely used. In ToPASeq one can choose either graphite pre-processed pathways (+GPT) or pathway pre-processing as in the original implementation (MSPT) (if available) and hence evaluate the effect of different pre-processing strategies. The +GPT topologies were used in all experiments, and the MSPT was used in Experiment 8 only. In Experiment 7 we also used non-topological variants of the compared methods corresponding to pathway topologies without interactions (-GPT). To reduce computational complexity, we filtered out pathways with more than 150 genes and with less than two genes with available expression data.

## Results

### Experiment 1: Effect of sample size, pathway size, platform density and number of differentially expressed genes

**Fig 2:**
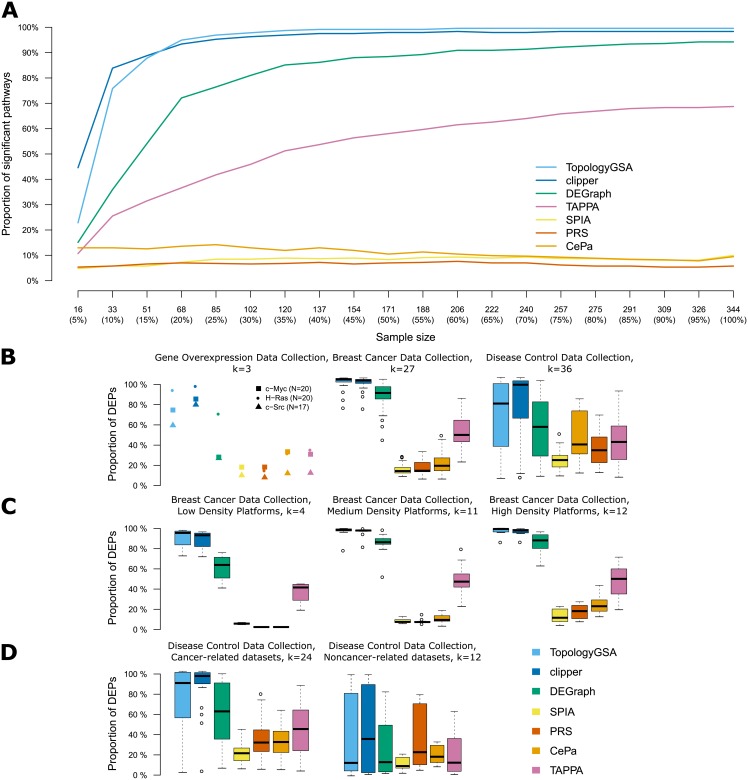
The effect of sample size. (A) The selected dataset from Breast Cancer Data Collection (denoted as VDX) was reduced to 20 random subsets representing 5%, 10%, … 95% of its original sample size (while preserving the proportion of samples in the clinical groups) leading to sample sizes from 16 to 326. Differentially expressed pathways between estrogen receptor positive and negative samples were detected. The lines show the median proportion of significant pathways (*p* < 0.05) over 20 subsets for each sample size. (B-D) Graphs indicating the percentage of differentially expressed pathways (DEPs) in the respective data collections. *k* denotes the number of datasets. See Table 3 for the summary of sample sizes. The datasets from the Breast Cancer Data Collection were divided by platfrom densities into: low-density platforms (2780-5486 EntrezIDs), medium-density platforms (9041-13091 EntezIDs) and high-density platforms (17779-20389 EntrezIDs).

Fig 2 shows the influence of sample size on the proportion of DEPs in both real and simulated data. In the simulated datasets (Fig 2A), an increase in sample size results in an increase in the proportion of DEPs for TAPPA and all the multivariable methods (TopologyGSA, Clipper, DEGraph). For each of these methods, we observed a breakpoint (sample size) beyond which the proportion of DEPs stabilised. For TopologyGSA and Clipper, this breakpoint was at 68 samples, with 94.9% and 93.4% DEPs, respectively. For the complete dataset (344 samples), DEGraph and TAPPA identified 94.2% and 68.7% of pathways to be differentially expressed, respectively. On the other hand, SPIA, PRS and CePa reported a rather stable proportion of differentially expressed pathways across all sample sizes (medians between 4.7% and 14.2%). Interestingly, there is a trend of decreasing number of DEPs with increasing sample size in CePa.

Similar observations were made in the analysis of real datasets from the three real data collections. Across all data collections, the highest proportion of DEPs was observed in Clipper (median: 92.5%), followed by TopologyGSA (median: 73.7%), DEGraph (median: 48.0%) and TAPPA (median: 36.1%). CePa, SPIA and PRS reported the smallest median proportion of DEPs (27.9%, 16.5% and 13.9%, respectively). Results for individual disease collections are shown in (Fig 2B). Although the Gene Overexpression Data Collection comprised of relatively small datasets (Table 3), multivariable methods still reported a large proportion of DEPs, similar to the case of generally larger datasets in the Breast Cancer Data Collection. The smallest dataset (with overexpressed c-Src) had the lowest proportion of DEPs in all methods.

**Table 3:** Overview of the data collections.

Data Collection	Number of datasets	Sample size			Number of gene IDs		
		Median	Min	Max	Median	Min	Max
Gene Overexpression	3	17	17	20	23 521	23 521	23 521
Breast Cancer	27	129	49	856	13 091	2 780	20 389
Disease-Control	36	21	8	153	20 535	12 438	20 535

The Breast Cancer Data Collection contained datasets of various microarray platform sizes (probes representing between 2 780 and 20 389 unique Entrez IDs). For *competitive* methods (SPIA, PRS and CePa), the statistical significance of the differential expression of a pathway depends on the set of genes measured in the experiment. A smaller number of genes outside a pathway leads to reduced variability of the random sets of DEGs which results in lower probability of extreme pathway-statistic and, as consequence, higher p-value. Hence, we split the collection into low-, medium- and high- density platform datasets, based on the number of unique EntrezIDs their probes mapped to (from 2780 to 5486 EntrezIDs for low-density, 9041 to 13091 EntrezIDs for medium-density and 17779 to 20389 EntrezIDs for high-density platforms) (Fig 2C and S1 Fig). Indeed, all the competitive methods reported fewer DEPs in the datasets from low-density platforms. On the other hand, one *self-contained* method—DEGraph also reported fewer DEPs. In DEGraph, each pathway is divided into connected components which contain only the measured genes. In case of low-density microarray platform, this results in the small size of the individual components which tend to have higher *p*–values.

The Disease-Control Data Collection contained small to medium size datasets in which patients with various diagnoses were compared to healthy controls. The proportion of DEPs varied greatly between datasets from this collection (Fig 2B). However, when we divided the datasets into cancer-related and non-cancer-related, all the methods reported more DEPs for the cancer-related datasets (Fig 2D). We hypothesised that this was a consequence of the larger number of differentially expressed genes (it is known that tumours have highly deregulated gene expression in comparison to healthy tissue). The proportion of DEPs as a function of the number of DEGs is shown in S2 Fig. Indeed, the percentage of DEPs depended on the number of DEGs in multivariable methods and TAPPA, but not in SPIA, CePa and PRS. Since in ORA methods (SPIA, PRS, CePa), fixed thresholds were used to identify DEGs, we assessed the effect of three thresholds (*p* < 0.05, *p* < 0.01 and *p* < 0.001) on the proportion of DEPs (S4 Fig). For stricter thresholds (*p* < 0.01 and *p* < 0.001), in all methods, the number of DEPs increased with increasing sample size, as one would expect based on statistical properties of hypothesis testing. For *p* < 0.05, however, this trend holds only until a breakpoint in sample size, which is method specific: between 85-120 samples in CePa, between 222-257 samples in PRS and between 257-291 samples in SPIA. After the breakpoint, the number of DEPs rapidly decreases for *p* < 0.05.

To study the effect of pathway size, we divided pathways into small (<35 nodes) and large (≥35 nodes) (following [29]). S3 Fig shows the median *p*-value of pathways within each group as a function of dataset sample size for individual methods. Large pathways achieved lower median *p*-values in comparison to small pathways, independently on the dataset sample size, except PRS. In PRS, we observed the opposite effect starting at 137 (40%) samples. In multivariable methods, median *p*-values decreased very rapidly with increasing sample size, dropping below 0.01 at 33 (10%) and 51 (15%) for Clipper and TopologyGSA.

### Experiment 2: Type I error rate

For all methods, the observed type I error rate was close to the expected 5% threshold, except for CePa (12.8%), see Table 4 and S5 Fig.

**Table 4:** Type I error rates: For each method the number (N) and the proportion (%) of rejected hypotheses out of 1000 tested is shown.

Method	Rejected hypotheses N(%)
SPIA	30 (3.0%)
PRS	38 (3.8%)
Clipper	45 (4.5%)
TopologyGSA	47 (4.7%)
DEGraph	55 (5.5%)
TAPPA	57 (5.7%)
CePa	128 (12.8%)

### Experiment 3: Effect of mean expression, difference in expression and topology of a single gene

In this experiment, we studied the effect of group-specific increase of expression of single genes in three selected pathways (increments of 0.1 to 2 in log2 fold change with step size 0.1 in 200 simulated datasets). The influence of a gene was quantified as a proportion of identified differentially expressed pathways across all simulations and increments. For simplicity, we divided the gene influence into five categories: very low influence (0%-20% DEPs), low influence (20%-40% DEPs), medium influence (40%-60% DEPs), high influence (60%-80% DEPs) and very high influence (80%-100% DEPs) (S6 Fig), respectively.

An induced change in a single gene had a much stronger influence on the results of multivariable methods than on the results of univariable methods. The median proportion of DEPs (combined across all induced differences) for multivariable methods was 82.5% for TopologyGSA, 82.3% for Clipper and 42.7% for DEGraph, compared to 29.3% for PRS, 25.9% for CePa, 15.4% for TAPPA and 12.8% for SPIA.

We further examined the effect of relative change of gene expression between the groups, the effect of gene mean expression and the effect of gene topology in a pathway (S6 Fig).

**Fig 3:**
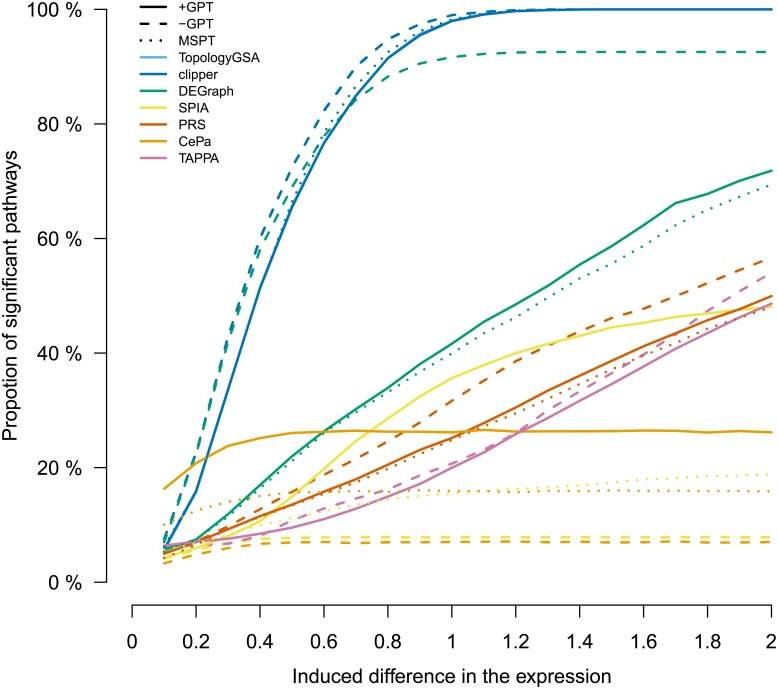
Proportion of differentially expressed pathways (DEPs) combined across all genes as function of the induced change. The proportion of differentially expressed pathways combined across all tested genes in the Non-small cell lung cancer pathway at different induced expression changes. Each line represents one method. Results were very similar for TopologyGSA and Clipper, and the respective lines are overlapping. Solid lines refer to pathway topology from graphite package (+GPT), dashed to pathway topology from graphite package without interactions (-GPT) and dotted to method-specific pathway topology (MSPT).

Fig 3 shows the proportion of DEPs across all genes in the Non-small cell lung cancer pathway as a function of the induced change, for each method separately. TopologyGSA and Clipper were very sensitive to the increase in the induced log2 fold-change of a gene. The higher the fold change, the higher the proportion of DEPs. In fact, both methods marked 96% of the simulations as DEPs at log2FC = 1. In all the other methods, the effect of the increased induced change was less dramatic, although monotone, except CePa that reached its plateau at the induced change of 0.6 (28.3%).

The influence of gene topology was in agreement with methods’ algorithms (S6 Fig). In TopologyGSA and Clipper, all the tested genes had a high or very high influence on the detection of DEPs, regardless of their topological properties (Table 5). The proportion of DEPs was instead correlated with mean expression of the individual genes. The mean expression had no significant effect on the proportion of DEPs in other methods. In DEGraph, the genes with the highest influence were those without incoming interactions (root nodes). In SPIA, the most influential genes had none or only neutral (e.g. binding) incoming interactions and many downstream genes. In PRS, most of the genes had low influence on the pathway detection, except for RIG-I-like receptor signalling pathway, which contained four genes with medium influence. One of these genes was a common subunit of two multiprotein complexes. We observed a correlation of the gene influence with the number of gene interactions in PRS and TAPPA (Table 5). Although the number of interactions is one of the centralities (see S1 Text, section Materials and methods) used in CePa, the most influential genes were the nodes with the highest betweenness centrality.

**Table 5:** Spearman’s correlations coefficients between the gene influence and the number of interactions stratified by interaction type.

	Pathway								
	Bacterial invastion of epithelial cells			Non-small cell lung cancer			RIG-I-like receptor signaling pathway		
	Interaction type			Interaction type			Interaction type		
Method	Incoming	Outgoing	Both	Incoming	Outgoing	Both	Incoming	Outgoing	Both
TopologyGSA	0.434	0.149	0.368	0.102	-0.123	-0.005	0.413	-0.063	0.239
Clipper	0.437	0.153	0.374	0.103	-0.120	-0.002	0.414	-0.059	0.244
DEGraph	-0.399	0.145	-0.264	-0.608	-0.158	-0.446	-0.585	-0.113	-0.477
SPIA	-0.153	0.278	0.096	-0.127	0.070	0.023	0.041	0.314	0.208
PRS	0.220	0.861	0.779	0.355	0.826	0.779	0.610	0.713	0.917
CePa	0.325	0.394	0.648	0.373	0.207	0.493	0.563	0.782	0.916
TAPPA	0.161	0.273	0.403	0.543	0.536	0.747	0.653	0.584	0.873

### Experiment 4: Effect of overexpression of multiple genes

Here, we assessed the combined impact of overexpression of multiple genes (gene sets), regardless of the possible topological motif. In all methods, the number of DEGs in a pathway positively correlated with the number of DEPs. Within the same gene set size, the influence of a gene set increased with the cumulative effect of individual genes as measured in Experiment 3 (S7 Fig).

### Experiment 5: Effect of overexpression of topological motifs

In this experiment, we overexpressed three, four and five genes, respectively, representing one of the 18 topological motifs present in the Non-small cell lung cancer pathway (see S1 Text). Similarly to the previous experiments, the proportion of DEPs increased with the induced change and with the number of genes in the motif.

**Fig 4:**
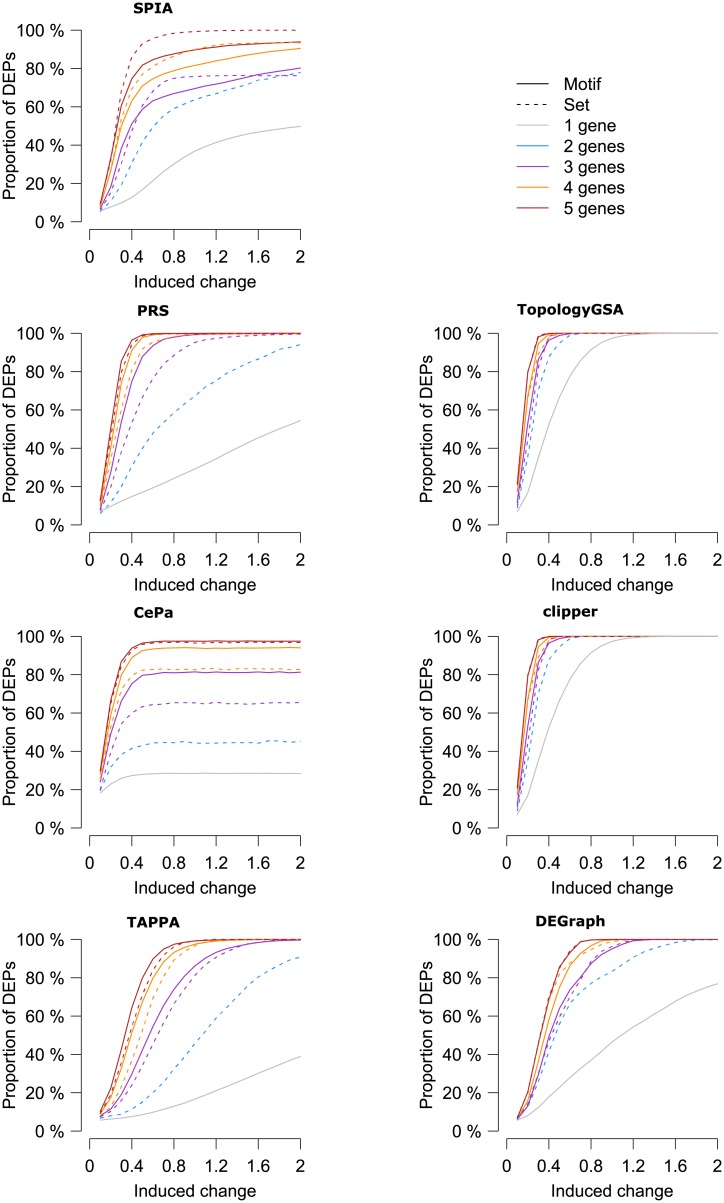
Comparison of the effect of expression change in a single gene, multiple genes and topological motifs. Combined influence of single gene, multiple genes and topological motifs on the proportion of differentially expressed pathways (DEPs) at varying induced expression changes is displayed. Sets of multiple genes and topological motifs are shown in the dashed and solid lines of the same color, respectively.

For the multivariable methods, we did not observe the influence of the motif on the proportion of DEPs when compared to the gene set effect from Experiment 4 (Fig 4). In all univariable methods, except SPIA, motif overexpression resulted in the increased proportion of DEPs in comparison to gene set overexpression. This difference in overexpression was independent of the number of overexpressed genes for TAPPA but diminished with the increasing number of overexpressed genes in PRS and CePa. In contrast, motif overexpression resulted in the decreased proportion of DEPs in SPIA in comparison to gene set overexpression.

**Fig 5:**
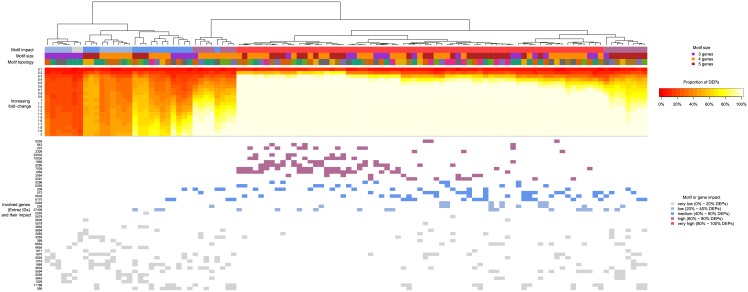
Effect of topological motifs in SPIA. Proportions of differentially expressed pathways (DEPs) for individual motifs (columns) at variable induced log2 fold-changes (rows) are displayed as a heatmap. Color bars on the top show influence of the motif, its size and topology (see S1 Text for details). Note, that colors used for motif topology are unique only among motifs of the same size. The bottom panel shows the influence of the genes in a representation of a topological motif as discovered in Experiment 3.

The effect of the motifs in the context of previous findings and the motifs’ properties (size, topology, the sum of effects of individual genes) is shown as a heatmap with information from Experiment 3 overlaid (S8 Fig, Fig 5). The heatmap shows clustered proportions of DEPs at different increments of log2 fold-changes (rows) in all tested topological motifs (columns). The proportion of DEPs increased with the induced change, and this effect separated the analysed motifs into multiple clusters. We categorised the motifs based on their overall effect (the proportion of DEPs from all the simulations and induced changes). We were also further interested to see how the clusters correlated with the size (3, 4 or 5 genes) and the topology of the motif. For all methods but TopologyGSA and Clipper, we observed a clustering of the motifs according to motif size (S8 Fig). Since Experiment 4 showed that effect of multiple genes is directly dependent on the sum of effects of individual genes, we plotted the effect of individual genes (as measured in Experiment 3) involved in the individual topological motifs in the panel below the heatmap. Here, the gene-specific influence is indicated by color (white means gene was not present in the motif). Clearly, in all methods, the impact of topological motif positively correlated with the impact of individual genes of the motif (S8 Fig).

### Experiment 6: Identification of target pathways

In this experiment, for each real dataset we identified a pathway that was related to the disease or a biological problem and, in an ideal situation, this pathway should be detected as differentially expressed with very low *p*-value in comparison to other pathways.

During the analysis, we encountered multiple method-specific problems that resulted in the impossibility to analyse all available pathways. First, TopologyGSA requires the dataset to have more samples than the number of genes in the largest clique of the pathway and this condition was met only by several pathways. For large datasets, such as SUPERTAM_HGU133A from the Breast Cancer Data Collection (N = 856 expression profiles), we were unable to run TopologyGSA on 80GB RAM machine. DEGraph encountered similar but less frequent problems due to the singularity of pooled covariance matrices.

S9 and S10 Figs, and Tables 4 and 5 in S1 Text show results of the target pathway *p*-values and ranks in the Disease-Control Data Collection and Breast Cancer Data Collection. The results from the Gene Overexpression Data Collection can be found in S11 Fig. Since target pathways are unique for each dataset from this collection, they were not suitable for trend estimation.

Overall, multivariable methods assigned lower *p*–values and ranks to the target pathways than univariable methods. In the Disease-Control Data Collection, the target pathway was tested by TopologyGSA in only ten out of 36 datasets, of which nine times it was reported as differentially expressed. In contrast, the ranks from the DEGraph method were the highest in multivariable methods and the second largest in all methods. PRS and CePa reported consistently low median *p*-values (0.031 and 0.034, respectively) and low median ranks (19.5 and 25.5, respectively). Amongst univariable methods, the highest median *p*-value and rank of target pathways were observed in SPIA and TAPPA. In the Breast Cancer Data Collection datasets, the aim was to detect differentially expressed pathways between the estrogen receptor positive (ER+) and estrogen receptor negative (ER-) group. The set of target pathways therefore comprised of four pathways with estrogen receptor genes: Endocrine and other factor-regulated calcium reabsorption, Estrogen signalling pathway, Prolactin signalling pathway and Thyroid hormone signalling pathway. Since estrogen receptor plays different roles in these pathways and therefore harbours different topological ‘importance’, results for individual pathways from topology-based pathway analysis may vary. For all these pathways, all multivariable methods (TopologyGSA, Clipper, DEGraph) again reported very low *p*-values and ranks. From the univariable methods, TAPPA returned the lowest median *p*-values (except Estrogen signalling pathway) and the highest ranks (except Endocrine and other factor-regulated calcium reabsorption pathway). The lowest median *p*-values and ranks of all target pathways amongst remaining univariable methods were observed in CePa. SPIA reports lower *p*-values and ranks than PRS only for the Endocrine and other factor-regulated calcium reabsorption pathway. Estrogen receptor is one of the root nodes and has a medium influence on this pathway in SPIA (47% DEPs) and only low influence in PRS (22% DEPs). On the other hand, Prolactin signalling pathway is the least significant by SPIA, and the estrogen receptor is a leaf node in this pathway with very low influence (3.5% DEPs). In the original experiments of the Gene Overexpression Data Collection, an overexpression of three genes (c-Myc, H-Ras, c-Src) was induced experimentally via adenoviral infection. The fold change of the perturbed genes ranged from 2.38 to 5.29 (S1 Text). 15, 40 and 14 target pathways were identified, for c-Myc, H-Ras and c-Src, respectively. The results of the analysis of this collection are summarized in Table 6. TopologyGSA was able to analyse only the Bladder cancer pathway, which was detected as differentially expressed. Clipper identified all target pathways as differentially expressed. Results of DEGraph, PRS, CePa and TAPPA, varied greatly between the three sets of pathways, ranging from 21% to 80% target pathways as differentially expressed. All univariable methods reported a higher percentage of target pathways as differentially expressed in the dataset with deregulated c-Myc in comparison to other datasets. When individual target pathways were assessed separately, DEGraph and univariate methods agreed on differential expression of the most biologically relevant pathways (S11 Fig).

**Table 6:** Proportion of significant target pathways in the Gene Overexpression Data Collection.

Overexpressed gene in the target pathway	Method						
	SPIA	PRS	CePa	TAPPA	TopologyGSA	Clipper	DEGraph
c-Myc	7/15 (46.7%)	8/15 (53.3%)	12/15 (80%)	10/15 (66.7%)	0/0	14/14 (100%)	3/9 (33.3%)
H-Ras	14/40 (35.0%)	6/40 (15.0%)	16/40 (40.0%)	9/40 (22.5%)	1/1 (100%)	39/39 (100%)	18/23 (78.3%)
c-Src	5/14 (35.7%)	3/14 (21.4%)	3/14 (21.4%)	3/14 (21.4%)	0/0	14/14 (100%)	3/6 (50%)

### Experiment 7: Effect of the exclusion of topological information

To assess the effect of exclusion of topological information, we studied the effect of individual genes on the proportion of differentially expressed pathways in the simulated datasets. We hypothesised that, in the non-topological setting, individual genes influence the final result equally. We applied the non-topological variants of the methods on both simulated (from Experiment 3) and real (from Experiment 6) datasets and Non-small cell lung cancer pathway was used as a model pathway for simulated data. Then we quantified the effect of genes in simulated datasets and computed the corresponding *p*–values and ranks of target pathways. The results were compared to the results obtained in Experiment 3 and the Experiment 6 (Fig 3).

The effect of the individual genes in simulated data is shown in S12 Fig. In TopologyGSA and Clipper, no difference between the topological and non-topological variant of the method was found. In all other methods, we did observe, in agreement with our hypothesis, the equal redistribution of the effect of the genes across the pathway in the non-topological variant. For DEGraph and PRS, the non-topological variant resulted in an overall increase of the individual gene effects, while in CePa and SPIA, the individual effects of the genes diminished. In the Disease-Control Data Collection (S9 Fig), we observed increased *p*-values and ranks for target pathways in PRS and CePa and decreased *p*-values and ranks for DEGraph and SPIA. No effect of exclusion of topological information was found in TAPPA, TopologyGSA and Clipper. Note that the median *p*-value of the target pathway was below 0.2 in all methods regardless pathway topologies. In PRS, the median *p*-value raised from 0.031 in the topological variant to 0.055 in the non-topological variant. In the Breast Cancer Data Collection (S10 Fig), we observed the pathway-specific effect of the exclusion of pathway topologies in SPIA where *p*-values increased only in the pathway in which estrogen receptor is one of the root nodes (Endocrine and other factor-regulated calcium reabsorption) and decreased in other pathways. In all estrogen receptor containing pathways, we observed increased *p*-values in CePa and decreased in PRS. No difference was observed in multivariable methods.

### Experiment 8: Effect of pre-processing of pathway topologies

**Fig 6:**
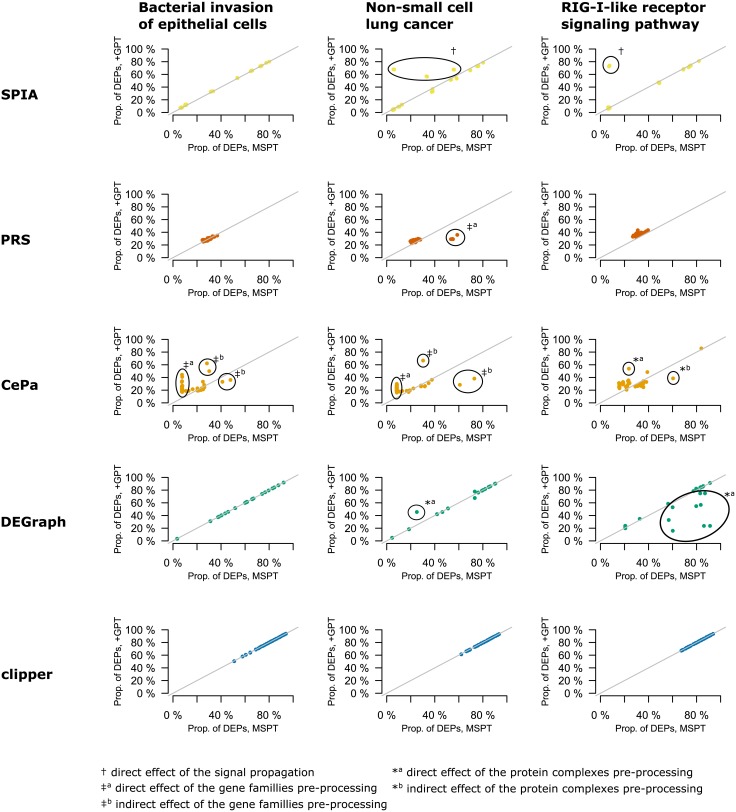
Effect of pre-processing of pathway topologies on simulated data—Overexpression of single gene. Each point represents a single gene. Only genes common for pathway topologies from graphite package (+GPT) and method-specific pathway topologies (MSPT) are displayed. Points on diagonal represent genes with the same influence in +GPT and MSPT. Points below (above) diagonal represent genes with higher (lower) influence in MSPT.

To assess the effect of pre-processing of pathway topologies (methods’ original pre-processing MSPT vs graphite pre-processing +GPT), we first compared effects of the individual genes in model pathways (Fig 6). The main differences between +GPT and MSPT were in the pre-processing of multisubunit protein complexes, gene families and interactions related to non-gene product nodes (e.g. small chemical compounds). These differences had a direct effect on individual genes by changing their properties or an indirect effect on the genes by altering the distribution of a particular property in a pathway. No difference in the effects of individual genes was observed in Clipper. In the DEGraph’s original pathway topology (MSPT) there were no interactions between subunits of multiprotein complexes. These interactions were introduced in graphite (S1 Text, [28]) pathway topologies (+GPT). In consequence, the genes whose products were subunits of multiprotein complexes had a different effect in MSPT compared to +GPT (see Fig 6, RIG-I-like receptor signalling pathway and Non-small cell lung cancer pathway). There were no protein complexes in the Bacterial invasion of epithelial cells pathway, so the gene effects were the same. In PRS, we observed a clear difference in the effect of individual genes only in the Non-small cell lung cancer, where a group of six genes had approximately two times higher effect in MSPT compared to +GPT. In this pathway, two nodes involved each of these genes—either as a member of two different gene families or a single node and a member of a gene family. In MSPT of PRS, gene families were processed into combined nodes (S1 Text), hence possibly increasing the effect of genes present in multiple nodes. We observed complex differences in gene effects between +GPT and MSPT for CePa. In CePa’s MSPT, gene families and protein complexes are pre-processed into combined nodes, thus decreasing their degree centralities (if they interacted with other families or complexes) or decreasing the total number of nodes in a pathway resulting in the reduced influence of family members or subunits of protein complexes. At the same time, both the influence and the degree centrality of the genes interacting with these families was reduced. However, other genes gained importance as consequence of the different distribution of centralities or pathway topology. SPIA-specific pre-processing of pathway topologies did not propagate perturbations of individual genes through as many interaction types (including compound-mediated interactions) as in graphite. Therefore, in MSPT, the number of genes with high influence was reduced.

In both the Breast Cancer Data Collection and Disease Control Data Collection, with the agreement to the individual gene overexpression experiment, we observed increased *p*-values in CePa; slightly increased ranks in DEGraph and decreased *p*–values in PRS and no difference in *p*-values in Clipper (S10 Fig). For SPIA, we observed no difference in both *p*–values and ranks in agreement with the individual gene overexpression experiment only in the Breast Cancer Data Collection and decreased *p*–values and ranks in the Disease Control Data Collection.

## Discussion

We presented a series of eight controlled experiments designed to gauge the suitability of a number of topological pathway analysis methods to various analytical scenarios. Since topological information can be used in different ways and for different goals, in our study, we decided to focus on methods that (i) aim to detect differentially expressed pathways between two groups of interest, (ii) use a priori known pathway structures (topologies) and (iii) model each pathway separately. We described the performance of the selected methods on both simulated and real datasets.

We studied the methods’ behavior from several perspectives: the sample size, pathway size, platform density, effect size, number of differentially expressed genes, gene topologies, platform density, gene sets and their topological motifs, the inclusion of topology information in the method’s algorithm and different strategies for pre-processing of pathway topologies. The influence of the tested variables was assessed by comparison of the proportion of differentially expressed pathways, their p-values and ranks.

Table 7 shows the overall evaluation of the compared methods and summarises the most important observations from our experiments.

**Table 7:** Overall assessment of the compared methods.

Parameter	SPIA	PRS	CePa	TAPPA	TopologyGSA	Clipper	DEGraph
Median proportion of DEPs in real datasets	16.5%	13.9%	27.9%	36.1%	73.7%	92.5%	48.0%
**Effect on proportion of DEPs due to**							
Increasing sample size	→	→	slowly ↘	↗	rapidly ↗	rapidly ↗	↗
Increasing pathway size	↑	↑ ↓	↑	↑	↑	↑	↑
DEGs threshold *p* < 0.001	↑	↑	↑	NA	NA	NA	NA
DEGs threshold *p* < 0.01	↑	↑	↑	NA	NA	NA	NA
DEGs threshold *p* < 0.05	↑ ↓	↑ ↓	↑ ↓	NA	NA	NA	NA
Single DEG [% DEPs]	12.8%	29.3%	25.9%	15.4%	82.5%	82.3%	42.7%
**Characteristics of the most influential genes**							
Crutial node property	root node	connected DEGs	betweenness	degree	mean expression	mean expression	root node
Incoming interactions	!!	-	!!	!!	-	-	!!
Outgoing interactions	!!	!!	!!	!!	-	-	-
Mean expression	-	-	-	-	!!	!!	-
**Impacts of individual genes as observed on simulated data**							
+GPT [% DEPs]	4.2 - 82.6	23.4 - 44.0	16.9 - 86.4	3.5 - 71.2	51.2 - 94.5	51.2 - 94.5	4.0 - 92.6
-GPT vs. +GPT	↓	↑	↓	↕	↑	↑	↑
MSPT vs. +GPT	↓	↑	↕	NA	NA	→	↑
**Preferred scenario for hypotheses generation**							
Number of DEGs	Many	Many	Many	Any	Few	Few	Few
Sample size	Any	Any	Any	Any	Small	Small	Small
Pathway of interest	Any	Any	Any	Any	Small	Small	Small
Experiment scale	Genome	Genome	Genome	Any	Any	Any	Any

→ - stable, ↘ - decrease, ↗ - increase, ↑ - higher, more, ↓ - lower, less, ↑↓ - trend changes at certain point, NA - not applicable, root node - node without incoming interactions, !! - important, - - not important, ↕ - both effects observed

In all the compared methods, large pathways (> 35 genes) were assigned lower *p*-values than small pathways. Also, as expected, when a pathway contained more differentially expressed genes it was more often detected as differentially expressed. The number of differentially expressed genes usually surpassed their topological influence. None of the methods showed a preference for a particular differentially expressed topological motif.

The most striking difference was found between multivariable and univariable methods. Multivariable methods (TopologyGSA, Clipper and DEGraph) overall reported larger proportions of differentially expressed pathways in comparison to univariable methods (SPIA, PRS, CePa and TAPPA). Although all tested multivariable methods are derived from Hotelling’s *T*^2^ statistic, they differed significantly in their performance. TopologyGSA and Clipper assigned very low *p*-values and ranks to all the target pathways. However, this seems to be the result of overall low specificity, since they reported many other pathways (if not all) as differentially expressed. These methods were also sensitive to the increase in the sample and pathway size, the number of differentially expressed genes and the mean gene expression. The higher the increase, the lower the *p*–values and the larger the proportion of differentially expressed pathways, independent of the platform density. These findings indicate that in the scenario where (i) many differentially expressed genes are expected (e.g. cancer-related experiments); (ii) the dataset contains more than a few tens of samples (> 68 samples in our experiments); (iii) a pathway contains a gene with at least a subtle random change in the expression, the pathway will be identified as significant. This behavior agrees with the *self-contained* nature of the methods, which is known to have higher sensitivity. However, many differentially expressed pathways identified by these methods might be false positives and therefore not useful for selection of biological hypotheses for further research. Interestingly, in TopologyGSA and Clipper, the exclusion of the topological information made no difference in the results. Therefore, despite well-established mathematical background (Graphical Gaussian models), these methods do not appear to fit the definition of topology-based methods for identification of differentially expressed pathways.

In contrast, DEGraph detected fewer differentially expressed pathways compared to TopologyGSA and Clipper, suggesting higher specificity. At the same time, in DEGraph the influence of individual genes was related to the pathway topology. DEGraph was less sensitive to sample size, pathway size or the number of differentially expressed genes. The performance of the non-topological variant of DEGraph was similar to the TopologyGSA and Clipper with or without topology. Different pathway pre-processing strategies had only limited influence on both DEGraph and Clipper (not assessed for TopologyGSA).

Univariable ORA methods SPIA, PRS and CePa, assigned low *p*-values only to some of the target pathways depending on the topological properties of differentially expressed genes in the pathway. This behaviour suggests higher specificity and stronger dependency on the topological information. These methods were less sensitive to the effects of sample size, pathway size, number of DEGs or thresholds used to identify differentially expressed genes. However, with increasing number of differentially expressed genes in a pathway, the effect of gene topology became less important. Due to the competitive nature of SPIA, PRS and CePa, these methods reported less differentially expressed pathways on low-density platforms. The univariable methods also exhibited higher sensitivity to the pre-processing of pathway topologies. Hence they can be considered true representatives of the topology-based pathway methods. Pre-processing of protein complexes, gene families and interactions involving non-gene products (metabolites such as PIP3) was the key factor in methods’ performance and influence of the individual genes. Although, our results suggest that, for PRS and CePa, the method-specific pathway pre-processing seams to be more appropriate and should be preferred to graphite’s approach, further research is needed to identify an optimal pre-processing strategy for the compared methods. For instance, gene family members may be incomplete, and thus the observed increased influence of a gene which is a member of two different gene families may not be biologically sustained. Also, members of a gene family are seen as interchangeable regarding signal transduction, while each subunit of a protein complex is necessary for complex assembly and biological function. Therefore the unified approach, as used in method-specific pathway pre-processing, may not be optimal. The TAPPA [11] method stands out with its unique algorithm—a gene expression profile is being transformed into a pathway-level expression profile. Pathway-expression profiles were then analysed with traditional statistical methods (e.g. Mann-Whitney test for identification of differentially expressed pathways between two groups). As a consequence, this method is suitable also for applications with a complex experimental design. The sensitivity and specificity of TAPPA seemed to be well balanced. Amongst univariable methods, it was the most sensitive to sample size and usually identified most of the differentially expressed pathways. However, the proportion of differentially expressed pathways was never as high as in TopologyGSA or Clipper. At the same time, the method performance depended on the topological properties of the deregulated genes.

### Guidelines for method selection

The increased sensitivity of multivariable methods (mainly TopologyGSA and Clipper) makes them ideal candidates for pathway analysis of experiments, where subtle changes in expression or a small number of differentially expressed genes between two conditions are expected—e.g. as in the case of tumor samples which contain a significant proportion of non-tumoral tissue (such as supporting stroma), thus confounding and diminishing measured signal of the gene expression. Since multivariable methods do not use lists of differentially expressed genes based on pre-defined thresholds but work with a complete list of the tested genes, they can be applied even in cases where none or very few genes are significant after statistical testing (for instance due to small sample size). The results of these methods, however, must be taken with caution and the significance of a pathway of interest must be interpreted in the context of all the results to ensure it is not just a consequence of overall low specificity of the method. To control for low specificity of the result, we recommend using DEGraph.

**Fig 7:**
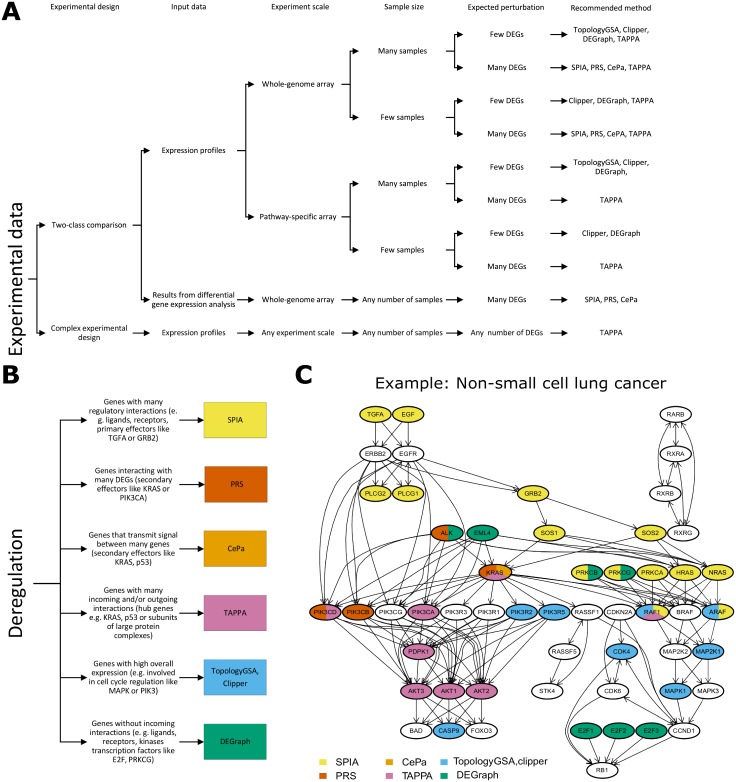
Guide to selection of topology-based pathway analysis method. (A) Recommended methods for specific scenarios based on experimental design, available input data, platform density, sample size and expected number of differentially expressed genes. (B) The most important deregulated genes in particular methods. Individual methods prefer different genes as the most important for pathway deregulation, and these preferences represent another factor for optimal method selection. The genes are defined mostly by their topological properties (e.g. number of interactions). Examples of genes must be interpreted within specific pathway (p53 signalling pathway for p53, Non-small cell lung cancer for others), and specific pathway pre-processing (graphite). (C) An illustrative example of the most important genes in the Non-small cell lung cancer pathway from KEGG database as available in the graphite package.

Univariable methods are not sensitive to the sample size or the number of differentially expressed genes in the datasets. Their ability to identify particular pathway as differentially expressed is highly dependent on the topological properties of the deregulated genes, the inclusion of the topological information and the pre-processing of the pathway topologies. Univariable methods are recommended in most applications and especially when the biological hypothesis aims at a pathway where genes of certain topological properties (biological function) are expected to be affected (see below and Fig 7B). However, since SPIA, PRS and CePa are ORA methods, they require at least some differentially expressed genes, and their applicability on datasets with very subtle changes in gene expression can be limited (in contrast to multivariable methods). On the other hand, if the differentially expressed genes occupy in the pathway the “correct” topological positions, the topological properties of the methods help to categorize this pathway as significant despite a small overall number of differentially expressed genes in the pathway. TAPPA, in contrast, being the FCS method, is a good choice for applications with a limited number of differentially expressed genes overall. Since in TAPPA the most important genes are those with many interactions, pre-processing of gene families and protein complexes must be carefully considered as their expansion into individual members or subunits may unintentionally increase their effect.

Based on our results we propose some guidelines for optimal method selection based either on (i) design of the experiment (comparison type, input data type, the platform density, sample size, expected number of differentially expressed genes)—Fig 7A; or (ii) selected (preferred) deregulation type—Fig 7B. Note, that the presence of many differentially expressed genes in a pathway surpasses topological effect of individual genes. Fig 7C shows an example of the most influential genes in the Non-small cell lung cancer pathway based on graphite pre-processing of topologies (+GPT). In SPIA, CePa, TAPPA and DEGraph, we colored all the genes with the highest influence as defined in Experiment 3 for each method. Details of the topological as well as biological properties of the most influential genes are described in the S1 Text. In TopologyGSA and Clipper, the most influential genes have the highest overall expression (usually related to the cell cycle regulation [30]). In DEGraph and SPIA, the genes without incoming interactions have the largest impact. These genes are often represented by ligands, receptors, or transcription factors (E2F family dissociating from pRB). On the other hand, genes interacting with many other genes (e.g. secondary effectors, such as PIK3CA or KRAS) have the highest influence in PRS, CePa and TAPPA.

The observed differences between topological methods should be considered when the results of pathway analyses are to be compared across experiments in which different methods were used to detect differentially expressed pathways. Currently, SPIA is the most often cited method (282 citations from Web of Science Core Collection as of 14 February 2017). The other compared methods were mainly used in methodological publications, in which the general concepts were compared to the new method and only very rarely in applications.

## Conclusion

We performed one of the largest studies of topology-based pathway analysis methods published to date. In this study, we compared seven methods that aim to detect differentially expressed pathways from expression data employing a priori known pathway topologies in their algorithm. The methods were ranked according to their sensitivity to sample and pathway size, ability to detect target pathways, the proportion of differentially expressed pathways, benefit from incorporating topological information and sensitivity to different pathway pre-processing strategies. We also verified type I error rates and described the influence of overexpression and topological properties of a single gene or gene sets on the detection of a pathway as differentially expressed by the selected methods.

We demonstrated that multivariable self-contained methods are very sensitive to the changes in gene expression within a pathway leading to the uninformative identification of over 90% pathways as differentially expressed. As a consequence, a significant result can be easily obtained for a particular pathway. On the other hand, univariable methods (mostly competitive) were less sensitive to subtle changes in gene expression but exhibited stable performance over a wide range of scenarios and benefited from the inclusion of topological information.

Finally, we proposed guidelines for method selection based on a number of variables connected to experimental design as well as biological hypotheses. Overall, we recommend any of the multivariable approaches to be used mainly for applications with small sample size and subtle changes in gene expression, whereas univariable methods should be preferred for genome-scale applications with large changes in gene expression. The pre-processing strategy for pathway topologies must be carefully considered for univariable methods, and further research is required to identify an optimal pre-processing strategy.

## Supporting information

S1 Text Details of the selected methods and used real data collection. (PDF)

S1 Fig Effect of the number of Entrez IDs. Proportion of DEPs depending on the number of Entrez IDs for datasets from Breast Cancer Data Collection. Each point represents one dataset.
(PDF)

S2 Fig Effect of the number of DEGs. Proportion of DEPs depending on the number of DEGs for datasets from Disease-Control Data Collection. Each point represents one dataset.
(PDF)

S3 Fig Effect of the pathway size. (PDF)

S4 Fig Effect of the thresholds used for DEG detection. (PDF)

S5 Fig Distribution of *p*-values from Experiment 2. (PDF)

S6 Fig Summarization of the Experiment 3. Dependence of the proportion of DEPs on the difference in expression induced between groups, the gene mean expression and its postion. In SPIA, neutral interactions were drawn in grey.
(PDF)

S7 Fig Effect of expression change in randomly selected multiple genes on the proportion of differentially expressed pathways. Sets of 2, 3, 4 and 5 genes were randomly selected from Non-small cell lung cancer pathway. Each circle represents one of those sets. Expression of genes in the set was modified with increments of 0.1 to 2 with step size 0.1 in 200 simulated datasets. Border color indicates number of genes in the set. Vertical axis shows combined influence of the genes (proportion of differentially expressed pathways across all increments and datasets). Horizontal axis corresponds to sum of the influence of individual genes. Pie color (from grey to blue and red) represents the influence of a single gene (see Experiment 3 for details).
(PDF)

S8 Fig Summarization of the Experiment 5. Heatmaps of the proportion of DEPs for all compared methods.
(ZIP)

S9 Fig P-values and ranks of the target pathways—Disease-Control Data Collection details. Boxplots of *p*-values and rank of the estrogen receptor-containing pathways in Disease-Control Data Collection. Ranks are based on *p*-values. Pathway with the lowest *p*-value has rank 1. All pathways with the same *p*-value recieved same rank. The rank was incremented by one between subsequent *p*-values.
(PDF)

S10 Fig P-values and ranks of the target pathways—Breast Cancer Data Collection details. Boxplots of *p*-values and rank of the estrogen receptor-containing pathways in Breast Cancer Data Collection. Ranks are based on *p*-values. Pathway with the lowest *p*-value has rank 1. All pathways with the same *p*-value recieved same rank. The rank was incremented by one between subsequent *p*-values.
(PDF)

S11 Fig P-values of the target pathways—Gene Overexpression Data Collection details. Heatmaps of *p*-values of the overexpressed oncogene-containing pathways in Gene Overexpression Data Collection. Pathways are ordered by the number of methods in which they are differentially expressed (*p* < 0.05).
(PDF)

S12 Fig Effect of individual genes in non-topological variants of the methods. (A) Proportion of differentially expressed pathways for different genes and the difference in expression induced between groups. (B) Dependence of the proportion of differentially expressed pathways on the difference in the gene position. In the non-topological variants of the methods (-GPT) we observed reduced proportion of differentially expressed pathways and loss of its dependence on gene postion in all methods except TopologySGA an Clipper.
(PDF)
