# Supplementary material for: A critical comparison of topology-based pathway analysis methods
Source: PLoS One. 2018 Jan 25;13(1):e0191154. doi: 10.1371/journal.pone.0191154 (PMC5784953; doi:10.1371/journal.pone.0191154)
Supplement: S1 Text — (PDF) [file pone.0191154.s001.pdf]

Introduction

Following the categorization of the GSEA methods, topology-based pathway analysis methods can be divided based on three main criteria: (i) the null hypothesis; (ii) the identification of differentially expressed genes (DEGs) before pathway analysis and (iii) the number of variables in the model.

Depending on the type of the null hypothesis we distinguish *competitive* and *self-contained* methods [19]. The null hypothesis of a *competitive* method expects the genes in a pathway to be at most as often differentially expressed as the genes outside the pathway (the remaining genes measured in the experiment). Methods testing this hypothesis typically use gene randomization in the assessment of statistical significance and cannot be applied in an experiment that only measured the expression of genes in a particular pathway. The null hypothesis of the *self-contained* methods expects none of the genes from the pathway to be differentially expressed and use sample randomization in the assessment of the statistical significance. These methods can be used for both genome-wide as well as pathway-specific experiments but require sufficient sample size for sample randomization.

Identification of differentially expressed genes before the pathway analysis distinguishes between *over-representation analysis (ORA)* and *functional class scoring (FCS)* methods as described in [33]. Usage of ORA methods depends on a priori generation of a list of differentially expressed genes (DEGs), usually by application of arbitrarily chosen thresholds. Then the DEGs are mapped on the pathway, and their proportion is statistically assessed. Traditionally, ORA is represented by methods based on 2×2 contingency tables, coupled with the hypergeometric model or Fisher's exact test and competitive hypothesis. However, since both proportion and position of the DEGs is statistically evaluated in the topology-based pathway analysis, both competitive and self-contained hypothesis can be tested. On the other hand, FCS methods (like GSEA [72]) do not depend on the application of any thresholds and transform gene-level statistics of all measured genes into a pathway-level statistic. Both competitive and self-contained hypotheses can be tested with FCS methods.

Independently on the categories above, we can further distinguish *univariable* and *multivariable* methods. Known univariable topology-based methods, typically, increase the weight of the differentially expressed genes as a function of their topological properties (position in the graph, the proximity of other differentially expressed genes etc.) [32, 3, 20] or transform the expression profile of each sample separately from gene-level to pathway-level [18]. Multivariable topology-based methods use either Gaussian Graphical Models [49] or Fourier analysis on graphs [30] combined with Hotelling's T^2 statistic for detecting differentially expressed pathways.

Material and methods

We performed eight distinct experiments to provide comprehensive insight into the topology-based pathway analysis methods. In these experiments, we examined the influence of the number of parameters on the results obtained by topology-based pathway analysis methods (proportion of differentially expressed pathways (DEPs), distribution of p-values or p-value-based ranks).

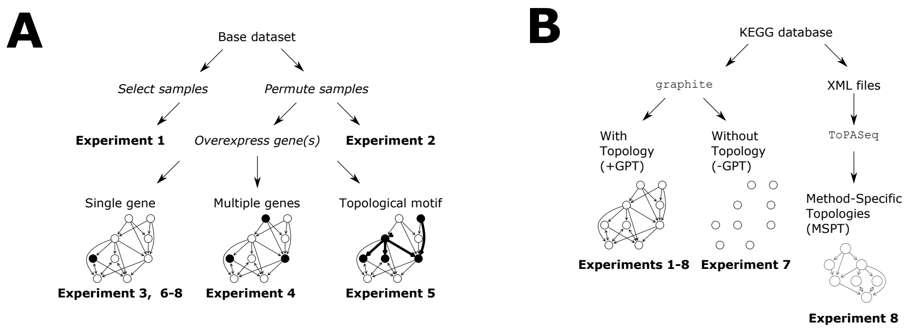

Figure 1: **Overview of the generation of the simulated dataset and pathway topologies used in individual experiments**
(A) Generation of the simulated datasets. A base dataset was selected from the Breast Cancer Data Collection. Subsets of this dataset were used in the Experiment 1. Samples were randomly assigned to two groups in experiments 2-8 and overexpression of a single or multiple genes was induced in Experiments 3-8. (B) Pathway topologies used in individual experiments. Human KEGG pathways as provided by `graphite` package were used in all experiments. The interactions between nodes were removed for Experiment 7. For Experiment 8 we created a new set of pathway topologies by method-specific pre-processing of manually downloaded KGML files from the KEGG database.

Each experiment E is formally defined as a tuple $E = (X_{p\times(n_1+n_2)}, y, PT, \theta, m)$, where X is a normalized $\log_2$-transformed gene expression data matrix of expression profiles of p genes (rows) and $n_1 + n_2$ samples (columns), n_1 and n_2 denote number of samples in two compared groups, y is a vector of 1's and 2's assigning samples into the groups, PT is a set of pathway topologies, θ is the threshold used for detection of DEGs and m is a topology-based pathway analysis method. Pathway topologies are represented as graphs $G = (V, E)$, where V is a set of vertices/nodes represented by products of genes, and E is a set of edges representing interactions between them. Both real and simulated datasets were used in the individual experiments.

Datasets

In our study, we used both real and simulated datasets. The real gene expression microarray datasets were obtained from public sources and merged into three collections: Gene Overexpression Data Collection [34, 9], Breast Cancer Data Collection [22] and Disease Control Data Collection [8, 7]. Individual datasets are described in separate section *Real datasets.* These datasets were used in Experiments 1, 6, 7 and 8.

Since the proper statistical distribution of the pathway expression data is unknown, we decided to use a real dataset (a dataset from Breast Cancer Data Collection denoted as VDX) as a base for the generation of simulated data. The simulated datasets were used in all experiments. Fig 1A displays generation of simulated datasets for particular experiments.

Pathways and their topologies

Pathway topologies can be obtained from the existing implementation of the selected methods, which provide either already pre-processed pathway topologies (original implementations of PRS, CePa, TopologyGSA and Clipper and `graphite`) or a tool for pre-processing of the manually downloaded files (original implementation of SPIA and DEGraph and `ToPASeq` from version 1.9.1). `graphite` employs a unique pre-processing strategy of gene families, multi-protein complexes and chemical compounds which allows signal propagation through compound-mediated interactions, but differs from the original method's implementation or publication.

We used human pathways from the KEGG database as the source of pathway topologies. For our comparison, we used `graphite`'s pre-processed pathways as a default set of pathway topologies. In `ToPASeq` one can choose either `graphite` pre-processed pathways (+GPT) or pathway pre-processing as in the original implementation (MSPT) (if available) and hence evaluate the effect of different pre-processing strategies. Fig 1B shows the generation of pathway topologies for individual experiments.

Experiments

Experiment 1: effect of sample size, pathway size, platform density and number of differentially expressed genes

Experiment 1 aimed to study the following effects: sample size, pathway size and significance threshold for detection of differentially expressed genes. We used both real and simulated datasets. In total, 380 simulated datasets were created by 20 random selections of 5%, 10%, 15% ... 95% of the samples in each clinical group resulting in 19 different sample sizes of 16, 33, 51 ... 326. To study the effect of pathway size, we further, as in [76], split pathways into small ($<$ 35 genes) and large ($\geq$ 35 genes). Finally, three thresholds for detection of DEGs were compared: $p < 0.05$, $p < 0.01$ and $p < 0.001$. The methods were compared in terms of proportion of DEPs from all the tested pathways.

Experiment 2: type I error rate

In this experiment, we assessed type I error rate. We generated 1000 simulated datasets in which we randomly assigned samples of the base dataset into two groups. Only one pathway was used to fix the effect of the pathway size and type, the Non-small cell lung cancer from +GPT. The methods were compared in terms of relative and absolute number of datasets in which this pathway was identified as differentially expressed.

Experiments 3-5: effect of overexpression of a single gene, multiple genes and topological motifs

The main goal of Experiments 3 to 5 was to assess the effect of mean expression, difference in expression and topology of a single gene (Experiment 3), multiple random genes (later referred to as *gene set* or *set*, Experiment 4) and genes connected in particular topological motif (later referred to as *topological motif* or *motif*, Experiment 5) in one out of the two compared groups. The topological

motifs studied in Experiment 5 are visualized in 2. For this, due to high computational costs we used only the first 200 simulated datasets from Experiment 2 and added a constant to one or more genes in one of the groups. The constant varied from 0.1 to 2 with step size 0.1 sampling the range of common log2 fold-changes in real datasets. We selected three model pathways for Experiment 3: Non-small cell lung cancer, RIG-I-like receptor signaling pathway and Bacterial invasion of epithelial cells. These pathways differ in the number of pathway components (2,1 and 1, respectively) and presence of protein complexes (yes, yes and no, respectively). For Experiments 4 and 5 we used only Non-small cell lung cancer pathway.

In Experiment 3 we modified the expression of each gene present in the pathway individually (152 genes in total, 144 unique) whereas in Experiment 4 we created and subsequently altered expression of genes from 40 randomly selected gene sets (10 gene sets for each size: 2, 3, 4 and 5). In the model pathway (Non-small cell lung cancer) we identified topological motifs with 3, 4 and 5 nodes, as follows: first, we generated all 3-,4- and 5-node combinations and then we removed all combinations which did not induce a connected subgraph. The induced connected subgraphs represented 10, 40 and 157 isomorphism classes (topological motifs). Then for each motif, we identified only vertex and edge disjoint matches and discarded motifs with less than five matches (representations). A match of a motif is an induced subgraph of the target pathway which is isomorphic to the motif. We obtained 18 topological motifs with 110 matches in total.

In all Experiments 3-5 we calculated proportion of DEPs and used it as score to divide genes, gene sets and topological motifs into 5 categories that describe their influence on the pathway significance: very low (0% - 20% DEPs), low (20% - 40% DEPs), medium (40% - 60% DEPs), high (60% - 80% DEPs), very high (80% - 100% DEPs).

Experiment 6: effect of target pathways

In Experiment 6 we evaluated the ability of the methods to detect potentially relevant biological pathways. First, we applied each method on a full set of human KEGG pathways on the real datasets (from GODC, BCDC and DCDC) and created pathway lists in ascending order according to their p-values. Then, for each dataset, according to its experimental design, we identified *target pathway(s)* as pathways which were very likely to be differentially expressed. Here, we would like to emphasize the word "likely", since real ground truth information is lacking. For the GODC datasets, target pathways were those containing oncogene overexpressed in the particular dataset. In BCDC, estrogen receptor-containing pathways were target pathways for all datasets. Since oncogenes or estrogen receptors are of different importance (relevance) for individual target pathways, we expected to observe a pathway-specific effect in topology-based pathway analysis methods. Each dataset from DCDC was related to a specific disease for which a matching pathway exists in KEGG, for example for a dataset of the Non-small cell lung cancer patients and healthy controls, the Non-small cell lung cancer pathway was the target pathway. Together, in DCDC we identified 15 target pathways in 36 datasets (each dataset had only one target pathway, but one pathway could be target pathway in multiple datasets). Since DEPs are usually identified by an application of arbitrary threshold on p-values

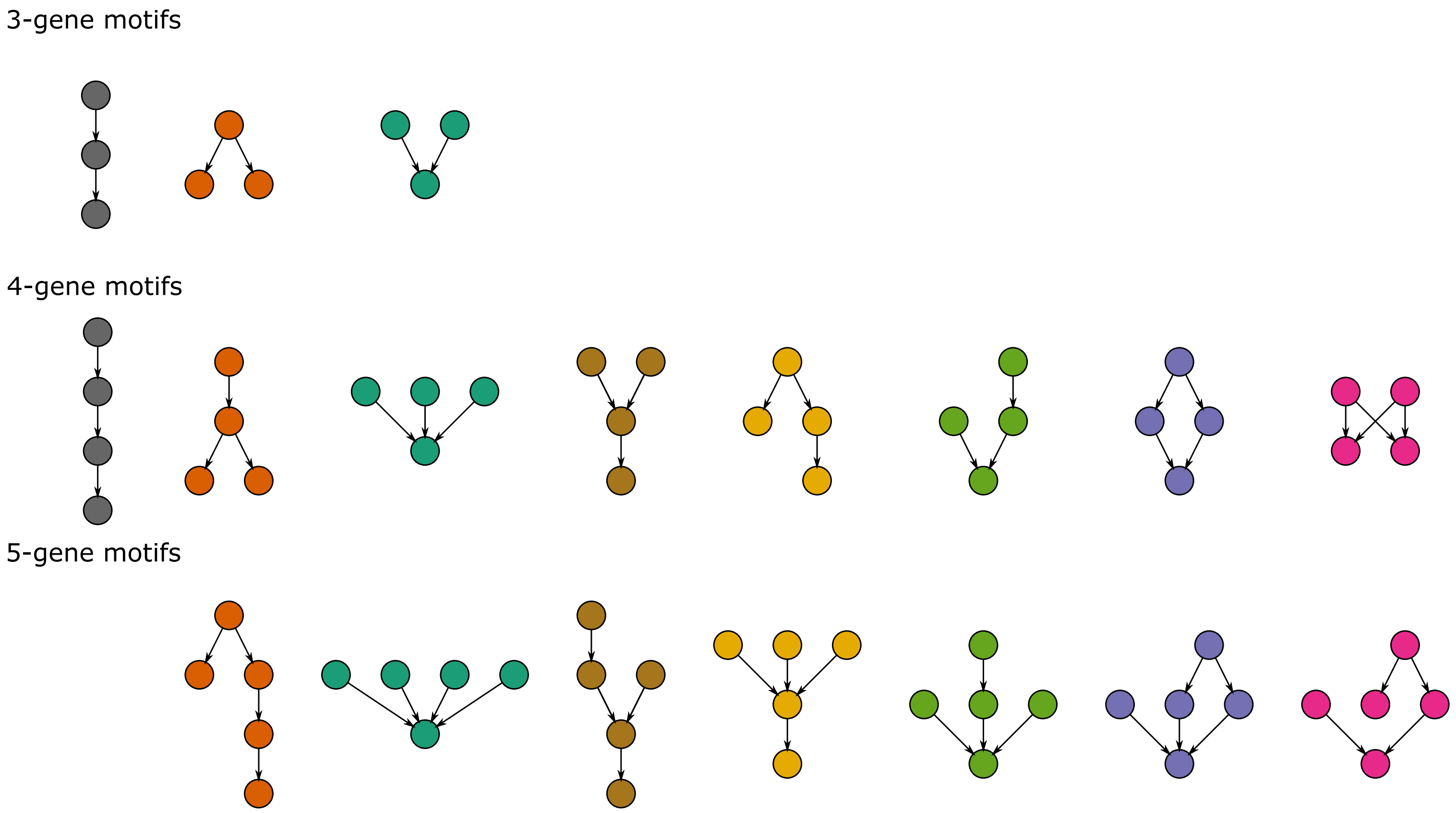

Figure 2: **Topological motifs studied in Experiment 5**

(eventually q-values) or as the top pathways in a ranked pathway list, we looked at the distribution of the p-values and ranks of the target pathway. We expected target pathways to have low p-values and ranks. We combined all target pathways in DCDC data collection and separately assessed target pathways for BCDC and GODC. Moreover, we used the same approach as in Experiment 3 to quantify the influence of estrogen receptor in estrogen receptor-containing pathways for SPIA and PRS.

Experiment 7: effect of exclusion of topological information

In this experiment we tested whether the inclusion of topological information had any influence on the method's performance. Instead of comparing topology-based pathway analysis method with an arbitrarily chosen enrichment-based method, we created and used non-topological (enrichment-based) variants of the compared methods as follows. First, we decomposed the algorithm into individual steps. Then we identified the step incorporating topological information and later decoupled this step from the algorithm (see *Non-topological variants of selected method* for details).

We applied the non-topological variants of the methods on both real (from Experiment 6) and simulated (from Experiment 3) datasets. Non-small cell lung cancer pathway was used as a model pathway for simulated data. Then we quantified the effect of genes in simulated datasets and calculated p-values and ranks of target pathways. The results were compared to the results obtained in Experiment 3 and Experiment 6 (the topological variants).

Experiment 8: effect of pre-processing of pathway topologies

In Experiment 8 we studied the effect of the pre-processing of pathway topologies. In contrast to all previous experiments, here, pathway topologies were created from freely available manually downloaded files in KEGG Markup Language format (KGML). A KGML file is coded in XML-like language and contains information about 3 distinct entities: (i) nodes (genes, protein complexes, gene families, compounds, pathways, etc., same gene can be represented by several nodes); (ii) relations (interaction between two proteins/gene products or protein and compound) and (iii) reactions (between a substrate and a product involving an enzyme, only in metabolic pathways). These entities were processed as stated by the authors of compared methods in the original implementations or publications, or as explained in personal communication. The approaches taken for various pathway elements are shown in Fig 3. While in CePa all types of nodes are preserved, only gene products are kept in the other methods. All methods combine multiple instances of the same molecules into a single node. Gene families and protein complexes are expanded as either OR or AND group in Clipper, DEGraph and SPIA, but preserved as a combined node in PRS and CePa. All methods retrieve protein-protein interactions and expression regulation interactions. DEGraph, SPIA and PRS do not propagate interactions through a compound. These interactions are recovered only in Clipper and CePa. The sequence of reactions is also reflected in a randomly oriented relation, which is retrieved in Clipper, DEGraph and CePa. The proper orientation of the relation is recovered only in PRS. All metabolic pathways are omitted in SPIA. Examples of two particular pathways can be found in Figs 4

and 5. Note, that the strategy used for Clipper matches the original publication of the `graphite` package [62]. However, after its first release, authors decided to discard nodes with no interactions.

This set of pathway topologies is later referred to as Method-Specific Pathway Topologies (MSPT). Two methods (TopologyGSA and TAPPA) did not provide sufficient information about topology pre-processing or use third-party databases, hence were excluded from this experiment.

As in Experiment 7, both real (from Experiment 6) and simulated datasets (from Experiment 3) were analyzed and Non-small cell lung cancer pathway from MSPT was used as a model pathway for simulated data. For each method, we correlated the influences of individual genes in MSPT with the genes influences in +GPT. p-values and ranks of target pathways from MPST were compared to those from +GPT.

Overview of the compared topology-based pathway analysis methods

We compared a variety of univariable and multivariable topology-based pathway analysis methods. Here, we capture only the most essential steps of the methods' algorithms; more details can be found in the original papers or section *Topology-based pathway analysis methods.*

In general, the univariable methods (SPIA [75, 15, 32], PRS [3] CePa [20]) share the following common steps. First, a list of DEGs is identified and their log fold-changes calculated. DEGs are most often identified by moderated t-test [67] and application of a threshold on the resulting p-values. In CePa, only the information about the significance of a gene is used. Then, DEGs are mapped to the pathway topology and, for each gene, topology-dependent weights are calculated. Log fold-changes and the weights are later combined into a pathway-level score, and a gene permutation approach is used to assess statistical significance. The topology-dependent weights of genes reflect their importance and are method specific. In SPIA, the most important genes are considered to be the receptors (genes with no incoming interactions). In PRS, the most important genes are those that are connected to a large number of differentially expressed genes. In CePa, the importance of the genes is connected to its centrality (a function that assigns a real value to each node in a graph). Amongst selected centralities, two are of particular interest: (i) degree, which equals to the number of interactions of a given node and is also employed in other univariable methods and (ii) betweenness, which equals to the number of shortest paths passing through a given node. Methods also differ greatly in their gene permutation strategy. SPIA and CePa generate random DEGs for each pathway separately. In SPIA, the number of DEGs in a pathway equals the number of DEGs in the original dataset and DEGs are randomly selected from pathway genes. In CePa, random DEGs are obtained by repeated Bernoulli trials where each gene has the probability of being differentially expressed equally to the proportion of DEGs in the dataset. The number of DEGs in a pathway is variable between permutations and different from the number of DEGs in the original dataset. In PRS, the random DEGs are generated for all pathways in one run. From the set of genes in the dataset, the same number of DEG as in the original dataset is picked randomly. With this approach, the number of DEGs for a pathway is variable across permutations. The TAPPA [18] method stands

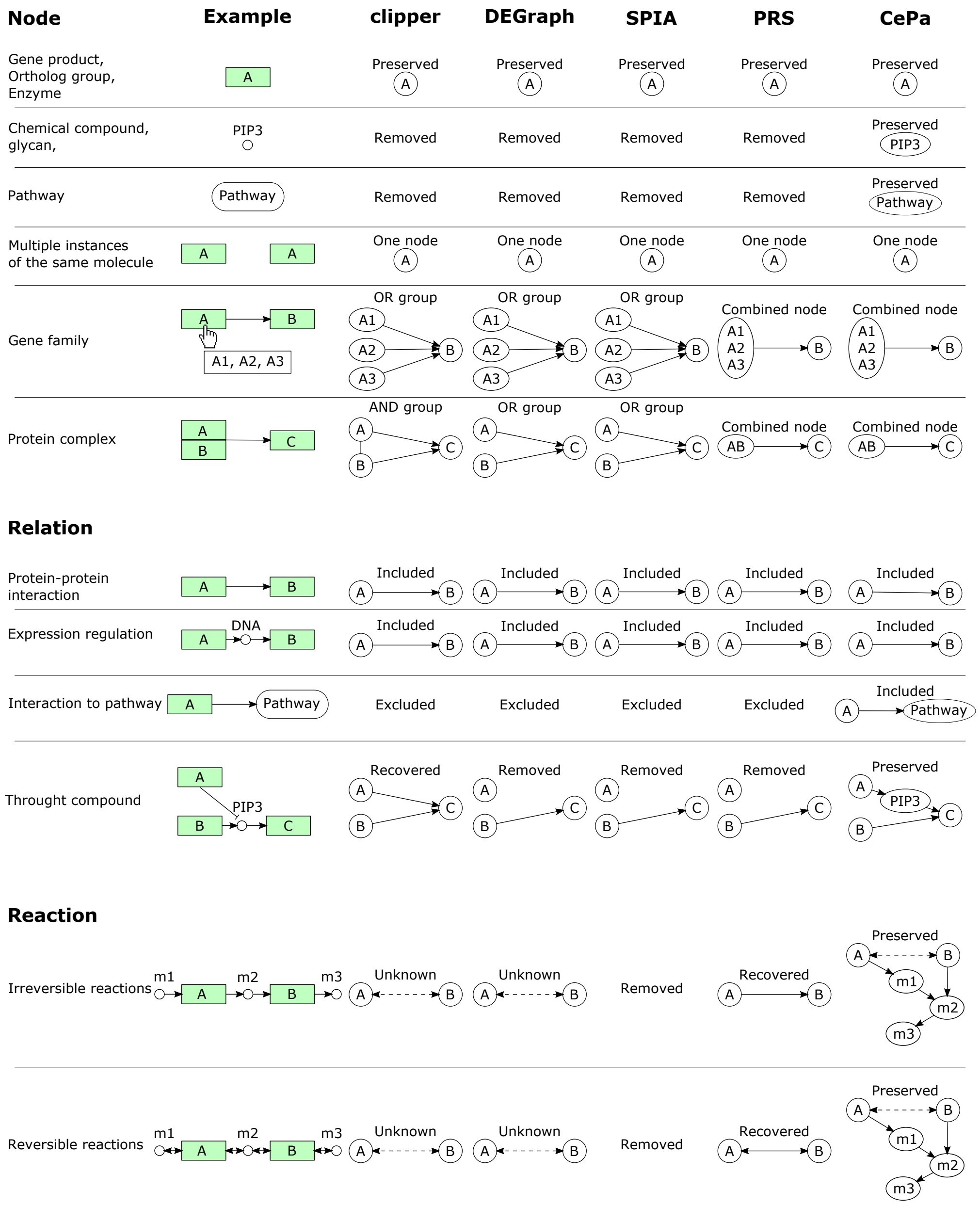

Figure 3: **Processing of selected elements from KEGG pathways.**

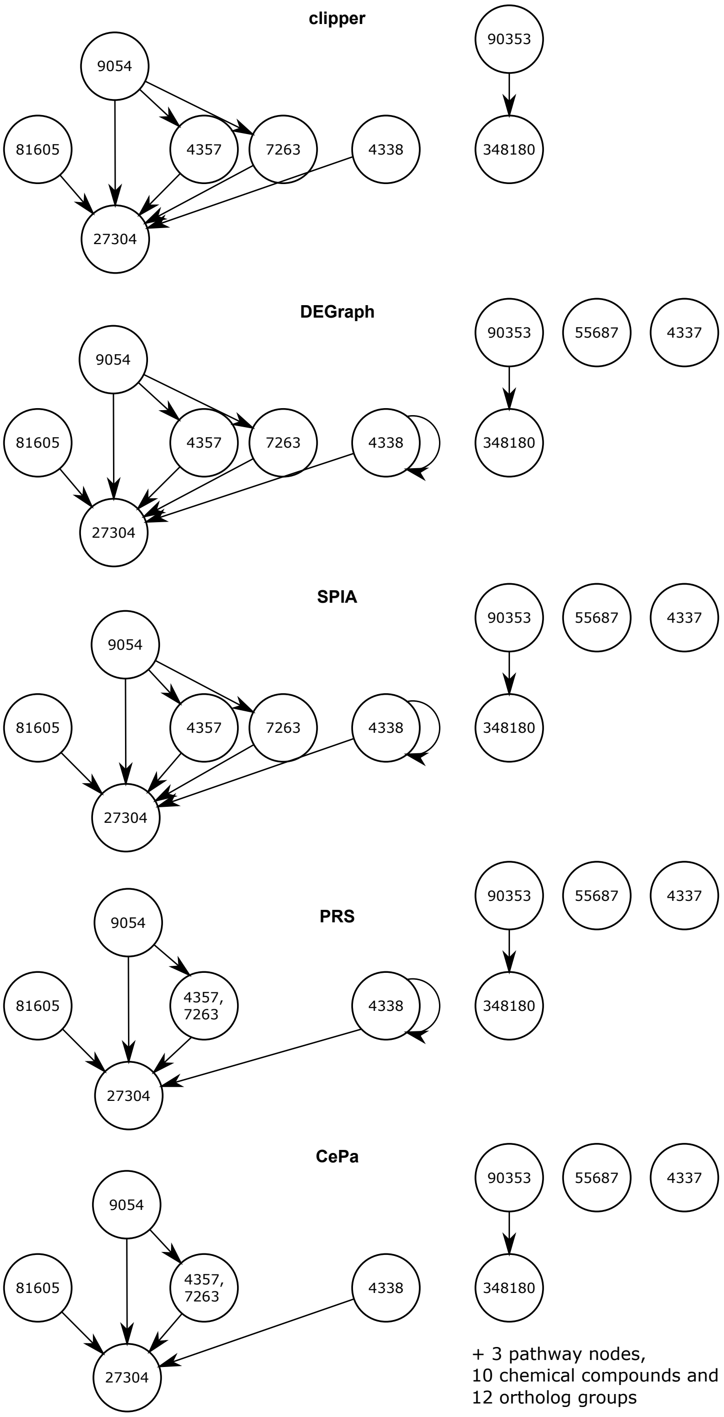

Figure 4: **Method-specific pathway pre-processing of signaling pathway - Sulphur relay system.**

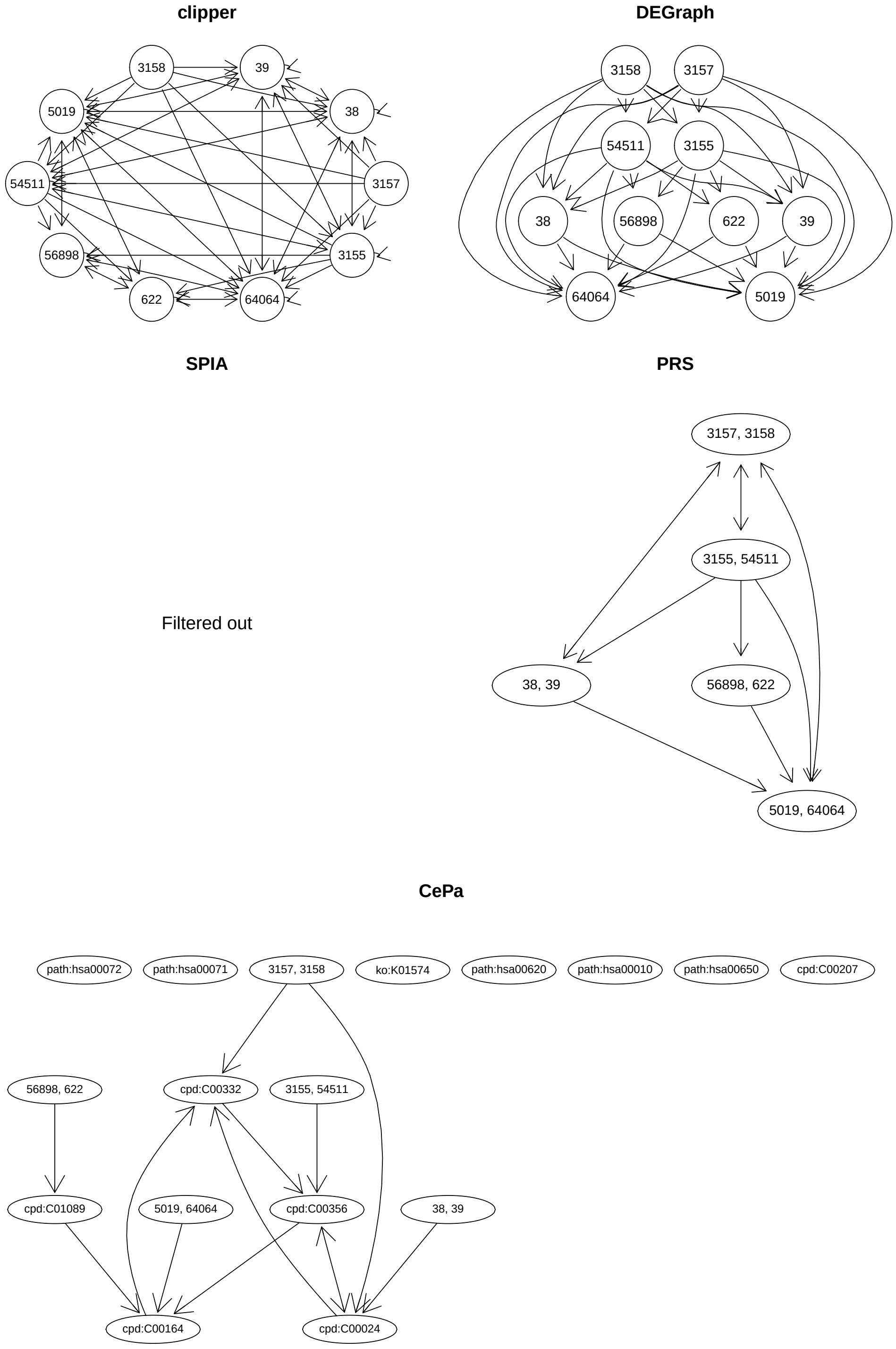

Figure 5: **Method-specific pathway pre-processing of metabolic pathway - Synthesis and degradation of ketone bodies.**

out with its unique approach. Self-interactions (self-loops) for all genes are being inserted, and a pathway connectivity index is being calculated to transform a gene expression profile into a pathway-level expression profile. Depending on the experimental design one needs to later decide on an appropriate test statistics. In our experiments, we aimed to detect differentially expressed pathways between two groups of interest, and therefore we selected Mann-Whitney test.

The underlying model of the multivariable methods (TopologyGSA [49], Clipper [48] and DEGraph [30]) is

$$\{X_1^1, \ldots, X_{n_1}^1\} \in \mathbb{R}^p, X_i^1 \sim \mathcal{N}_p(\mu_1, \Sigma_1), i = 1, \ldots, n_1$$

$$\{X_1^2, \ldots, X_{n_2}^2\} \in \mathbb{R}^p, X_i^2 \sim \mathcal{N}_p(\mu_2, \Sigma_2), i = 1, \ldots, n_2,$$

where X_i^1 denotes expression profile of sample i in the first clinical group which contains n_1 samples, (analogously for X_i^2 and n_2), $\mathcal{N}_p$ denotes p-dimensional multivariate normal distribution. The methods differ in their assumption about covariance matrices. TopologyGSA and Clipper are based on Graphical Gaussian Models and assume the inverse of the covariance matrix as a symmetric positive definite matrix with null elements corresponding to the missing edges in the graph after moralization and triangulation. A missing edge reflects conditional independence of two genes given the other genes in a pathway. The inverse covariance matrices are estimated via Iterative Proportional Scaling algorithm [35]. The Clipper method adds James-Stein-type shrinkage estimator [64] to assure convergence in the case the number of nodes of the largest clique of the graph exceeds the number of samples. A clique is a subset of nodes of an undirected graph such that every two distinct nodes in the clique are adjacent. The null hypothesis $\mu_1 = \mu_2$ is tested with Hotelling's T^2 statistic which is compared to the empirical null distribution created by sample permutations. Note, that this description matches the methods original implementations and not necessarily the original papers. In the original articles, authors propose two-step approach in which the test statistic for the null hypothesis $\mu_1 = \mu_2$ depends on the decision on the null hypothesis $\Sigma_1 = \Sigma_2$.

In DEGraph, both covariance matrices are expected to be equal [30]. A smooth distribution shift (similar for pairs of connected nodes, coherent with activation or inhibition) of the gene expression on the graph is assumed. Two groups are compared in terms of the first k components of the graph-Fourier basis (or in the original space after filtering out k high-frequency components). Hotelling's T^2-test is used for the comparison and a p-value is assigned to each connected component of the pathway topology. In our analyses, the largest component is used as a representation of the whole pathway.

Real datasets

The Gene Overexpression Data Collection (GODC, Table 1) is available from Gene Expression Omnibus (ID: GSE3151). This dataset contains 55 expression profiles of human primary mammary epithelial cell cultures. In the original experiment [34, 9], expression of 5 well-known oncogenes (β-catenin, E2F3, c-Myc, H-Ras and c-Src) and control (green fluorescent protein - GFP) was artificially upregulated by recombinant adenoviruses. The data were obtained via GEOquery package, quantile normalized and aggregated to Gene Symbol selecting the probe with highest SD and no missing values. Because expression profiles of

β-catenin and E2F3 did not confirm overexpression, those profiles were excluded from further analyses. Profiles with selected oncogene (7-10 profiles) and GFP (10 profiles) were combined into separate datasets. Pathway node identifiers were converted to the Gene Symbols. Due to experimental design, pathways that contain the deregulated oncogene are of particular interest. Each oncogene is present in more than ten pathways (22, 23 and 55 for c-Src, c-Myc and H-Ras respectively).

The Breast Cancer Data Collection (BCDC) is a collection of 35 breast cancer datasets (Table 2), which was downloaded from `http://compbio.dfci.harvard.edu/pubs/sbtpaper/data.zip`. In the archive, each dataset is stored as a separate .RData file containing three objects: a gene expression data matrix (normalized, log2-transformed), clinical data (e.g. estrogen receptor status, progesterone receptor status, human epidermal growth factor 2 status, node status, tumor size, age, grade etc.) and an annotation matrix. Within three molecular markers, estrogen receptor status was available for most of the samples and therefore it was chosen as the main clinical factor. Six datasets (DFHCC3, EMC2, FNCLCC, MCCC, MUG and NCCS) were excluded due to missing estrogen receptor status data. Additionally, datasets UCSF and NKI contained missing values in most of the probes and therefore were also excluded from the further analysis. The remaining 27 datasets (median sample size: 129, minimum: 49, maximum: 856) were processed as GODC. However, expression data were aggregated to Entrez ID, to which node identifiers of pathway topologies were translated, too.

Packages `KEGGdzPathwaysGEO` and `KEGGandMetacoreDzPathwaysGEO` from Bioconductor contain in total 36 preprocessed datasets (Table 3, median sample size: 21, minimum: 8, maximum: 153) from the Gene Expression Omnibus. Since each dataset consists of expression profiles of patients and healthy controls, the collection was named Disease-Control Data Collection (DCDC). Provided datasets cover 15 different diagnoses (malignant, neurodegenerative, metabolic and others) and each of them can be mapped to a single Human Diseases pathway from KEGG (*target pathway*). The datasets were processed as BCDC.

Topology-based pathway analysis methods

TopologyGSA

TopologyGSA [49] is a multivariate method based on the Gaussian Graphical Models. The pathway is represented as a directed acyclic graph which is later moralized and triangulated. Then, the concentration matrices (inverse of the covariance matrices) are estimated via Iterative Proportional Scaling algorithm [35] (IPS) from the sample covariance matrices. This algorithm reduces covariance of gene-pairs which are not connected in the graph while preserving genes variance and covariance of connected gene-pairs. The necessary condition for the existence of the estimate is that the number of samples is greater than the number of nodes of the largest cliques. Then, the question of the equality of the concentration matrices is addressed via likelihood test. If the null hypothesis is rejected, as stated in the original publication, the pooled covariance matrix is estimated according to the Behrens-Fisher problem [4]. Otherwise, the pooled variance is estimated as in Hotellings T^2 test. In both scenarios Hotellings T^2 statistic is used to assess the statistical significance of the differential expression

Overexpresed oncogene/ Dataset	Samples	Platform	Probe(set)s	Gene Symbols	Pathways	fold-change (case/control)
c-Myc	10	23	HG-U133_Plus_2	54 675	23 521	2.382
H-Ras	10	55	HG-U133_Plus_2	54 675	23 521	5.292
c-Src	7	22	HG-U133_Plus_2	54 675	23 521	5.007
GFP	10	0 (control group)	HG-U133_Plus_2	22 283	23 521	

Table 1: Overview of the Gene Overexpression Data Collection

Data Set	Reference	Sample Size	Platform	Probe(set)s	Entrez IDs	ER+ samples	ER- samples
CAL (A-TABM-158)	[11]	118	HG-U133A	22 283	13 091	43	75
DFHCC (GSE19615)	[38]	115	HG-U133_Plus_2	54 675	23 521	45	70
DFHCC2 (GSE18864)	[66, 38, 31]	84	HG-U133_Plus_2	54 675	23 521	53	31
DUKE (GSE3143)	[9]	171	HG-U95	12 625	9 041	57	114
DUKE2 (GSE6861)	[74]	160	U133_X3P	61 359	20 389	123	37
EORTC10994 (GSE1561)	[16]	49	HG-U133A	22 283	13 091	22	27
EXPO (GSE2109)	-	246	HG-U133_Plus_2	54 675	23 521	85	161
HLP (E-TABM-543)	[53]	53	Illumina Human-6 v2	48 701	19 985	28	25
IRB (GSE5460)	[47]	129	HG-U133_Plus_2	54 675	23 521	53	76
KOO	[29]	88	HG-U95	12 625	9 041	15	73
LUND (GSE5325)	[60]	143	Swegene Human 27K RAP UniGene188	26 824	5 486	29	114
LUND2 (GSE5325)	[60]	105	Swegene Human 27K RAP UniGene188	27 648	5 472	60	45
MAINZ (GSE11121)	[65]	200	HG-U133A	22 382	13 091	38	162
MAQC2 (GSE20194)	[57]	230	HG-U133A	22 382	13 091	89	141
MDA4	[41, 24]	127	HG-U133A	22 382	13 091	48	79
MSK (GSE2603)	[52]	99	HG-U133A	22 382	13 091	42	57
NCI	[68]	99	custom made cDNA	7 650	2 780	34	65
PNC (GSE20713)	[12]	88	HG-U133_Plus_2	54 675	20 365	43	45
STK (GSE1456)	[55]	159	HG-U133A and HG-U133B	44 928	18 528	29	130
STNO2	[70]	113	custom made cDNA	7 787	5 427	31	82
SUPERTAM_HGU133A (GSE6532, GSE9191, GSE17705, GSE12093)	[46, 45, 44, 73, 83]	856	HG-U133A	22 283	13 091	140	716
SUPERTAM_HGU133PLUS2 (GSE6532, GSE9191)	[46, 45, 44]	410	HG-U133_Plus_2	54 675	23 521	85	325
TRANSBIG (GSE7390)	[13]	198	HG-U133A	22 283	13 091	64	134
UNC4	[58]	253	multiple Agilent platforms	17 779	17 779	99	154
UNT (GSE2990)	[?, 69]	126	HG-U133A and HG-U133B	44 928	18 528	40	86
UPP (GSE3494)	[50]	247	HG-U133A and HG-U133B	44 928	18 528	34	213
VDX (GSE2034, GSE5327)	[51, 81]	344	HG-U133A	22 283	13 091	135	209

Table 2: Overview of Breast Cancer Data Collection

Dataset	Reference	Sample size	Platform	Probesets	Entrez IDs	Disease samples	Control samples	Target pathway
GSE1145	-	26	HG-U133_Plus_2	54 675	23 521	15	11	Dilated cardiomyopathy
GSE1297	[10]	16	HG-U133A	22 283	13 091	7	9	Alzheimer's disease
GSE14762	[80]	21	HG-U133_Plus_2	54 675	23 521	9	12	Renal cell carcinoma
GSE14924_CD4	[36]	20	HG-U133_Plus_2	54 675	23 521	10	10	Acute myeloid leukemia
GSE14924_CD8	[36]	21	HG-U133_Plus_2	54 675	23 521	10	11	Acute myeloid leukemia
GSE15471	[5]	70	HG-U133_Plus_2	54 675	23 521	35	35	Pancreatic cancer
GSE16515	[56]	30	HG-U133_Plus_2	54 675	23 521	15	15	Pancreatic cancer
GSE16759	[54]	8	HG-U133_Plus_2	54 675	23 521	4	4	Alzheimer's disease
GSE18842	[63]	88	HG-U133_Plus_2	54 675	23 521	44	44	Non-small cell lung cancer
GSE19188	[28]	153	HG-U133_Plus_2	54 675	23 521	91	62	Non-small cell lung cancer
GSE19420	[78]	24	HG-U133_Plus_2	54 675	23 521	12	12	Type II diabetes mellitus
GSE19728	[42]	21	HG-U133_Plus_2	54 675	23 521	17	4	Glioma
GSE20153	[84]	16	HG-U133_Plus_2	54 675	23 521	8	8	Parkinson's disease
GSE20164	[84]	11	HG-U133A	22 283	13 091	6	5	Parkinson's disease
GSE20291	[84, 82]	33	HG-U133A	22 283	13 091	14	19	Parkinson's disease
GSE21354	[43]	17	HG-U133_Plus_2	54 675	23 521	13	4	Glioma
GSE23878	[77]	38	HG-U133_Plus_2	54 675	23 521	19	19	Colorectal cancer
GSE24739_G0	[2, 1]	12	HG-U133_Plus_2	54 675	23 521	8	4	Chronic myeloid leukemia
GSE24739_G1	[2, 1]	12	HG-U133_Plus_2	54 675	23 521	8	4	Chronic myeloid leukemia
GSE32676	[14]	32	HG-U133_Plus_2	54 675	23 521	25	7	Pancreatic cancer
GSE3467	[23]	18	HG-U133_Plus_2	54 675	23 521	9	9	Thyroid cancer
GSE3585	[6]	12	HG-U133A	22 283	13 091	7	5	Dilated cardiomyopathy
GSE3678	-	14	HG-U133_Plus_2	54 675	23 521	7	7	Thyroid cancer
GSE4107	[27]	22	HG-U133_Plus_2	54 675	23 521	12	10	Colorectal cancer
GSE4183	[21, 17]	23	HG-U133_Plus_2	54 675	23 521	15	8	Colorectal cancer
GSE5281_EC	[39, 40]	21	HG-U133_Plus_2	54 675	23 521	9	12	Alzheimer's disease
GSE5281_HIP	[39, 40]	23	HG-U133_Plus_2	54 675	23 521	10	13	Alzheimer's disease
GSE5281_VCX	[39, 40]	31	HG-U133_Plus_2	54 675	23 521	19	12	Alzheimer's disease
GSE6956AA	[79]	10	HG-U133A	22 283	13 091	5	5	Prostate cancer
GSE6956C	[79]	16	HG-U133A	22 283	13 091	8	8	Prostate cancer
GSE7305	[25]	20	HG-U133_Plus_2	54 675	23 521	10	10	Endometrial cancer
GSE781	[37]	17	HG-U133A and HG-U133B	44 928	18 528	12	5	Renal cell carcinoma
GSE8671	[61]	64	HG-U133_Plus_2	54 675	23 521	32	32	Colorectal cancer
GSE8762	[59]	22	HG-U133_Plus_2	54 675	23 521	12	10	Huntington's disease
GSE9348	[26]	82	HG-U133_Plus_2	54 675	23 521	70	12	Colorectal cancer
GSE9476	[71]	63	HG-U133A	22 283	13 091	26	37	Acute myeloid leukemia

Table 3: Overview of Disease Control Data Collection

of the pathway. However, all available implementations use always both the pooled variance and test statistic as in Hotellings T^2 test.

The method is implemented in two R-packages: `topologyGSA` and `ToPASeq`. Implementation in `topologyGSA` relies on `moralize()` function from `gRbase` which no longer supports mixed graphs (graphs with both directed and undirected edges as in `graphite`) and requires directed acyclic graph.

clipper

Clipper [48] is a two-step method. First, the whole pathway is tested in a similar way as in TopologyGSA. In here, the approach is generalized for situations where the number of samples is much smaller than the number of genes (violating requirement of the IPS algorithm) by employing James-Stein-type shrinkage estimators [64] of the covariance matrices combined with the IPS. In the second step, a pathway topology is transformed into a junction tree, and the signal paths with the greatest association with specific phenotype are identified. This second step was beyond the scope of this paper.

DEGraph

DEGraph [30] also belongs to the multivariate methods. It assumes a smooth distribution shift (similar for pairs of connected nodes, coherent with activation or inhibition) of the gene expression on the graph. The smoothness is defined and controlled by spectral analysis of the graph Laplacian. In particular, the eigenvectors of the Laplacian provide a basis of functions which vary on the graph at increasing frequencies (corresponding to the increasing eigenvalues). Two groups can be therefore compared in terms of the first k components of the graph-Fourier basis, or in the original space after filtering out k high-frequency components. Hotelling's T^2-test is used for the comparison, and a p-value is assigned to each connected component of the pathway topology. Low ratio of graph-Fourier to full space p-value indicates a smooth shift.

SPIA

Signaling pathway impact analysis (SPIA) evolved from Pathway-Express by modification of perturbation factor calculation. Both methods were developed by the same research group [75, 15, 32]. In the impact analysis, two independent aspects of the pathway expression are captured by two p-values P_{NDE} and P_{PERT}. The first one refers to the significance of a pathway as provided by conventional gene set analysis method (it is based on the number of DEGs observed in the pathway). The second probability is calculated from the amount of perturbation measured. The two p-values are finally combined into an overall probability

$$P = c - c\ln(c) \text{ where } c = P_{NDE}.P_{PERT}$$

that tests the hypothesis that the pathway is significantly perturbed between two groups.

Interactions are divided into three categories: positive (e.g. activation), negative (e.g. inhibition) and neutral (e.g. binding, phosphorylation). These types are incorporated in the calculation of the gene perturbation which was first done via propagation through the network than via a system of linear equations

from which finally effect size of individual genes was removed. It is assumed that the difference in the expression of the entry nodes has a greater impact on the rest of the pathway than the difference in the expression of the leaf nodes. Also, if the DEGs are localized in a connected subgraph, a higher overall score for pathways is anticipated. Statistical significance of the P_{PERT} is assessed by permutation test in which the same number of DEG are mapped at random location throughout the pathway.

PRS

The Pathway Regulation Score (PRS) [3] is one of the newest approaches that detect significantly deregulated pathways by exploiting both topology and fold-change data. First, the list of DEG is created according to two thresholds: fold-change and a p-value from a simple t-test. PRS for pathway p is defined as

$$PRS_p = \sum_{i=1}^{N} NS_j$$

where NS_j is the node score given as the node value times number of downstream DEG (directly or via other significant nodes, including the starting node itself). The node value is 0 for genes without expression data, 1 for genes with expression data but not significantly deregulated and maximum absolute fold-change if any of the mapped genes is expressed and significantly deregulated. PRS is later normalized for the pathway size and the pathway-specific null distribution. The bias due to pathway size is removed by multiplying PRS by the ratio of the number of DEGs in a pathway to the total number of expressed genes. To characterize the null distribution of the raw values, fold-change values of all genes are permuted and random scores created. The raw values are standardized by mean and SD of permuted scores. The statistical significance is assessed by gene permutation. The p-value is a proportion of normalized random scores greater or equal to normalized raw scores and finally, adjusted via Benjamin-Hochberg method. A pathway detected as significantly differentially expressed has either a sufficient number of DEG, a sufficient combined fold-change of DEGs or DEG are located in densely connected subgraphs.

CePa

Another method that uses topological properties as weights in regular gene set analysis method is called Centrality-based Pathway enrichment analysis [20] or CePa. The weights are based on the centrality measures for the nodes like in-degree, out-degree, in-reach, out-reach and betweenness. The in-degree/out-degree is the number of upstream/downstream nodes directly acting on a given node. The in-reach/out-reach is the largest length of the shortest paths from a given nodes to all upstream/downstream nodes in the pathway. The betweenness is represented by the amount of information streaming through a given node.

The method begins by the determination of the differentially expressed genes and mapping them to the pathways. The pathway score is then defined as

$$S_p = \sum_{i=1}^{N} w_i d_i$$

where $d_i = 1$ if the node i is differentially expressed and $d_i = 0$ otherwise. w_i is a centrality-based weight. To avoid zeros, 1/100 if the minimum non-zero weight is added. The statistical significance is assessed by gene permutations (probability of a gene in a pathway to be DE is the proportion of DEGs in the dataset) and empirical null distribution of the pathway scores. Authors also provide GSA extension.

A significant pathway contains a few highly important differentially expressed genes or many less important ones. If a pathway is significant by in-reach weighting, then downstream node are most affected.

Due to permutation strategy, where the proportion of DEGs in pathways is approx. Same as in the dataset, pathways with few DEGs tend to have higher p-value regardless of their position on the topology.

TAPPA

TAPPA was introduced in [18]. The pathway-level statistic is called a Pathway Connectivity Index (PCI). The index is inspired by the second-order molecular connectivity index from chemoinformatics. Each sample expression values are first normalized to zero mean and same scope and then also by Sigmoid function to range $(-0.5, 0.5)$. The PCI is defined as

$$PCI = \sum_{i=1}^{N} \sum_{j=1}^{N} sgn(x_{is} + x_{js}) |x_{is}|^{0.5} a_{ij} |x_{js}|^{0.5},$$

where $a_{ij} = 1$ if there is an edge between genes i and j or $i = j$ and $a_{ij} = 0$ otherwise, x_{is} denotes the expression value of gene i in sample s and $sgn(x_{is} + x_{js})$ represents the overall expression status of the gene pair (up- or down-regulation). PCI normalized to the pathway size follow the normal distribution, therefore the Mann-Whitney test can be applied to test the hypothesis of equal medians of PCI in two groups. The pathway topology is incorporated as the higher contribution of the hub genes to PCI.

Non-topological variants of selected methods

For TopologyGSA and clipper, the IPS algorithm was not applied, and the sample covariance matrices were used as the final estimates. The DEGraph method provides two p-values for each connected component. One from Hotelling's test on full space (without topology) and one from Hotelling's test on reduced space (after reduction based on Fourier transformation). The Hottelings test on full space (without topology) was therefore used as a non-topological variant of the DEGraph. Similarly, SPIA method also returns two p-values (one from the hypergeometric test and one related to the perturbation factors), which are later combined via Fisher's method. Again, the hypergeometric test was used as a non-topological variant of the SPIA method. To avoid topology incorporation for PRS method, the weighting function was modified to set equal weights for all differentially expressed genes (multiplicative effect of DEG aggregation downstream from a gene was removed). The equal-weight centrality represents the non-topological variant of CePa method. This centrality is the same for all genes. To remove topological information in TAPPA method we removed all interactions between different genes (preserving only self-interactions).

Results

Tables 4 and 5 show results of the target pathway p-values and ranks in the Disease-Control Data Collection and Breast Cancer Data Collection.

Discussion

The methods' behavior was studied from several perspectives: the proportion of differentially expressed pathways under various conditions, the effect of pathway size and the effect of individual or multiple genes under different strategies for pre-processing of pathway topologies.

The ORA (univariable) methods are often criticized for arbitrary thresholds used to identify differentially expressed genes prior pathway analysis. However, in our experiments, we observed a stable proportion of DEPs in SPIA, PRS and CePa for three distinct thresholds. At certain method-specific sample size and threshold $p < 0.05$, the number of identified differentially expressed pathways decreased with increasing sample size.

We confirmed the expected type I error rate (5%) for all methods except CePa. The higher type I error rate observed in CePa method can be explained by its algorithm. The method calculates six independent centralities and returns a separate p-value associated with each centrality. The authors suggest to use the minimal p-value for the final decision on pathway significance, but the minimal p-values are no longer uniformly distributed (compared to individual centralities). Despite higher type I error rate, CePa does not detect many pathways as differentially expressed because of the competitive hypothesis: pathways which contain less differentially expressed genes than expected from the proportion of differentially expressed genes in the datasets are rarely detected as differentially expressed. Moreover, due to the ORA nature of CePa, a gene contributes to pathway-level statistic only when it is detected as differentially expressed, and all differentially expressed genes in the pathway contribute equally. When the fold-change of a gene is further increased, its effect on the pathway-level statistics stabilizes, as well as the resulting p-value.

When we artificially overexpressed single genes in simulated datasets, we could identify which specific gene had the strongest effect on the pathway significance. In all methods but TopologyGSA and Clipper, we could describe topological properties of these genes which can be to certain extent associated with their biological function. All tested genes had high or very high influence in TopologyGSA and Clipper. The most influential genes were those with the highest overall expression. Highly expressed genes are usually related to the cell cycle regulation [**?**]. Other methods showed various degrees of sensitivity to changes in individual genes within a single pathway. The topological properties of the most influential genes agreed with the methods algorithms. In DEGraph, SPIA, CePa and TAPPA, the influence of individual genes was related only to the number (or type) of incoming or outgoing interactions, regardless of the differential expression of the interacting genes. In DEGraph, the genes without incoming interactions had high influence correlating with their high algebraic connectivity (eigenvector related to the second-smallest eigenvalue of the Laplacian matrix of pathway graph). These genes are often represented either by ligands or receptors or transcription factors (E2F family dissociating from pRB).

		**SPIA**	**PRS**	**CePa**	**TAPPA**	**TopologyGSA**	**Clipper**	**DEGraph**
	+GPT	10/33	23/36	19/36	15/36	9/10	25/33	22/31
**$p \leq 0.05$**	-GPT	16/36	17/36	15/36	16/36	5/7	21/28	9/13
	MSPT	8/27	18/36	9/36	-	-	24/31	21/30
	+GPT	6/33	15/36	15/36	3/36	9/10	23/33	7/31
**top 10**	-GPT	10/36	15/36	10/36	3/36	5/7	19/28	4/13
	MSPT	5/27	9/36	3/36	-	-	21/31	6/30

Table 4: **P-values and ranks of the target pathways in Disease-Control Data Collection**
p-values and ranks of the target pathways in Disease-Control Data Collection for all compared methods and topologies. Ranks are p-value based. Pathway with the lowest p-value has rank 1. All pathways with the same p-value received the same rank. The rank was incremented by one between subsequent p-values.

		SPIA	PRS	CePa	TAPPA	TopologyGSA	Clipper	DEGraph
**Endocrine and other factor-regulated calcium reabsorption**								
	+GPT	4/27	3/27	2/27	21/27	26/26	27/27	23/27
**p ≤ 0.05**	-GPT	1/27	5/27	1/27	20/27	26/26	23/23	25/27
	MSPT	2/27	18/27	0/27	-	-	25/25	23/27
	+GPT	1/27	2/27	0/27	6/27	26/26	27/27	21/27
**top 10**	-GPT	0/27	2/27	0/27	5/27	25/26	21/23	20/27
	MSPT	2/27	12/27	0/27	-	-	25/25	20/27
**Estrogen signaling pathway**								
	+GPT	3/27	6/27	8/27	4/27	26/26	27/27	25/27
**p ≤ 0.05**	-GPT	6/27	14/27	3/27	6/27	19/22	17/20	22/24
	MSPT	4/27	16/27	1/27	-	-	25/25	26/27
	+GPT	2/27	3/27	4/27	0/27	26/26	27/27	15/27
**top 10**	-GPT	1/27	11/27	2/27	0/27	19/22	16/20	7/24
	MSPT	2/27	7/27	0/27	-	-	25/25	15/27
**Prolactin signaling pathway**								
	+GPT	1/27	3/27	3/27	8/27	26/26	27/27	25/27
**p ≤ 0.05**	-GPT	1/27	7/27	1/27	11/27	22/24	19/21	24/25
	MSPT	1/27	8/27	2/27	-	-	25/25	24/27
	+GPT	0/27	2/27	1/27	0/27	26/26	27/27	4/27
**top 10**	-GPT	0/27	4/27	0/27	0/27	21/24	19/21	6/25
	MSPT	1/27	5/27	1/27	-	-	25/25	5/27
**Thyroid hormone signaling pathway**								
	+GPT	1/27	1/27	6/27	6/27	19/19	26/26	26/27
**p ≤ 0.05**	-GPT	2/27	5/27	2/27	10/27	18/20	19/20	19/21
	MSPT	1/27	9/27	5/27	-	-	25/25	26/27
	+GPT	0/27	1/27	2/27	0/27	19/19	26/26	14/27
**top 10**	-GPT	0/27	2/27	0/27	1/27	17/20	19/20	5/21
	MSPT	0/27	6/27	3/27	-	-	25/25	7/27

Table 5: **P-values and ranks of the estrogen receptor containing pathways in Breast Cancer Data Collection**
p-values and ranks of the estrogen receptor containing pathways in Breast Cancer Data Collection for all compared methods and topologies. Ranks are p-value based. Pathway with the lowest p-value has rank 1. All pathways with the same p-value received the same rank. The rank was incremented by one between subsequent p-values.

In SPIA, the most influential genes had none or only neutral (e.g. binding) incoming interaction and could propagate their perturbation to many downstream genes. This is in accordance with the receptors of signaling pathways being the most important genes according to the authors. We also observed decreasing influence from ligands to receptors to secondary effectors. Due to the bidirectional interaction between EGFR and PLCG1 and PLCG2 in the Non-small cell lung cancer pathway, PLCG1 and PLCG2 had a higher impact than EGFR. If ligands are included in a pathway topology, receptors are not the most influential molecules. In CePa, we identified one gene with high influence in each model pathway. This gene was mapped to the node with the highest betweenness centrality, which is the centrality with the highest values amongst the six centralities suggested in the original CePa method. Secondary effectors tend to have highest betweenness centrality. Note, that in our analyses we used the smallest p-value from six distinct centralities as a representative value for a pathway. As stated in the original paper, each centrality is aimed at different genes. For example, transcription factors are typically the last effectors in a signaling pathway and as such have the highest in-reach centrality. Alternatively, the gene(s) which interact with members of a large gene family (pre-processed by `graphite`'s strategy) have a high out-degree centrality. To sum up, selection of a specific centrality can increase the specificity of analysis. In TAPPA, individual genes had very low to medium influence, and the hub genes had the highest influence within each tested pathway. Hub genes (genes with many incoming or outgoing interactions) are often effectors interacting with gene families or subunits of a large protein complex (in `graphite`'s preprocessing). In PRS method the influence of individual gene depends on the differential expression of the interacting genes not only on the number of interactions. Although we observed only low to medium gene influence across all simulated datasets, the overexpression of a gene interacting with other differentially expressed genes resulted in a decreased p-value in the individual datasets. These genes are frequently effector molecules in the middle of a signaling cascade.

The overexpression of a single gene had a different effect in the non-topological variants of the compared methods. Whereas for SPIA and CePa we observed a reduction of influence for all genes to 'very low', the influence of individual genes in PRS increased (although remained 'low'). Interestingly, in TAPPA, the combined gene influence in the topological variant is very similar to the non-topological one. However, the variability between individual genes was reduced. For DEGraph we observed a dramatic increase in gene effect (to 'high' and 'very high'). The individual genes had a similar influence on both topological and non-topological variants of TopologyGSA and Clipper.

Additionally, we observed no difference in Clipper performance on `graphite`'s and method-specific pathway topologies. Note that method-specific pre-processing of pathway topologies for Clipper matched the first version of `graphite` package as described in [62]. The DEGraph method was sensitive to the pre-processing of pathway topologies only for pathways that contained protein complexes since the pre-processing of these nodes perturbs the topology the most. The `graphite`'s strategy was beneficial for SPIA method in which it allowed deeper propagation of perturbation factors. On the other hand, the expansion of gene families and protein complexes into individual members or subunits reduced the influence of genes belonging to different gene groups, as detected for PRS in the Non-small cell lung cancer and also in CePa. Moreover, these expansions increased the

degree centralities significantly, further influencing the performance of CePa. Our results suggest that, for PRS and CePa, the method-specific pathway pre-processing seams to be more appropriate and should be preferred to `graphite's` approach. However, the pre-processing of gene families or protein complexes in MSPT of CePa and PRS, may not be biologically sustained and further research is needed to identify an optimal pre-processing strategy for these methods.

Analyses of real datasets

The observations on simulated datasets were confirmed by the analysis of real datasets from public data collections. In each dataset, we first identified pathways that we expected to be differentially expressed due to the experimental design (target pathways) and then compared the methods in terms of p-values assigned to these pathways. Whereas multivariable methods showed stable performance ($p < 0.05$ for most of the target pathways) over various real datasets, univariable methods differed in their ability to detect target pathways. However, in most datasets, multivariable methods identified over 50% of the pathways as differentially expressed (TopologyGSA and clipper over 90%) resulting in a much less informative outcome for the generation of hypotheses for further research. The CePa method outperformed all the other univariable methods in detecting the estrogen receptor-containing pathways as differentially expressed between estrogen receptor positive and estrogen receptor negative breast tumors. This observation suggests that the differentially expressed genes in these pathways occupy mostly nodes with high centralities than cluster together (PRS preference) or occupy root positions (SPIA preference).

However, when the goal was to identify differentially expressed pathways between patients and healthy controls, PRS was the best performing method suggesting that, in this situation, differentially expressed genes tend to make connected clusters rather than occupy important positions (either by centrality, or root nodes). This is a consequence of the (large) number of genes that are simultaneously deregulated in the diseased (tumor) samples and of the fact that they are involved in the target pathways.

The exclusion of topological information had a detrimental influence on p-values and ranks of estrogen receptor-containing pathways in CePa and also Endocrine and other factor-regulated calcium reabsorption in SPIA. However, the p-values and ranks were lower in PRS and DEGraph and further decreased in PRS for Breast Cancer Data Collection when method-specific pathway topologies were used. It implies that univariable methods benefit more from the inclusion of topological information than DEGraph and that inappropriate pre-processing can have a dramatic effect on the methods' performance. Interestingly, the exclusion of the topological information had the opposite effect on the univariable methods (lower p-values of target pathways in SPIA, but higher in PRS and CePa) in the Disease-Control Data Collection. It seems that higher proportion of the differentially expressed genes in the target pathways can surpass the topological effects of individual genes. After the exclusion of the topological information, the differences between TopologyGSA, Clipper and DEGraph vanished, and all the methods exhibited very high sensitivity to the expression change in a pathway with concordance to the *self-contained* null hypothesis of Hotelling's T^2 test.

Bibliography

[1] Sheela A. Abraham, Lisa EM Hopcroft, Emma Carrick, Mark E. Drotar, Karen Dunn, Andrew JK Williamson, Koorosh Korfi, Pablo Baquero, Laura E. Park, Mary T. Scott, Francesca Pellicano, Andrew Pierce, Mhairi Copland, Craig Nourse, Sean M. Grimmond, David Vetrie, Anthony D. Whetton, and Tessa L. Holyoake. Dual targeting of p53 and c-myc selectively eliminates leukaemic stem cells. *Nature*, 534(7607):341–346, Jun 2016. 27281222[pmid].

[2] M. Affer, S. Dao, C. Liu, A. B. Olshen, Q. Mo, A. Viale, C. L. Lambek, T. G. Marr, and B. D. Clarkson. Gene expression differences between enriched normal and chronic myelogenous leukemia quiescent stem/progenitor cells and correlations with biological abnormalities. *J Oncol*, 2011:798592, Feb 2011. 21436996[pmid].

[3] Maysson Al-Haj Ibrahim, Sabah Jassim, Michael Anthony Cawthorne, and Kenneth Langlands. A topology-based score for pathway enrichment. *J Comput Biol*, 2012.

[4] T. W. Anderson and Theodore W. Anderson. *An Introduction to Multivariate Statistical Analysis, 2nd Edition*. Wiley, 2 edition, September 1984.

[5] Liviu Badea, Vlad Herlea, Simona Olimpia Dima, Traian Dumitrascu, and Popescu Irinel. Combined gene expression analysis of whole-tissue and microdissected pancreatic ductal adenocarcinoma identifies genes specifically overexpressed in tumor epithelia. *Hepato-Gastroenterology*, 88:2016–27, 2008.

[6] Andreas S. Barth, Ruprecht Kuner, Andreas Buness, Markus Ruschhaupt, Sylvia Merk, Ludwig Zwermann, Stefan Kb, Eckart Kreuzer, Gerhard Steinbeck, Ulrich Mansmann, Annemarie Poustka, Michael Nabauer, and Holger Sltmann. Identification of a common gene expression signature in dilated cardiomyopathy across independent microarray studies. *Journal of the American College of Cardiology*, 48(8):1610 – 1617, 2006.

[7] Gaurav Bhatti. *KEGGandMetacoreDzPathwaysGEO: Disease Datasets from GEO*, 2014. R package version 0.106.0.

[8] Gaurav Bhatti and Adi L. Tarca. *KEGGdzPathwaysGEO: KEGG Disease Datasets from GEO*, 2012. R package version 1.10.0.

[9] Andrea H. Bild, Guang Yao, Jeffrey T. Chang, Quanli Wang, Anil Potti, Dawn Chasse, Mary-Beth Joshi, David Harpole, Johnathan M. Lancaster,

Andrew Berchuck, John A. Olson, Jeffrey R. Marks, Holly K. Dressman, Mike West, and Joseph R. Nevins. Oncogenic pathway signatures in human cancers as a guide to targeted therapies. *Nature*, 439(7074):353–7, January 2006.

[10] Eric M. Blalock, James W. Geddes, Kuey Chu Chen, Nada M. Porter, William R. Markesbery, and Philip W. Landfield. Incipient alzheimer's disease: Microarray correlation analyses reveal major transcriptional and tumor suppressor responses. *Proceedings of the National Academy of Sciences*, 101(7):2173–2178, 2004.

[11] Koei Chin, Sandy DeVries, Jane Fridlyand, Paul T. Spellman, Ritu Roydasgupta, Wen-Lin Kuo, Anna Lapuk, Richard M. Neve, Zuwei Qian, Tom Ryder, Fanqing Chen, Heidi Feiler, Taku Tokuyasu, Chris Kingsley, Shanaz Dairkee, Zhenhang Meng, Karen Chew, Daniel Pinkel, Ajay Jain, Britt M. Ljung, Laura Esserman, Donna G. Albertson, Frederic M. Waldman, and Joe W. Gray. Genomic and transcriptional aberrations linked to breast cancer pathophysiologies. *Cancer Cell*, 10(6):529–541, December 2006.

[12] Sarah Dedeurwaerder, Christine Desmedt, Emilie Calonne, Sandeep K. Singhal, Benjamin Haibe-Kains, Matthieu Defrance, Stefan Michiels, Michael Volkmar, Rachel Deplus, Judith Luciani, Françoise Lallemand, Denis Larsimont, Jérôme Toussaint, Sandy Haussy, Françoise Rothé, Ghizlane Rouas, Otto Metzger, Samira Majjaj, Kamal Saini, Pascale Putmans, Gérald Hames, Nicolas van Baren, Pierre G. Coulie, Martine Piccart, Christos Sotiriou, and François Fuks. Dna methylation profiling reveals a predominant immune component in breast cancers. *EMBO Molecular Medicine*, 3(12):726–741, 2011.

[13] Christine Desmedt, Fanny Piette, Sherene Loi, Yixin Wang, Françoise Lallemand, Benjamin Haibe-Kains, Giuseppe Viale, Mauro Delorenzi, Yi Zhang, Mahasti Saghatchian d'Assignies, Jonas Bergh, Rosette Lidereau, Paul Ellis, Adrian L. Harris, Jan G.M. Klijn, John A. Foekens, Fatima Cardoso, Martine J. Piccart, Marc Buyse, and Christos Sotiriou. Strong time dependence of the 76-gene prognostic signature for node-negative breast cancer patients in the transbig multicenter independent validation series. *Clinical Cancer Research*, 13(11):3207–3214, 2007.

[14] Timothy R. Donahue, Linh M. Tran, Reginald Hill, Yunfeng Li, Anne Kovochich, Joseph H. Calvopina, Sanjeet G. Patel, Nanping Wu, Antreas Hindoyan, James J. Farrell, Xinmin Li, David W. Dawson, and Hong Wu. Integrative survival-based molecular profiling of human pancreatic cancer. *Clinical Cancer Research*, 18(5):1352–1363, 2012.

[15] Sorin Draghici, Purvesh Khatri, Adi Laurentiu Tarca, Kashyap Amin, Arina Done, Calin Voichita, Constantin Georgescu, and Roberto Romero. A systems biology approach for pathway level analysis. *Genome Research*, 17(10):000, 2007.

[16] Pierre Farmer, Herve Bonnefoi, Veronique Becette, Michele Tubiana-Hulin, Pierre Fumoleau, Denis Larsimont, Gaetan Macgrogan, Jonas Bergh, David Cameron, Darlene Goldstein, Stephan Duss, Anne-Laure Nicoulaz, Cathrin

Brisken, Maryse Fiche, Mauro Delorenzi, and Richard Iggo. Identification of molecular apocrine breast tumours by microarray analysis. *Oncogene*, 24(29):4660–71, 7 2005.

[17] Orsolya Galamb, Balzs Gyrffy, Ferenc Sipos, Sndor Spisk, Anna Mria Nmeth, Pl Miheller, Zsolt Tulassay, Elek Dinya, and Bla Molnr. Inflammation, adenoma and cancer: Objective classification of colon biopsy specimens with gene expression signature. *Disease markers*, 25(1):1–16, 2008.

[18] Shouguo Gao and Xujing Wang. Tappa: topological analysis of pathway phenotype association. *Bioinformatics*, 23(22):3100–3102, 2007.

[19] Jelle J. Goeman and Peter Bhlmann. Analyzing gene expression data in terms of gene sets: methodological issues. *Bioinformatics*, 23(8):980–987, 2007.

[20] Zuguang Gu, Jialin Liu, Kunming Cao, Junfeng Zhang, and Jin Wang. Centrality-based pathway enrichment: a systematic approach for finding significant pathways dominated by key genes. *BMC Systems Biology*, 6(1):56, 2012.

[21] Balazs Gyorffy, Bela Molnar, Hermann Lage, Zoltan Szallasi, and Aron C. Eklund. Evaluation of microarray preprocessing algorithms based on concordance with rt-pcr in clinical samples. *PLOS ONE*, 4(5):1–6, 05 2009.

[22] Benjamin Haibe-Kains, Christine Desmedt, Sherene Loi, Aedin C. Culhane, Gianluca Bontempi, John Quackenbush, and Christos Sotiriou. A three-gene model to robustly identify breast cancer molecular subtypes. *Journal of the National Cancer Institute*, 104(4):311–325, February 2012.

[23] Huiling He, Krystian Jazdzewski, Wei Li, Sandya Liyanarachchi, Rebecca Nagy, Stefano Volinia, George A. Calin, Chang-gong Liu, Kaarle Franssila, Saul Suster, Richard T. Kloos, Carlo M. Croce, and Albert de la Chapelle. The role of microrna genes in papillary thyroid carcinoma. *Proceedings of the National Academy of Sciences of the United States of America*, 102(52):19075–19080, 2005.

[24] Kenneth R. Hess, Keith Anderson, W. Fraser Symmans, Vicente Valero, Nuhad Ibrahim, Jaime A. Mejia, Daniel Booser, Richard L. Theriault, Aman U. Buzdar, Peter J. Dempsey, Roman Rouzier, Nour Sneige, Jeffrey S. Ross, Tatiana Vidaurre, Henry L. Gmez, Gabriel N. Hortobagyi, and Lajos Pusztai. Pharmacogenomic predictor of sensitivity to preoperative chemotherapy with paclitaxel and fluorouracil, doxorubicin, and cyclophosphamide in breast cancer. *Journal of Clinical Oncology*, 24(26):4236–4244, 2006. PMID: 16896004.

[25] Aniko Hever, Richard B. Roth, Peter Hevezi, Maria E. Marin, Jose A. Acosta, Hector Acosta, Jose Rojas, Rosa Herrera, Dimitri Grigoriadis, Evan White, Paul J. Conlon, Richard A. Maki, and Albert Zlotnik. Human endometriosis is associated with plasma cells and overexpression of b lymphocyte stimulator. *Proceedings of the National Academy of Sciences*, 104(30):12451–12456, 2007.

[26] Yi Hong, Thomas Downey, Kong Weng Eu, Poh Koon Koh, and Peh Yean Cheah. A 'metastasis-prone' signature for early-stage mismatch-repair proficient sporadic colorectal cancer patients and its implications for possible therapeutics. *Clinical & Experimental Metastasis*, 27(2):83–90, 2010.

[27] Yi Hong, Kok Sun Ho, Kong Weng Eu, and Peh Yean Cheah. A susceptibility gene set for early onset colorectal cancer that integrates diverse signaling pathways: Implication for tumorigenesis. *Clinical Cancer Research*, 13(4):1107–1114, 2007.

[28] Jun Hou, Joachim Aerts, Bianca den Hamer, Wilfred van IJcken, Michael den Bakker, Peter Riegman, Cor van der Leest, Peter van der Spek, John A. Foekens, Henk C. Hoogsteden, Frank Grosveld, and Sjaak Philipsen. Gene expression-based classification of non-small cell lung carcinomas and survival prediction. *PLOS ONE*, 5(4):1–12, 04 2010.

[29] Erich Huang, Skye H Cheng, Holly Dressman, Jennifer Pittman, Mei Hua Tsou, Cheng Fang Horng, Andrea Bild, Edwin S Iversen, Ming Liao, Chii Ming Chen, Mike West, Joseph R Nevins, and Andrew T Huang. Gene expression predictors of breast cancer outcomes. *Lancet (London, England)*, 361(9369):15901596, May 2003.

[30] L. Jacob, P. Neuvial, and S. Dudoit. Gains in Power from Structured Two-Sample Tests of Means on Graphs. *ArXiv e-prints*, September 2010.

[31] Nicolai Juul, Zoltan Szallasi, Aron C. Eklund, Qiyuan Li, Rebecca A. Burrell, Marco Gerlinger, Vicente Valero, Eleni Andreopoulou, Francisco J. Esteva, and W. Fraser Symmans. Assessment of an RNA interference screen-derived mitotic and ceramide pathway metagene as a predictor of response to neoadjuvant paclitaxel for primary triple-negative breast cancer: a retrospective analysis of five clinical trials. *The Lancet Oncology*, 11(4):358–365, April 2010.

[32] Purvesh Khatri, Sorin Draghici, Adi L. Tarca, Sonia S. Hassan, and Roberto Romero. A system biology approach for the steady-state analysis of gene signaling networks. In *Proceedings of the Congress on pattern recognition 12th Iberoamerican conference on Progress in pattern recognition, image analysis and applications*, CIARP'07, pages 32–41, Berlin, Heidelberg, 2007. Springer-Verlag.

[33] Purvesh Khatri, Marina Sirota, and Atul J. Butte. Ten years of pathway analysis: Current approaches and outstanding challenges. *PLoS Comput Biol*, 8(2):e1002375, 02 2012.

[34] Jong Wook Kim, Seiichi Mori, and Joseph R. Nevins. Myc-induced micrornas integrate myc-mediated cell proliferation and cell fate. *Cancer Research*, 70(12):4820–4828, 2010.

[35] Steffen L. Lauritzen. *Graphical Models*. Oxford Statistical Science Series. Oxford University Press, New York, USA, July 1996.

[36] Rifca Le Dieu, David C. Taussig, Alan G. Ramsay, Richard Mitter, Faridah Miraki-Moud, Rewas Fatah, Abigail M. Lee, T. Andrew Lister, and John G.

Gribben. Peripheral blood t cells in acute myeloid leukemia (aml) patients at diagnosis have abnormal phenotype and genotype and form defective immune synapses with aml blasts. *Blood*, 114(18):3909–3916, 2009.

[37] Marc E. Lenburg, Louis S. Liou, Norman P. Gerry, Garrett M. Frampton, Herbert T. Cohen, and Michael F. Christman. Previously unidentified changes in renal cell carcinoma gene expression identified by parametric analysis of microarray data. *BMC Cancer*, 3(1):31, 2003.

[38] Yang Li, Lihua Zou, Qiyuan Li, Benjamin Haibe-Kains, Ruiyang Tian, Yan Li, Christine Desmedt, Christos Sotiriou, Zoltan Szallasi, J. Dirk Iglehart, Andrea L. Richardson, and Zhigang C. Wang. Amplification of LAPTM4B and YWHAZ contributes to chemotherapy resistance and recurrence of breast cancer. *Nature Medicine*, 16(2):214–218, January 2010.

[39] Winnie S. Liang, Travis Dunckley, Thomas G. Beach, Andrew Grover, Diego Mastroeni, Douglas G. Walker, Richard J. Caselli, Walter A. Kukull, Daniel McKeel, John C. Morris, Christine Hulette, Donald Schmechel, Gene E. Alexander, Eric M. Reiman, Joseph Rogers, and Dietrich A. Stephan. Gene expression profiles in anatomically and functionally distinct regions of the normal aged human brain. *Physiological Genomics*, 28(3):311–322, 2007.

[40] Winnie S. Liang, Eric M. Reiman, Jon Valla, Travis Dunckley, Thomas G. Beach, Andrew Grover, Tracey L. Niedzielko, Lonnie E. Schneider, Diego Mastroeni, Richard Caselli, Walter Kukull, John C. Morris, Christine M. Hulette, Donald Schmechel, Joseph Rogers, and Dietrich A. Stephan. Alzheimer's disease is associated with reduced expression of energy metabolism genes in posterior cingulate neurons. *Proceedings of the National Academy of Sciences*, 105(11):4441–4446, 2008.

[41] Cornelia Liedtke, Chafika Mazouni, Kenneth R. Hess, Fabrice Andr, Attila Tordai, Jaime A. Mejia, W. Fraser Symmans, Ana M. Gonzalez-Angulo, Bryan Hennessy, Marjorie Green, Massimo Cristofanilli, Gabriel N. Hortobagyi, and Lajos Pusztai. Response to neoadjuvant therapy and long-term survival in patients with triple-negative breast cancer. *Journal of Clinical Oncology*, 26(8):1275–1281, 2008. PMID: 18250347.

[42] Zhongyu Liu, Zhiqiang Yao, Chao Li, Yicheng Lu, and Chunfang Gao. Gene expression profiling in human high-grade astrocytomas. *Comp Funct Genomics*, 2011:245137, Aug 2011. 21836821[pmid].

[43] Zhongyu Liu, Zhiqiang Yao, Chao Li, Yicheng Lu, and Chunfang Gao. Gene expression profiling in human high-grade astrocytomas. *Comp Funct Genomics*, 2011:245137, Aug 2011. 21836821[pmid].

[44] Sherene Loi, Benjamin Haibe-Kains, Christine Desmedt, Franoise Lallemand, Andrew M. Tutt, Cheryl Gillet, Paul Ellis, Adrian Harris, Jonas Bergh, John A. Foekens, Jan G.M. Klijn, Denis Larsimont, Marc Buyse, Gianluca Bontempi, Mauro Delorenzi, Martine J. Piccart, and Christos Sotiriou. Definition of clinically distinct molecular subtypes in estrogen receptorpositive breast carcinomas through genomic grade. *Journal of Clinical Oncology*, 25(10):1239–1246, 2007. PMID: 17401012.

[45] Sherene Loi, Benjamin Haibe-Kains, Christine Desmedt, Pratyaksha Wirapati, Françoise Lallemand, Andrew M. Tutt, Cheryl Gillet, Paul Ellis, Kenneth Ryder, James F. Reid, Maria G. Daidone, Marco A. Pierotti, Els MJJ Berns, Maurice PHM Jansen, John A. Foekens, Mauro Delorenzi, Gianluca Bontempi, Martine J. Piccart, and Christos Sotiriou. Predicting prognosis using molecular profiling in estrogen receptor-positive breast cancer treated with tamoxifen. *BMC Genomics*, 9(1):239, 2008.

[46] Sherene Loi, Benjamin Haibe-Kains, Samira Majjaj, Francoise Lallemand, Virginie Durbecq, Denis Larsimont, Ana M. Gonzalez-Angulo, Lajos Pusztai, W. Fraser Symmans, Alberto Bardelli, Paul Ellis, Andrew N. J. Tutt, Cheryl E. Gillett, Bryan T. Hennessy, Gordon B. Mills, Wayne A. Phillips, Martine J. Piccart, Terence P. Speed, Grant A. McArthur, and Christos Sotiriou. Pik3ca mutations associated with gene signature of low mtorc1 signaling and better outcomes in estrogen receptorpositive breast cancer. *Proceedings of the National Academy of Sciences*, 107(22):10208–10213, 2010.

[47] Xuesong Lu, Xin Lu, Zhigang C. Wang, J. Dirk Iglehart, Xuegong Zhang, and Andrea L. Richardson. Predicting features of breast cancer with gene expression patterns. *Breast Cancer Research and Treatment*, 108(2):191, 2007.

[48] Paolo Martini, Gabriele Sales, M. Sofia Massa, Monica Chiogna, and Chiara Romualdi. Along signal paths: an empirical gene set approach exploiting pathway topology. *Nucleic Acids Research*, 2012.

[49] Maria Massa, Monica Chiogna, and Chiara Romualdi. Gene set analysis exploiting the topology of a pathway. *BMC Systems Biology*, 4(1):121, 2010.

[50] Lance D. Miller, Johanna Smeds, Joshy George, Vinsensius B. Vega, Liza Vergara, Alexander Ploner, Yudi Pawitan, Per Hall, Sigrid Klaar, Edison T. Liu, and Jonas Bergh. An expression signature for p53 status in human breast cancer predicts mutation status, transcriptional effects, and patient survival. *Proceedings of the National Academy of Sciences of the United States of America*, 102(38):13550–13555, 2005.

[51] Andy J. Minn, Gaorav P. Gupta, David Padua, Paula Bos, Don X. Nguyen, Dimitry Nuyten, Bas Kreike, Yi Zhang, Yixin Wang, Hemant Ishwaran, John A. Foekens, Marc van de Vijver, and Joan Massagu. Lung metastasis genes couple breast tumor size and metastatic spread. *Proceedings of the National Academy of Sciences*, 104(16):6740–6745, 2007.

[52] Andy J. Minn, Gaorav P. Gupta, Peter M. Siegel, Paula D. Bos, Weiping Shu, Dilip D. Giri, Agnes Viale, Adam B. Olshen, William L. Gerald, and Joan Massague. Genes that mediate breast cancer metastasis to lung. *Nature*, 436(7050):518–524, July 2005.

[53] Rachael Natrajan, Britta Weigelt, Alan Mackay, Felipe C. Geyer, Anita Grigoriadis, David S. P. Tan, Chris Jones, Christopher J. Lord, Radost Vatcheva, Socorro M. Rodriguez-Pinilla, Jose Palacios, Alan Ashworth, and Jorge S. Reis-Filho. An integrative genomic and transcriptomic analysis reveals molecular pathways and networks regulated by copy number

aberrations in basal-like, her2 and luminal cancers. *Breast Cancer Research and Treatment*, 121(3):575–589, 2010.

[54] Juan Nunez-Iglesias, Chun-Chi Liu, Todd E. Morgan, Caleb E. Finch, and Xianghong Jasmine Zhou. Joint genome-wide profiling of mirna and mrna expression in alzheimer's disease cortex reveals altered mirna regulation. *PLOS ONE*, 5(2):1–9, 02 2010.

[55] Yudi Pawitan, Judith Bjöhle, Lukas Amler, Anna-Lena Borg, Suzanne Egyhazi, Per Hall, Xia Han, Lars Holmberg, Fei Huang, Sigrid Klaar, Edison T. Liu, Lance Miller, Hans Nordgren, Alexander Ploner, Kerstin Sandelin, Peter M. Shaw, Johanna Smeds, Lambert Skoog, Sara Wedrén, and Jonas Bergh. Gene expression profiling spares early breast cancer patients from adjuvant therapy: derived and validated in two population-based cohorts. *Breast Cancer Research*, 7(6):R953, 2005.

[56] Huadong Pei, Liang Li, Brooke L. Fridley, Gregory D. Jenkins, Krishna R. Kalari, Wilma Lingle, Gloria Petersen, Zhenkun Lou, and Liewei Wang. {FKBP51} affects cancer cell response to chemotherapy by negatively regulating akt. *Cancer Cell*, 16(3):259 – 266, 2009.

[57] Vlad Popovici, Weijie Chen, Brandon D. Gallas, Christos Hatzis, Weiwei Shi, Frank W. Samuelson, Yuri Nikolsky, Marina Tsyganova, Alex Ishkin, Tatiana Nikolskaya, Kenneth R. Hess, Vicente Valero, Daniel Booser, Mauro Delorenzi, Gabriel N. Hortobagyi, Leming Shi, W. Fraser Symmans, and Lajos Pusztai. Effect of training-sample size and classification difficulty on the accuracy of genomic predictors. *Breast Cancer Research*, 12(1):R5, 2010.

[58] Aleix Prat, Joel S. Parker, Olga Karginova, Cheng Fan, Chad Livasy, Jason I. Herschkowitz, Xiaping He, and Charles M. Perou. Phenotypic and molecular characterization of the claudin-low intrinsic subtype of breast cancer. *Breast Cancer Research*, 12(5):R68, 2010.

[59] Heike Runne, Alexandre Kuhn, Edward J. Wild, Wirahpati Pratyaksha, Mark Kristiansen, Jeremy D. Isaacs, Etienne Rgulier, Mauro Delorenzi, Sarah J. Tabrizi, and Ruth Luthi-Carter. Analysis of potential transcriptomic biomarkers for huntington's disease in peripheral blood. *Proceedings of the National Academy of Sciences*, 104(36):14424–14429, 2007.

[60] Lao H. Saal, Peter Johansson, Karolina Holm, Sofia K. Gruvberger-Saal, Qing-Bai She, Matthew Maurer, Susan Koujak, Adolfo A. Ferrando, Per Malmstrm, Lorenzo Memeo, Jorma Isola, Pr-Ola Bendahl, Neal Rosen, Hanina Hibshoosh, Markus Ringnr, ke Borg, and Ramon Parsons. Poor prognosis in carcinoma is associated with a gene expression signature of aberrant pten tumor suppressor pathway activity. *Proceedings of the National Academy of Sciences*, 104(18):7564–7569, 2007.

[61] Jacob Sabates-Bellver, Laurens G. Van der Flier, Mariagrazia de Palo, Elisa Cattaneo, Caroline Maake, Hubert Rehrauer, Endre Laczko, Michal A. Kurowski, Janusz M. Bujnicki, Mirco Menigatti, Judith Luz, Teresa V. Ranalli, Vito Gomes, Alfredo Pastorelli, Roberto Faggiani, Marcello Anti, Josef Jiricny, Hans Clevers, and Giancarlo Marra. Transcriptome profile of

human colorectal adenomas. *Molecular Cancer Research*, 5(12):1263–1275, 2008.

[62] Gabriele Sales, Enrica Calura, Duccio Cavalieri, and Chiara Romualdi. graphite - a bioconductor package to convert pathway topology to gene network. *BMC Bioinformatics*, 13(1):20, 2012.

[63] Abel Sanchez-Palencia, Mercedes Gomez-Morales, Jose Antonio Gomez-Capilla, Vicente Pedraza, Laura Boyero, Rafael Rosell, and M Esther Frez-Vidal. Gene expression profiling reveals novel biomarkers in nonsmall cell lung cancer. *International Journal of Cancer*, 129(2):355–364, 2011.

[64] Juliane Schäfer and Korbinian Strimmer. A Shrinkage Approach to Large-Scale Covariance Matrix Estimation and Implications for Functional Genomics. *Statistical Applications in Genetics and Molecular Biology*, 4(1), January 2005.

[65] Marcus Schmidt, Daniel Böhm, Christian von Törne, Eric Steiner, Alexander Puhl, Henryk Pilch, Hans-Anton Lehr, Jan G. Hengstler, Heinz Kölbl, and Mathias Gehrmann. The humoral immune system has a key prognostic impact in node-negative breast cancer. *Cancer Research*, 68(13):5405–5413, 2008.

[66] Daniel P. Silver, Andrea L. Richardson, Aron C. Eklund, Zhigang C. Wang, Zoltan Szallasi, Qiyuan Li, Nicolai Juul, Chee-Onn Leong, Diana Calogrias, Ayodele Buraimoh, Aquila Fatima, Rebecca S. Gelman, Paula D. Ryan, Nadine M. Tung, Arcangela De Nicolo, Shridar Ganesan, Alexander Miron, Christian Colin, Dennis C. Sgroi, Leif W. Ellisen, Eric P. Winer, and Judy E. Garber. Efficacy of neoadjuvant cisplatin in triple-negative breast cancer. *Journal of Clinical Oncology*, 28(7):1145–1153, 2010. PMID: 20100965.

[67] Gordon K. Smyth. Linear models and empirical bayes methods for assessing differential expression in microarray experiments. *Statistical applications in genetics and molecular biology*, 3(1), January 2004.

[68] Christos Sotiriou, Soek-Ying Neo, Lisa M. McShane, Edward L. Korn, Philip M. Long, Amir Jazaeri, Philippe Martiat, Steve B. Fox, Adrian L. Harris, and Edison T. Liu. Breast cancer classification and prognosis based on gene expression profiles from a population-based study. *Proceedings of the National Academy of Sciences*, 100(18):10393–10398, September 2003.

[69] Christos Sotiriou, Pratyaksha Wirapati, Sherene Loi, Adrian Harris, Steve Fox, Johanna Smeds, Hans Nordgren, Pierre Farmer, Viviane Praz, Benjamin Haibe-Kains, Christine Desmedt, Denis Larsimont, Fatima Cardoso, Hans Peterse, Dimitry Nuyten, Marc Buyse, Marc J. Van de Vijver, Jonas Bergh, Martine Piccart, and Mauro Delorenzi. Gene expression profiling in breast cancer: Understanding the molecular basis of histologic grade to improve prognosis. *Journal of the National Cancer Institute*, 98(4):262–272, 2006.

[70] Therese S?rlie, Robert Tibshirani, Joel Parker, Trevor Hastie, J. S. Marron, Andrew Nobel, Shibing Deng, Hilde Johnsen, Robert Pesich, Stephanie

Geisler, Janos Demeter, Charles M. Perou, Per E. L?nning, Patrick O. Brown, Anne-Lise B?rresen-Dale, and David Botstein. Repeated observation of breast tumor subtypes in independent gene expression data sets. *Proceedings of the National Academy of Sciences*, 100(14):8418–8423, 2003.

[71] Derek L. Stirewalt, Soheil Meshinchi, Kenneth J. Kopecky, Wenhong Fan, Era L. Pogosova-Agadjanyan, Julia H. Engel, Michelle R. Cronk, Kathleen Shannon Dorcy, Amy R. McQuary, David Hockenbery, Brent Wood, Shelly Heimfeld, and Jerald P. Radich. Identification of genes with abnormal expression changes in acute myeloid leukemia. *Genes, Chromosomes and Cancer*, 47(1):8–20, 2008.

[72] Aravind Subramanian, Pablo Tamayo, Vamsi K. Mootha, Sayan Mukherjee, Benjamin L. Ebert, Michael A. Gillette, Amanda Paulovich, Scott L. Pomeroy, Todd R. Golub, Eric S. Lander, and Jill P. Mesirov. Gene set enrichment analysis: A knowledge-based approach for interpreting genome-wide expression profiles. *Proceedings of the National Academy of Sciences of the United States of America*, 102(43):15545–15550, 2005.

[73] W. Fraser Symmans, Christos Hatzis, Christos Sotiriou, Fabrice Andre, Florentia Peintinger, Peter Regitnig, Guenter Daxenbichler, Christine Desmedt, Julien Domont, Christian Marth, Suzette Delaloge, Thomas Bauernhofer, Vicente Valero, Daniel J. Booser, Gabriel N. Hortobagyi, and Lajos Pusztai. Genomic index of sensitivity to endocrine therapy for breast cancer. *Journal of Clinical Oncology*, 28(27):4111–4119, 2010. PMID: 20697068.

[74] Yoshiaki Tabuchi, Ichiro Takasaki, Atsumi Suto, Takashi Kondo, Yoshihisa Suzuki, and Masuo Obinata. Genetic networks in nonpermissive temperature-induced cell differentiation of sertoli tte3 cells harboring temperature-sensitive sv40 large t-antigen. *Cell Biology International*, 31(10):1231–1236, 2007.

[75] Adi Laurentiu Tarca, Sorin Draghici, Purvesh Khatri, Sonia S. Hassan, Pooja Mittal, Jung-sun Kim, Chong Jai Kim, Juan Pedro Kusanovic, and Roberto Romero. A novel signaling pathway impact analysis. *Bioinformatics*, 25(1):75–82, 2009.

[76] Shailesh Tripathi and Frank Emmert-Streib. Assessment method for a power analysis to identify differentially expressed pathways. *PLOS ONE*, 7(5):1–13, 05 2012.

[77] Shahab Uddin, Maqbool Ahmed, Azhar Hussain, Jehad Abubaker, Nasser Al-Sanea, Alaa AbdulJabbar, Luai H. Ashari, Samar Alhomoud, Fouad Al-Dayel, Zeenath Jehan, Prashant Bavi, Abdul K. Siraj, and Khawla S. Al-Kuraya. Genome-wide expression analysis of middle eastern colorectal cancer reveals foxm1 as a novel target for cancer therapy. *Am J Pathol*, 178(2):537–547, Feb 2011. AJPA112[PII].

[78] F. H. J. van Tienen, S. F. E. Praet, H. M. de Feyter, N. M. van den Broek, P. J. Lindsey, K. G. C. Schoonderwoerd, I. F. M. de Coo, K. Nicolay, J. J. Prompers, H. J. M. Smeets, and L. J. C. van Loon. Physical activity is the key determinant of skeletal muscle mitochondrial function in type 2

diabetes. *The Journal of Clinical Endocrinology & Metabolism*, 97(9):3261–3269, 2012. PMID: 22802091.

[79] Tiffany A. Wallace, Robyn L. Prueitt, Ming Yi, Tiffany M. Howe, John W. Gillespie, Harris G. Yfantis, Robert M. Stephens, Neil E. Caporaso, Christopher A. Loffredo, and Stefan Ambs. Tumor immunobiological differences in prostate cancer between african-american and european-american men. *Cancer Research*, 68(3):927–936, 2008.

[80] Yi Wang, Olga Roche, Mathew S. Yan, Greg Finak, Andrew J. Evans, Julie L. Metcalf, Bridgid E. Hast, Sara C. Hanna, Bill Wondergem, Kyle A. Furge, Meredith S. Irwin, William Y. Kim, Bin T. Teh, Sergio Grinstein, Morag Park, Philip A. Marsden, and Michael Ohh. Regulation of endocytosis via the oxygen-sensing pathway. *Nat Med*, 15(3):319–324, March 2009.

[81] Yixin Wang, Jan G. Klijn, Yi Zhang, Anieta M. Sieuwerts, Maxime P. Look, Fei Yang, Dmitri Talantov, Mieke Timmermans, Marion E. Meijer-van Gelder, Jack Yu, Tim Jatkoe, Els M. Berns, David Atkins, and John A. Foekens. Gene-expression profiles to predict distant metastasis of lymph-node-negative primary breast cancer. *Lancet*, 365(9460):671–679, February 2005.

[82] Yanli Zhang, Michael James, Frank A. Middleton, and Richard L. Davis. Transcriptional analysis of multiple brain regions in parkinson's disease supports the involvement of specific protein processing, energy metabolism, and signaling pathways, and suggests novel disease mechanisms. *American Journal of Medical Genetics Part B: Neuropsychiatric Genetics*, 137B(1):5–16, 2005.

[83] Yi Zhang, Anieta M. Sieuwerts, Michelle McGreevy, Graham Casey, Tanja Cufer, Angelo Paradiso, Nadia Harbeck, Paul N. Span, David G. Hicks, Joseph Crowe, Raymond R. Tubbs, G. Thomas Budd, Joanne Lyons, Fred C. G. J. Sweep, Manfred Schmitt, Francesco Schittulli, Rastko Golouh, Dmitri Talantov, Yixin Wang, and John A. Foekens. The 76-gene signature defines high-risk patients that benefit from adjuvant tamoxifen therapy. *Breast Cancer Research and Treatment*, 116(2):303–309, 2009.

[84] Bin Zheng, Zhixiang Liao, Joseph J. Locascio, Kristen A. Lesniak, Sarah S. Roderick, Marla L. Watt, Aron C. Eklund, Yanli Zhang-James, Peter D. Kim, Michael A. Hauser, Edna Grünblatt, Linda B. Moran, Silvia A. Mandel, Peter Riederer, Renee M. Miller, Howard J. Federoff, Ullrich Wüllner, Spyridon Papapetropoulos, Moussa B. Youdim, Ippolita Cantuti-Castelvetri, Anne B. Young, Jeffery M. Vance, Richard L. Davis, John C. Hedreen, Charles H. Adler, Thomas G. Beach, Manuel B. Graeber, Frank A. Middleton, Jean-Christophe Rochet, Clemens R. Scherzer, and . Pgc-1, a potential therapeutic target for early intervention in parkinson's disease. *Science Translational Medicine*, 2(52):52ra73–52ra73, 2010.
