# Supplementary figures and images for: A critical comparison of topology-based pathway analysis methods
Source: PLoS One. 2018 Jan 25;13(1):e0191154. doi: 10.1371/journal.pone.0191154 (PMC5784953; doi:10.1371/journal.pone.0191154)

# Effect of the number of Entrez IDs

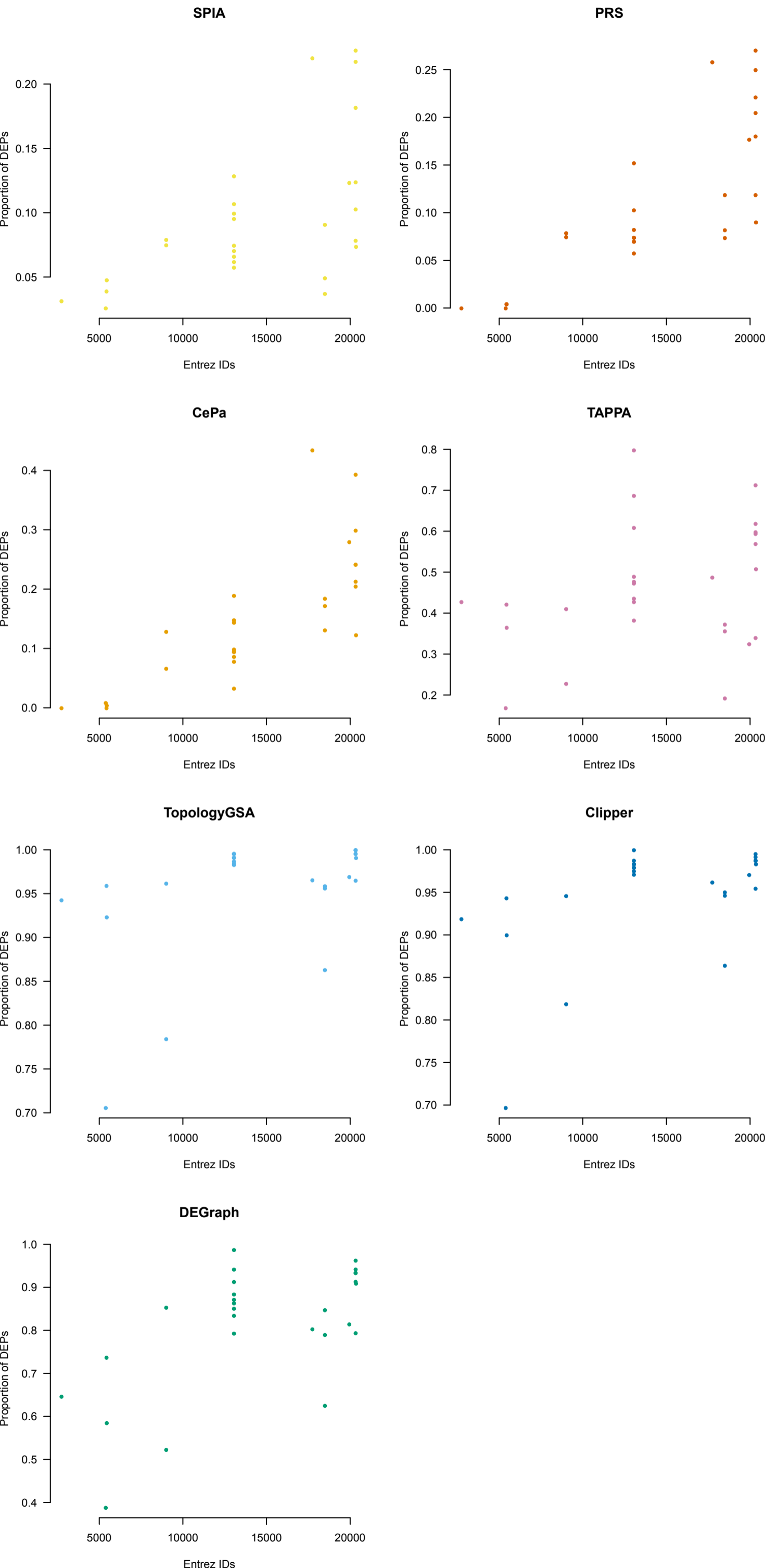

Supplement: S1 Fig — Proportion of DEPs depending on the number of Entrez IDs for datasets from Breast Cancer Data Collection. Each point represents one dataset. (PDF) [file pone.0191154.s002.pdf]

# Effect of the number of DEGs

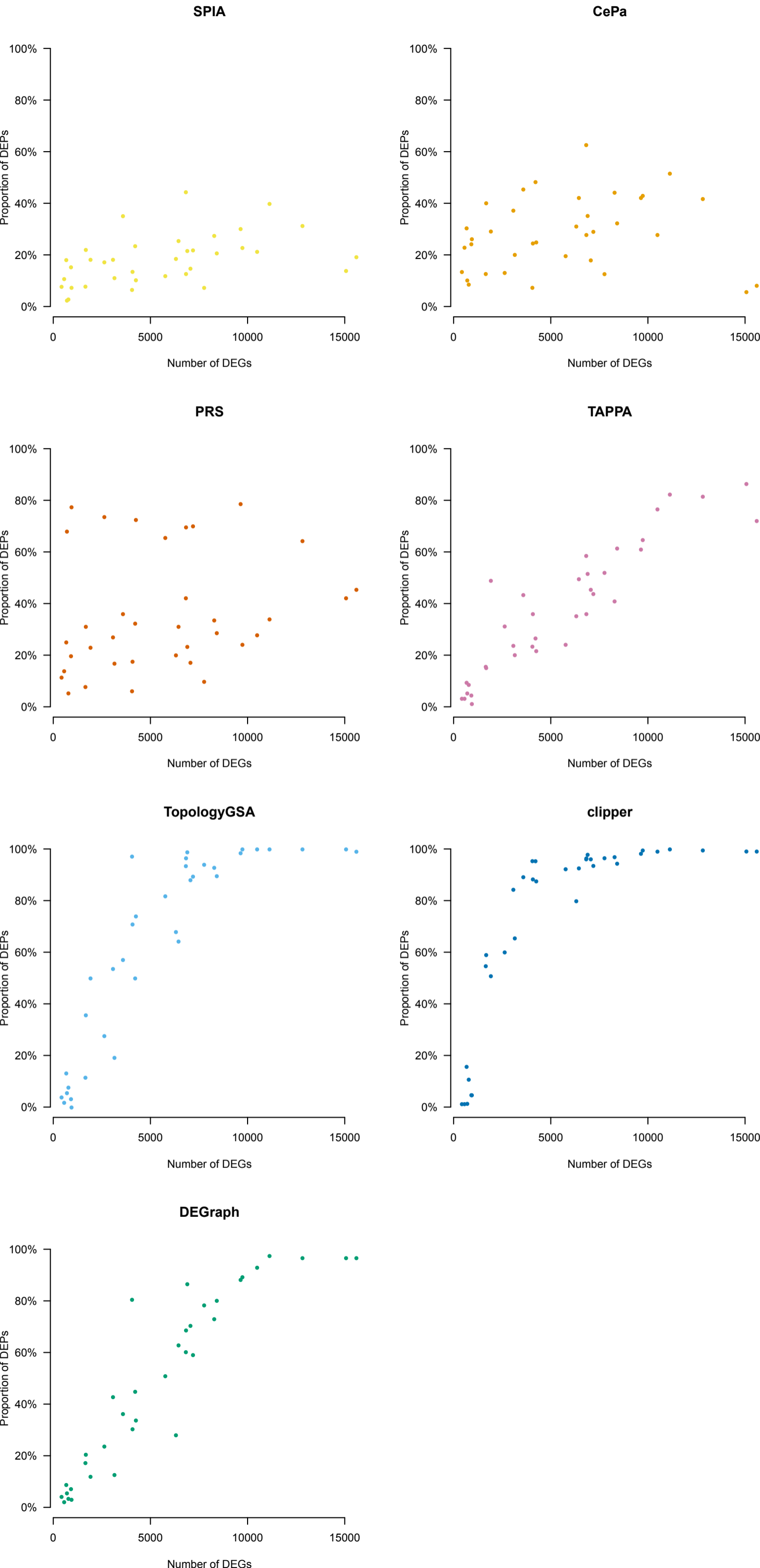

Supplement: S2 Fig — Proportion of DEPs depending on the number of DEGs for datasets from Disease-Control Data Collection. Each point represents one dataset. (PDF) [file pone.0191154.s003.pdf]

## Effect of the pathway size

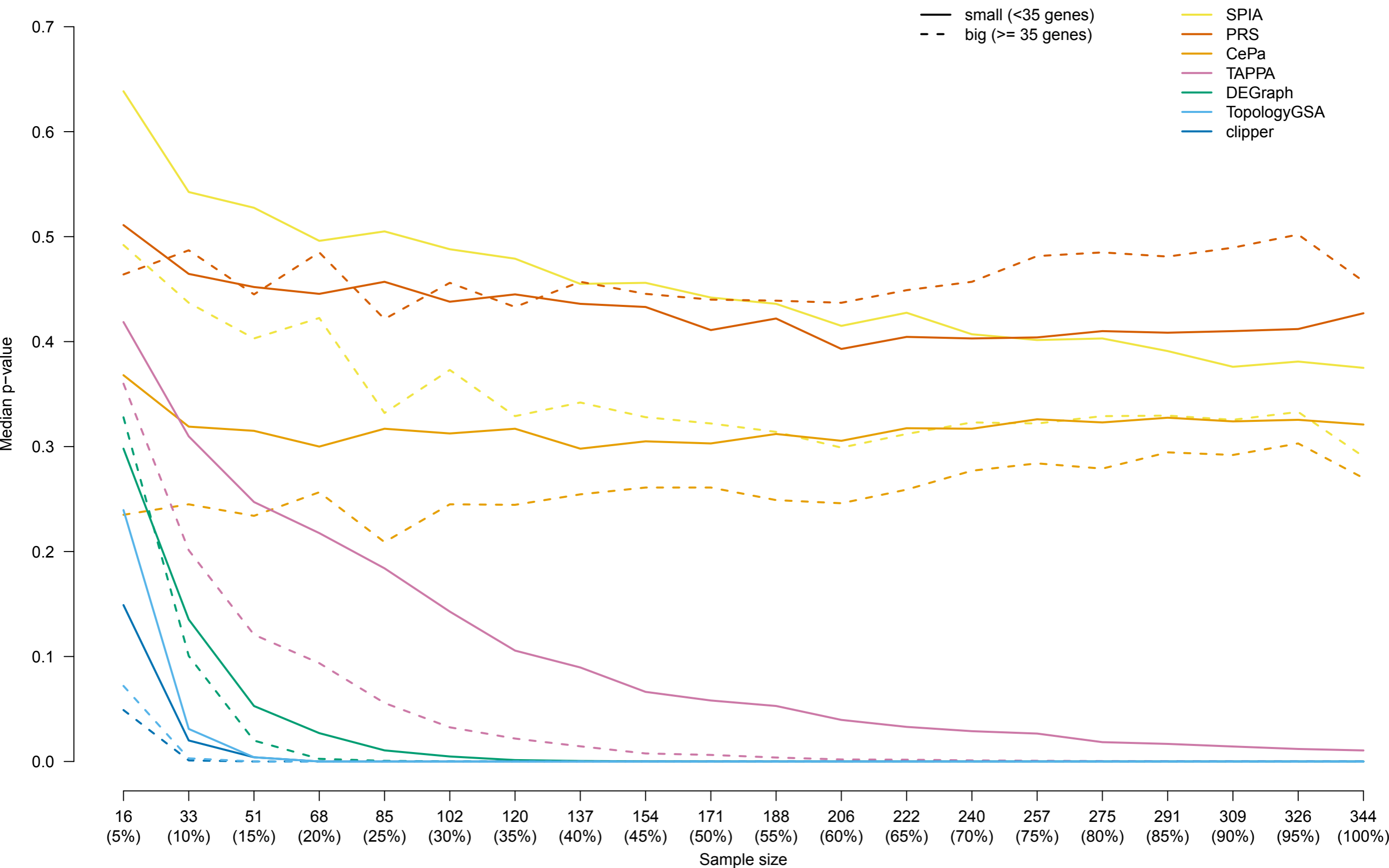

Supplement: S3 Fig — (PDF) [file pone.0191154.s004.pdf]

# Effect of the thresholds used for DEG detection

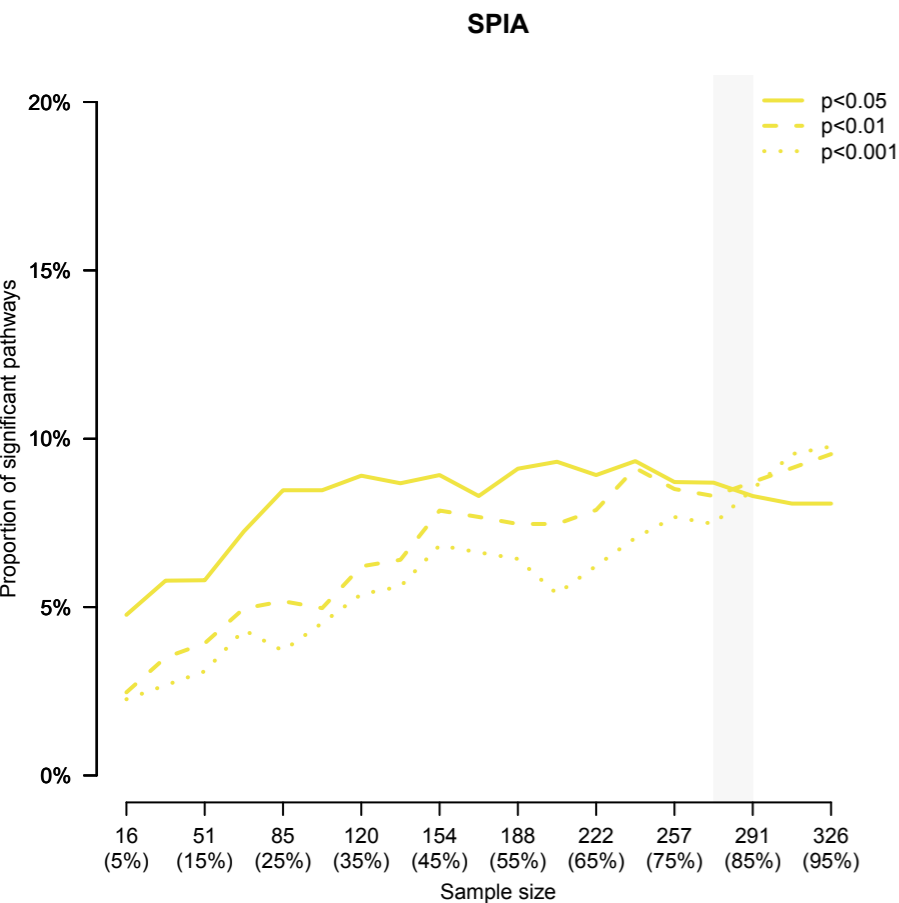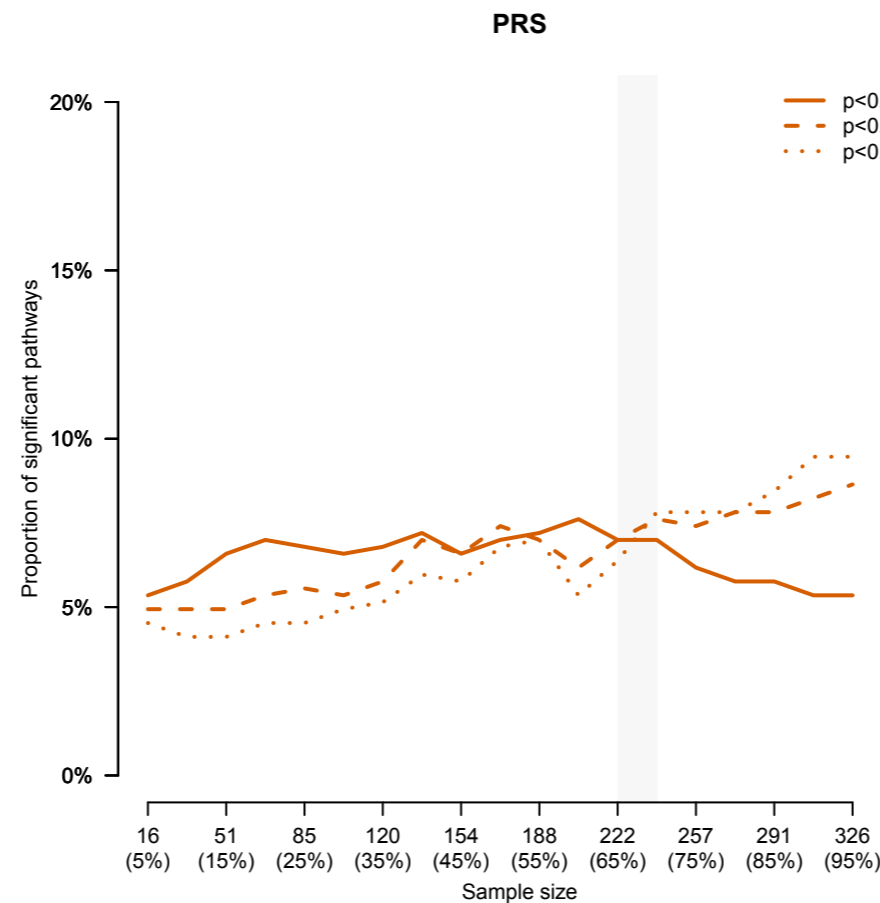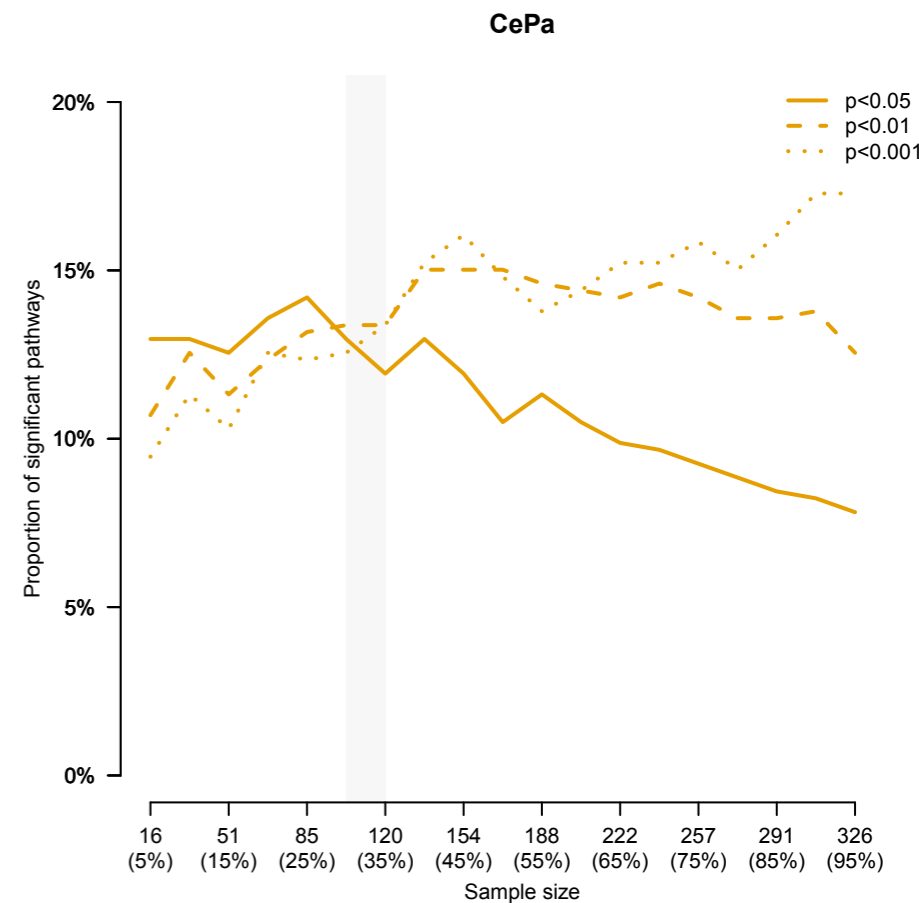

Supplement: S4 Fig — (PDF) [file pone.0191154.s005.pdf]

# Distribution of p-values from Experiment 2

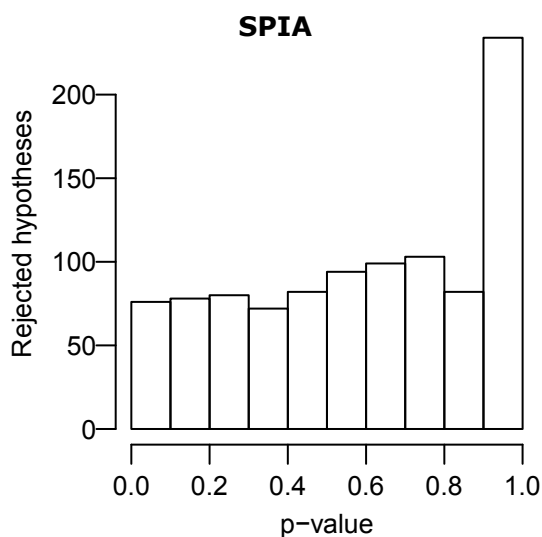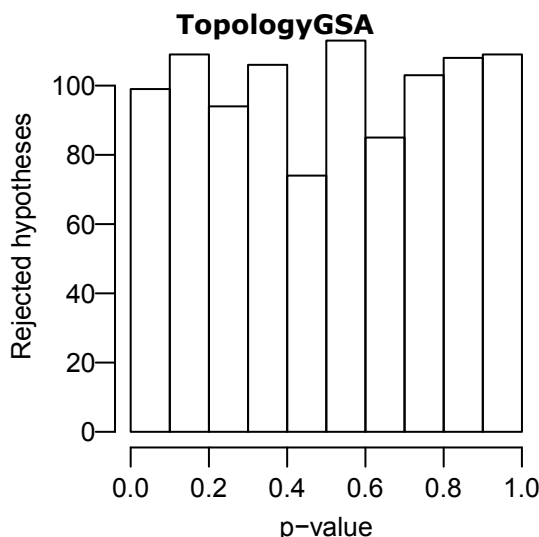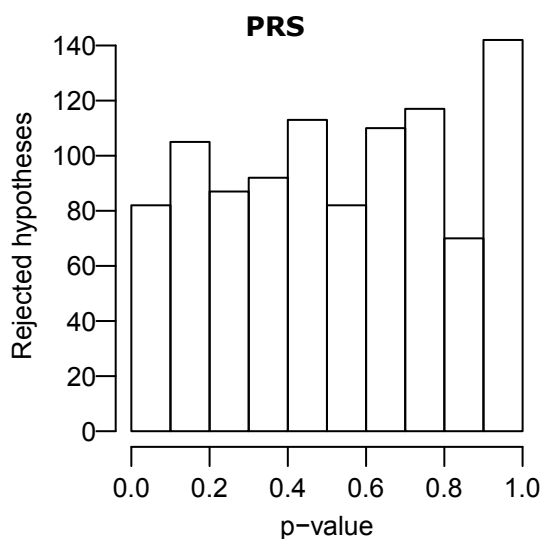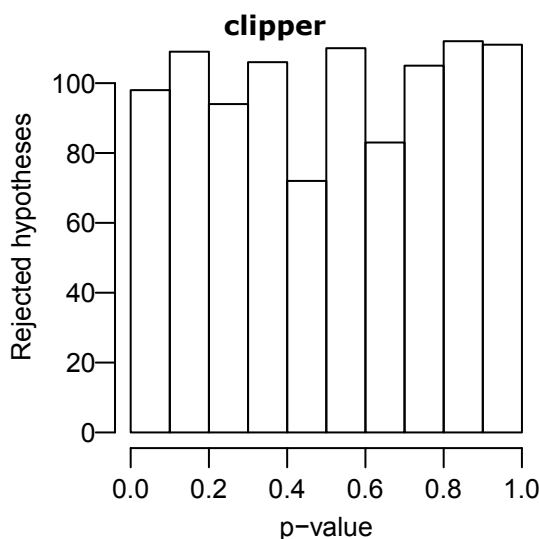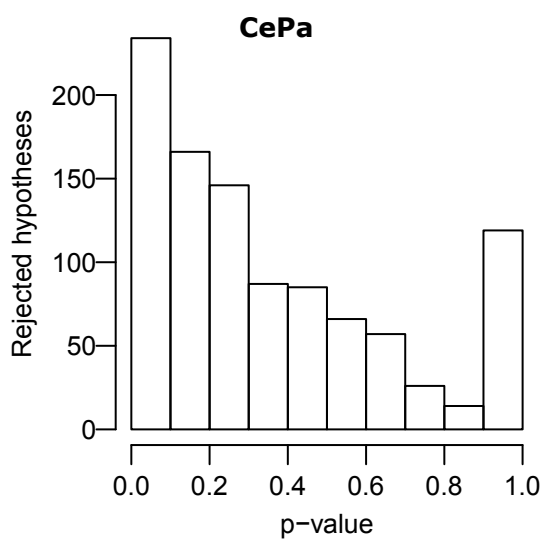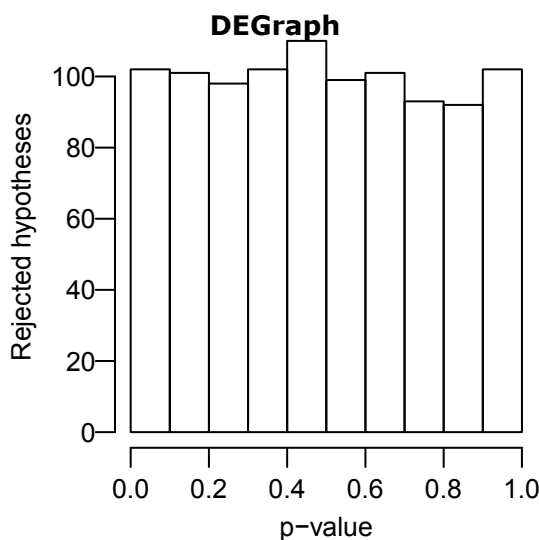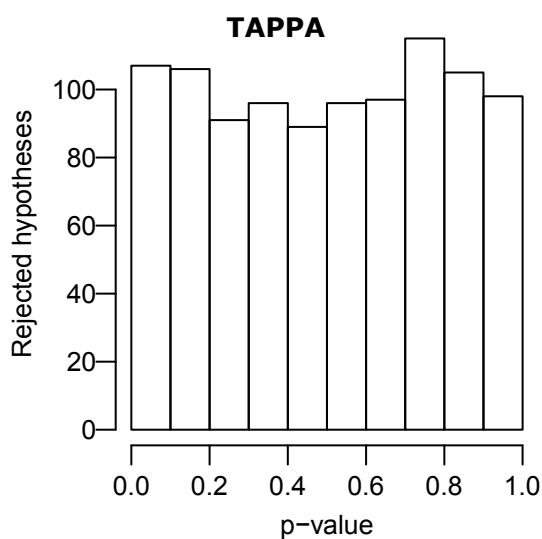

Supplement: S5 Fig — (PDF) [file pone.0191154.s006.pdf]

**SPIA**

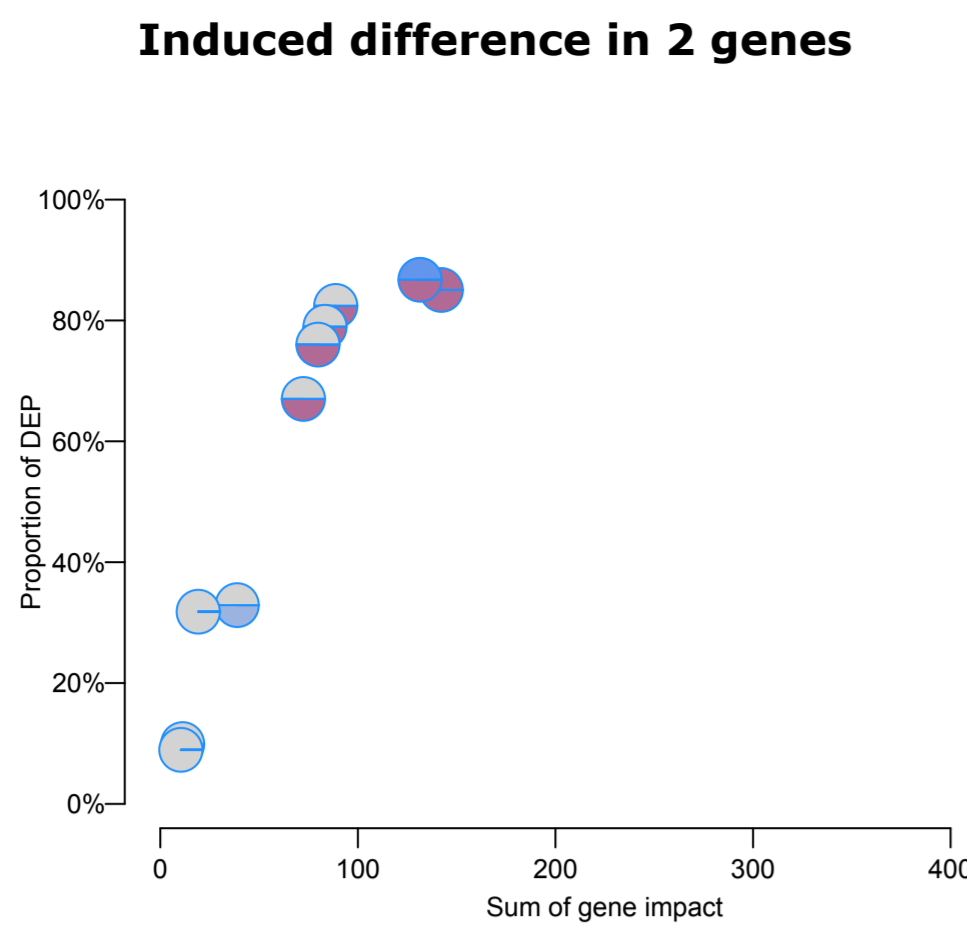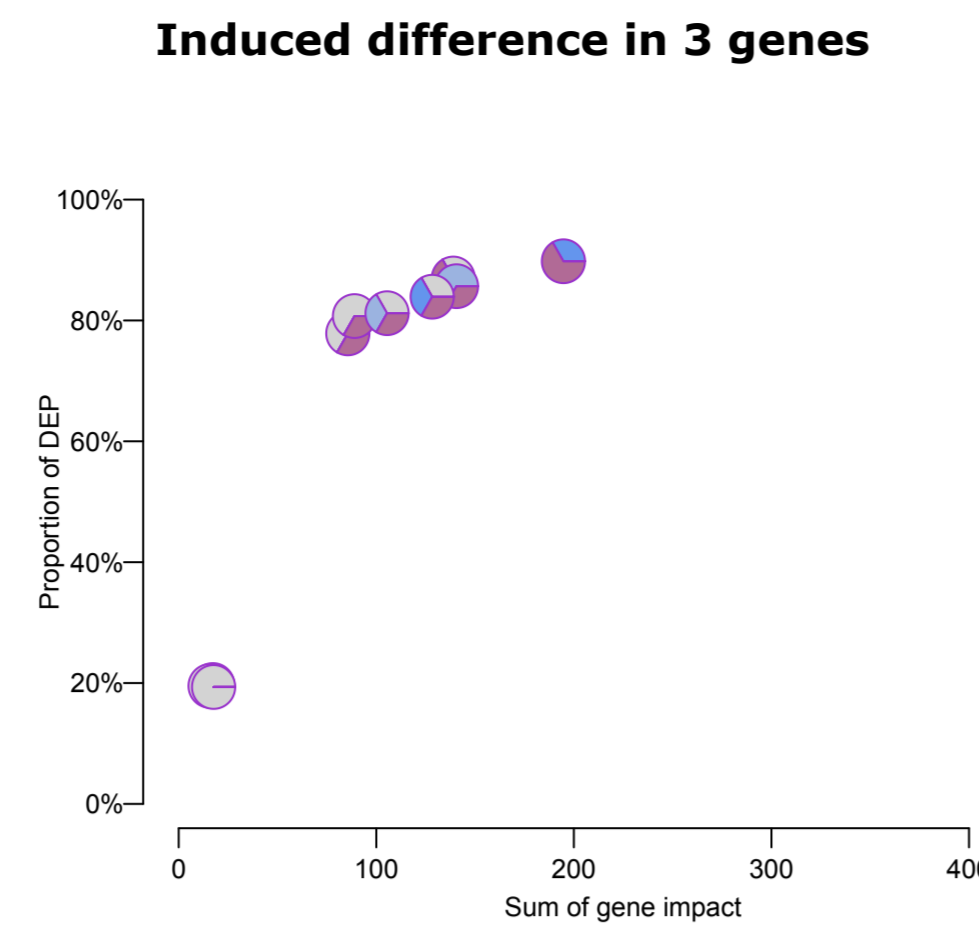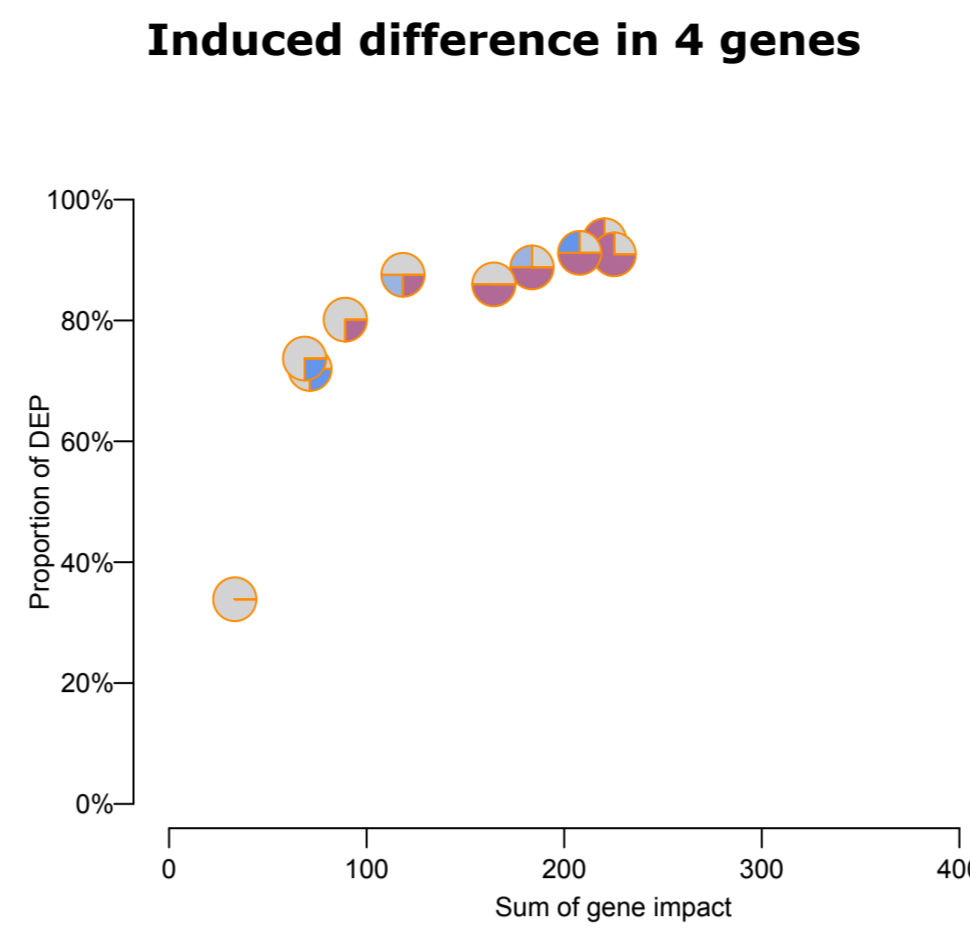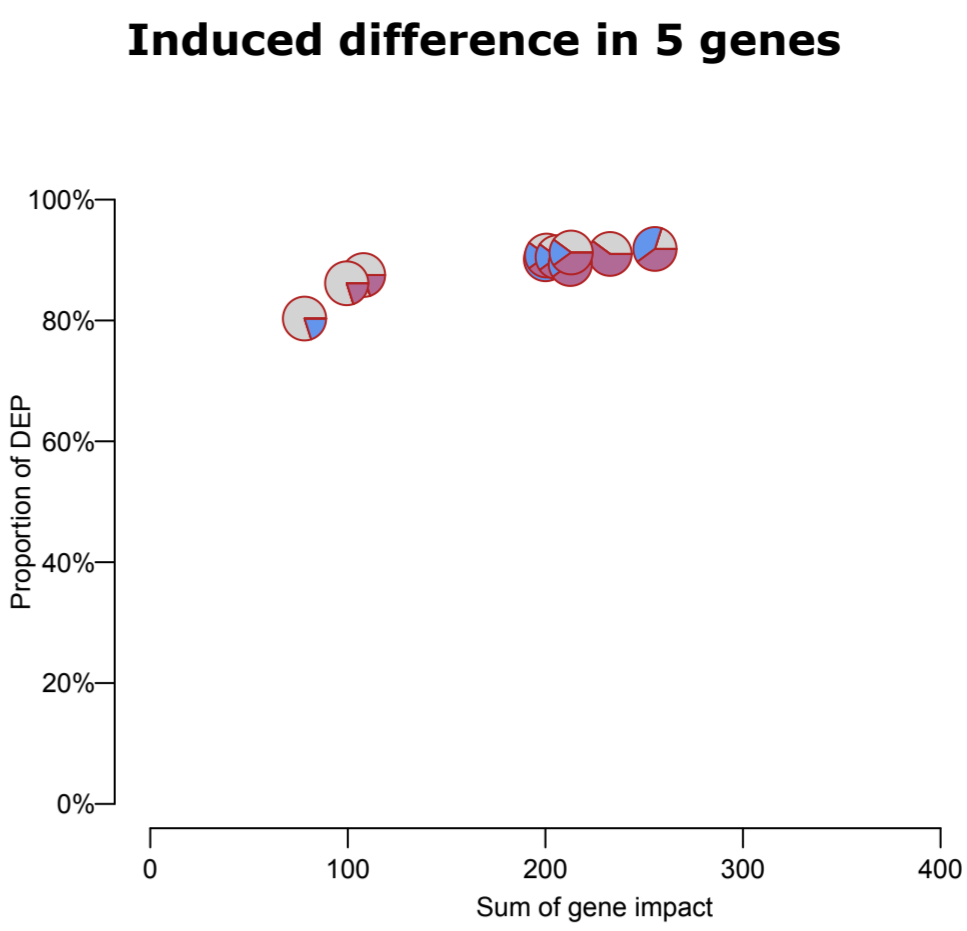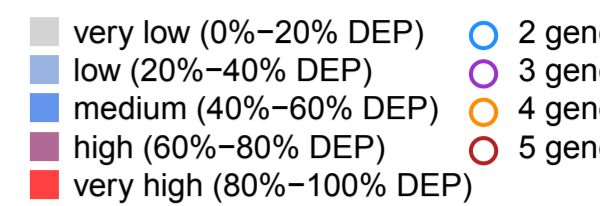

**PRS**

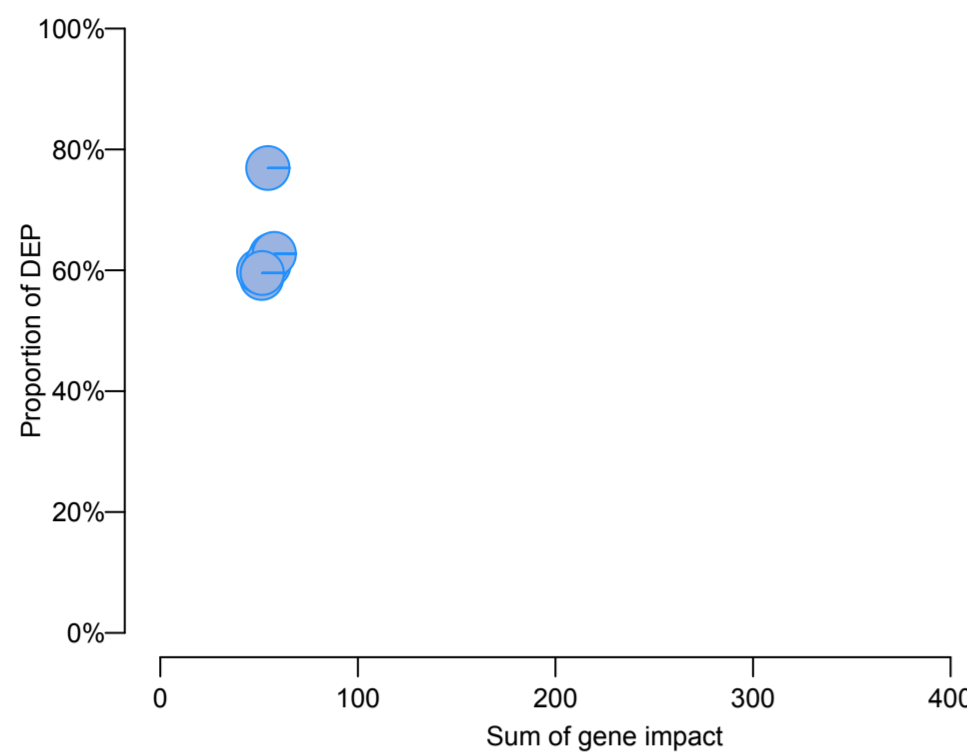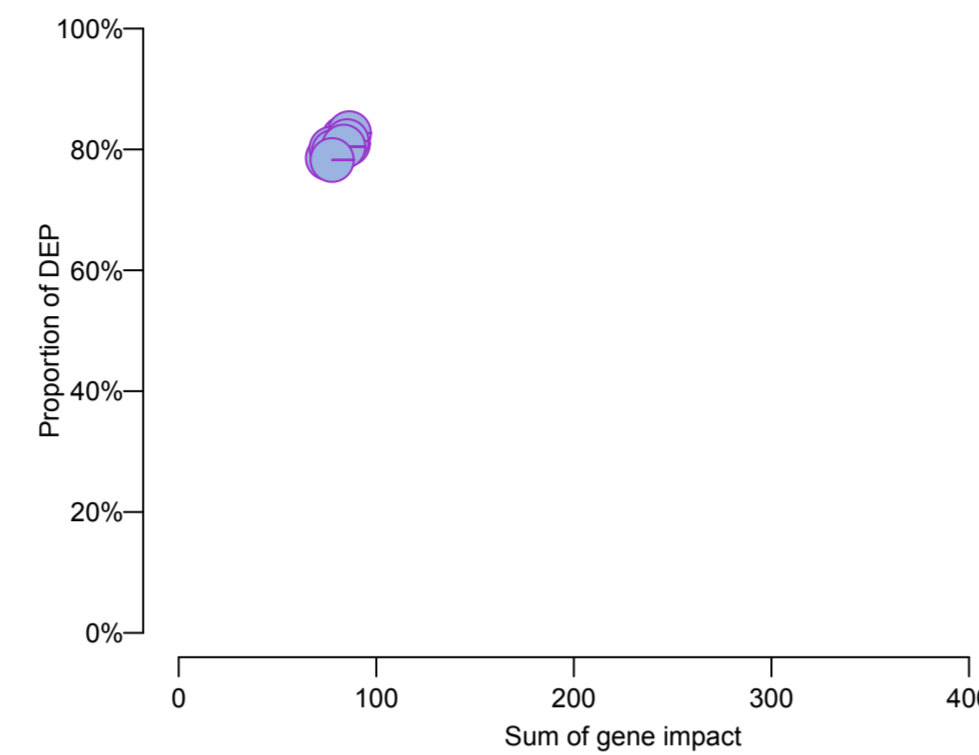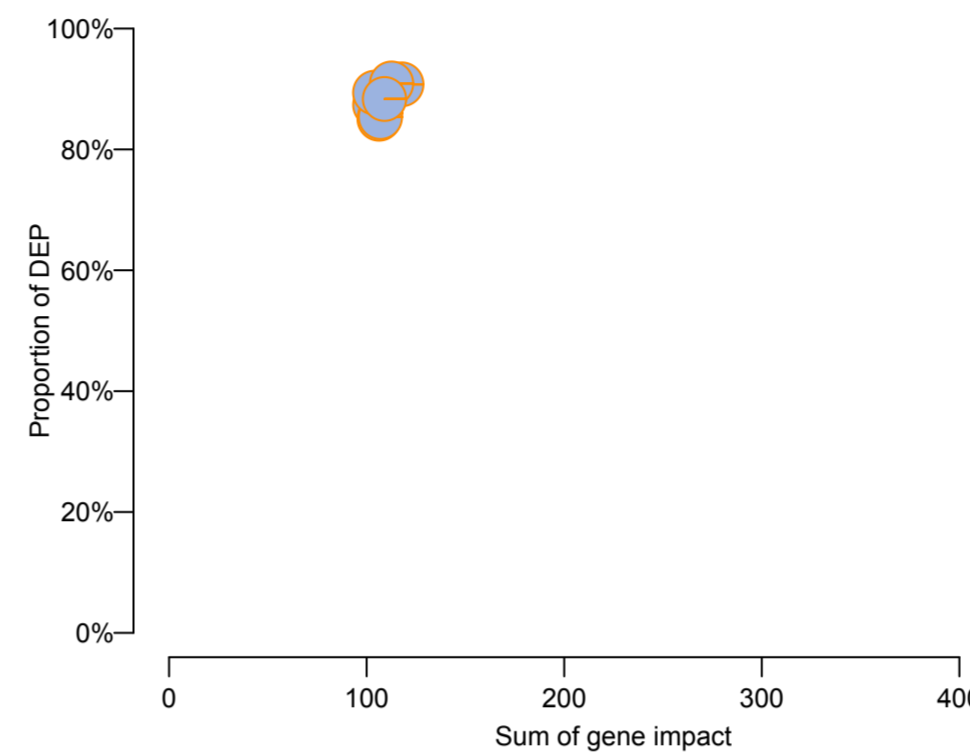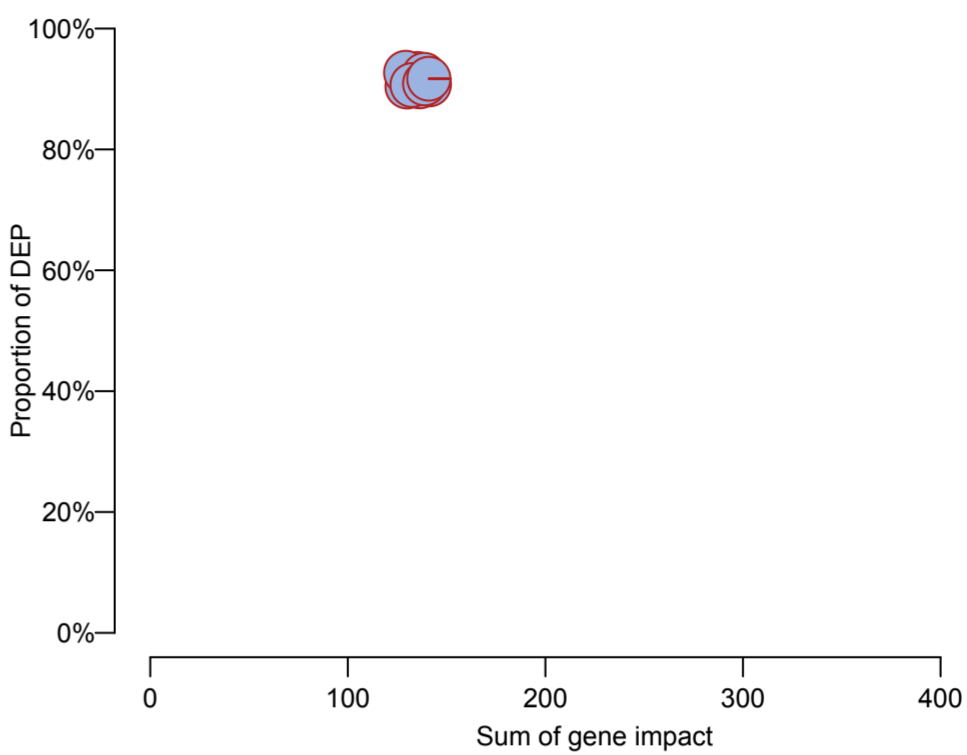

**CePa**

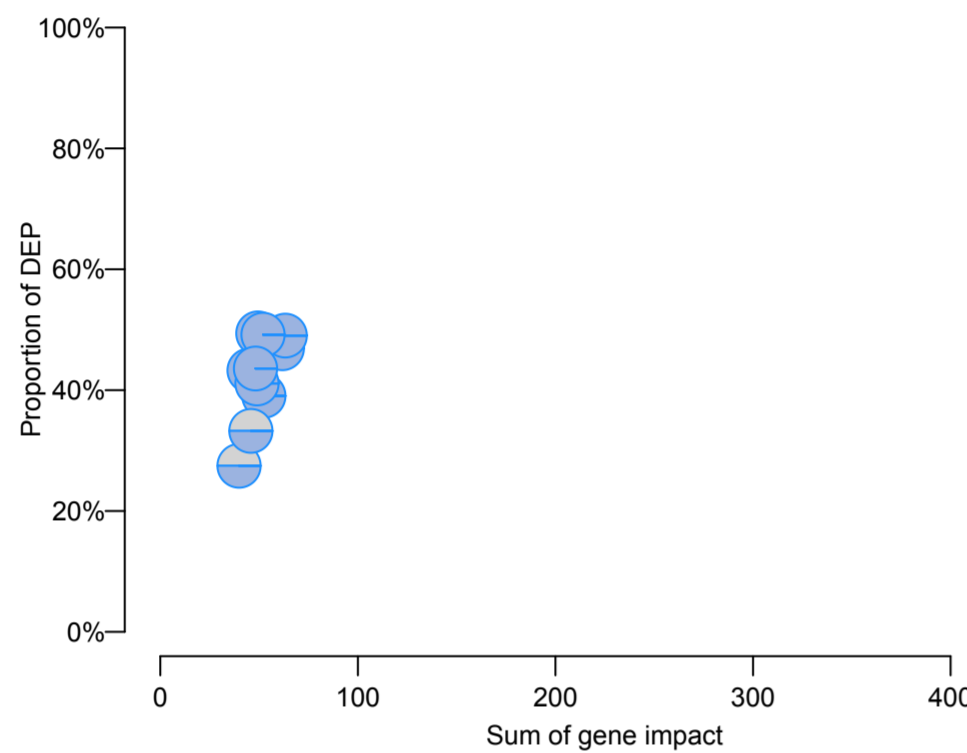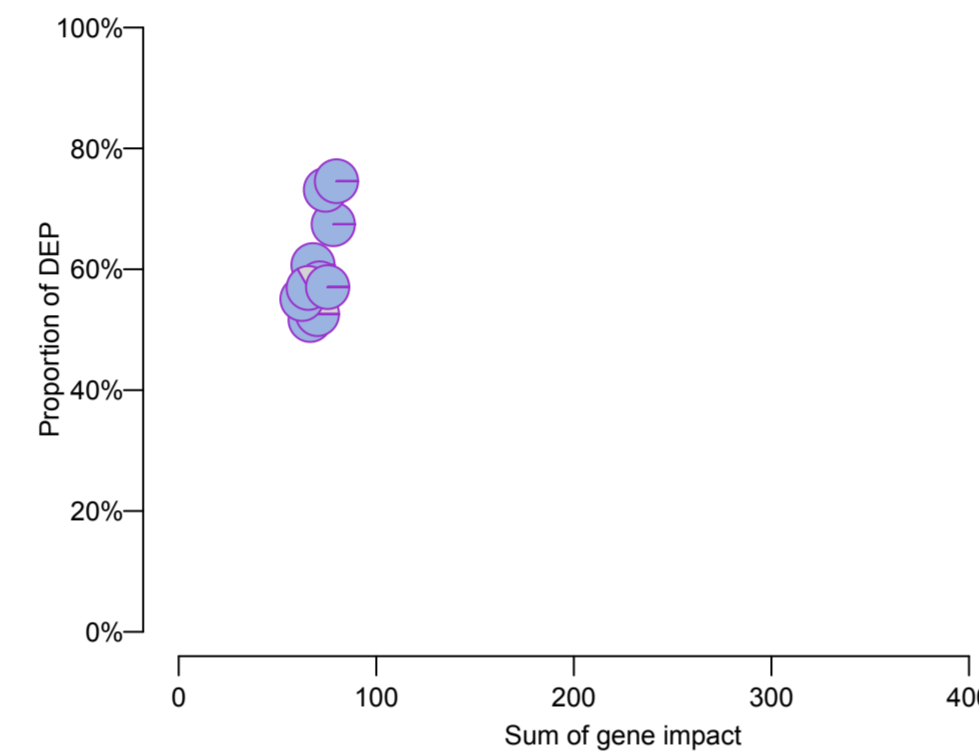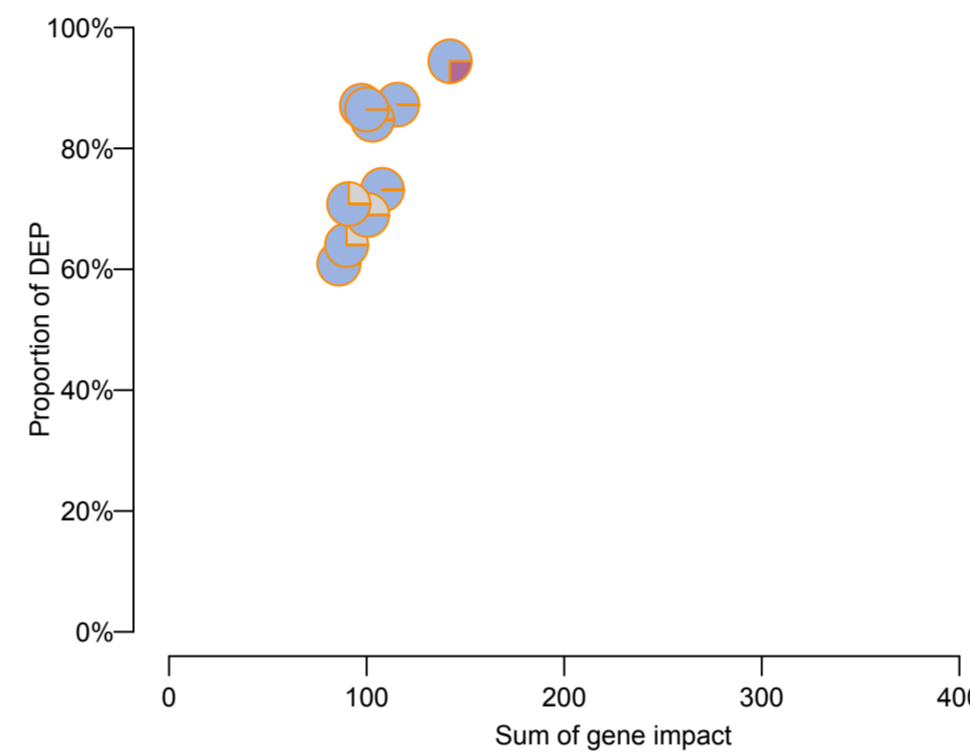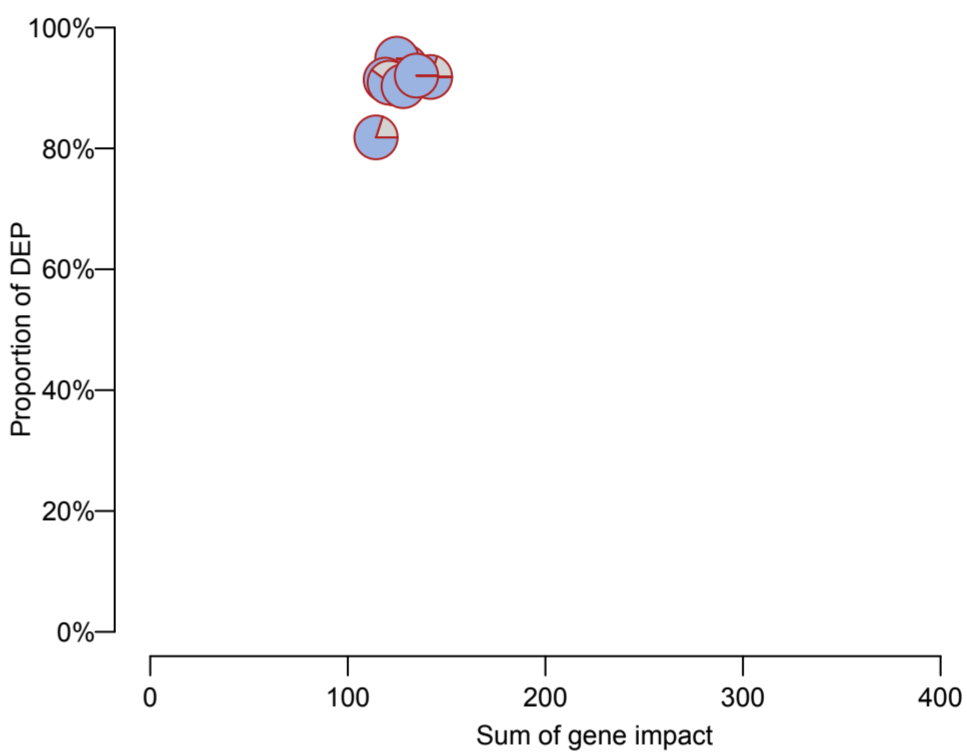

**TAPPA**

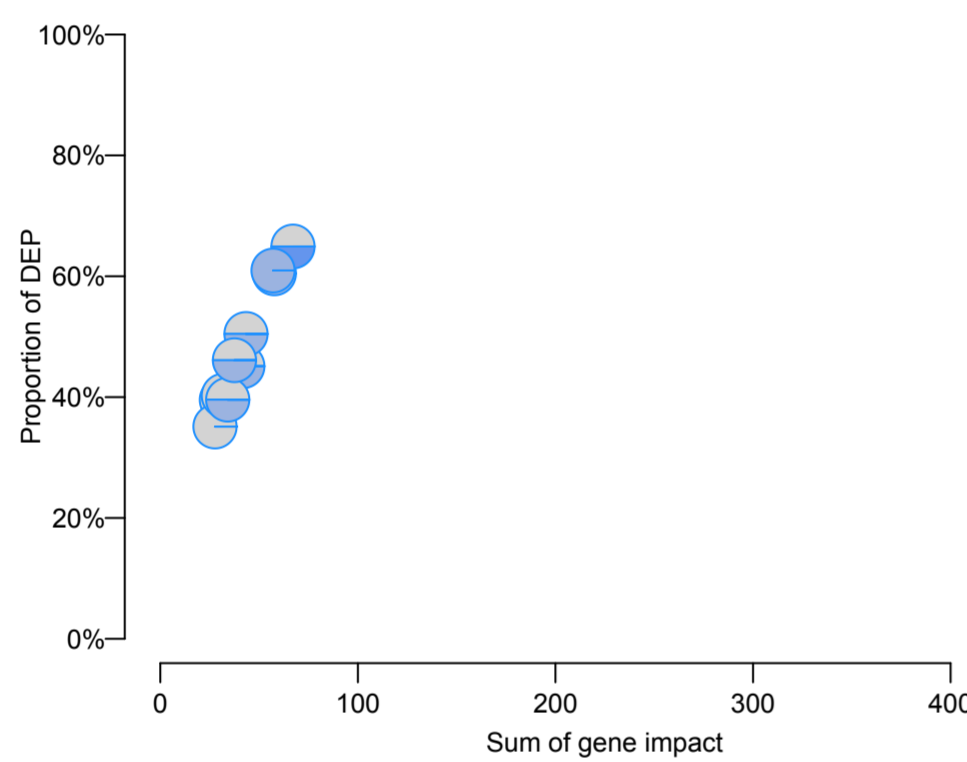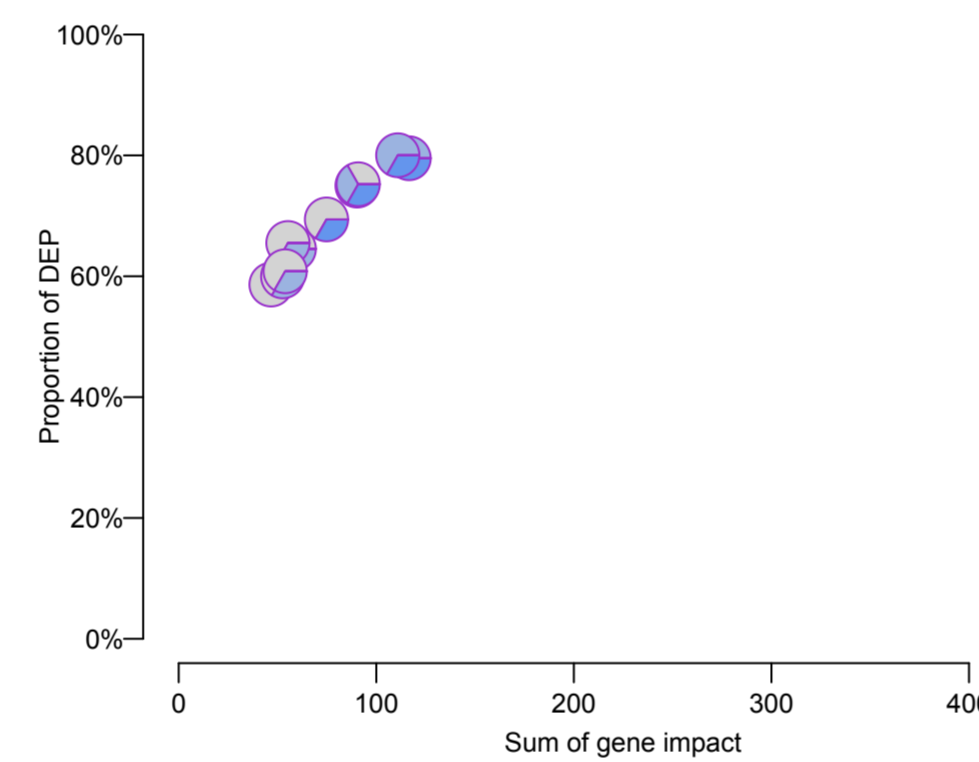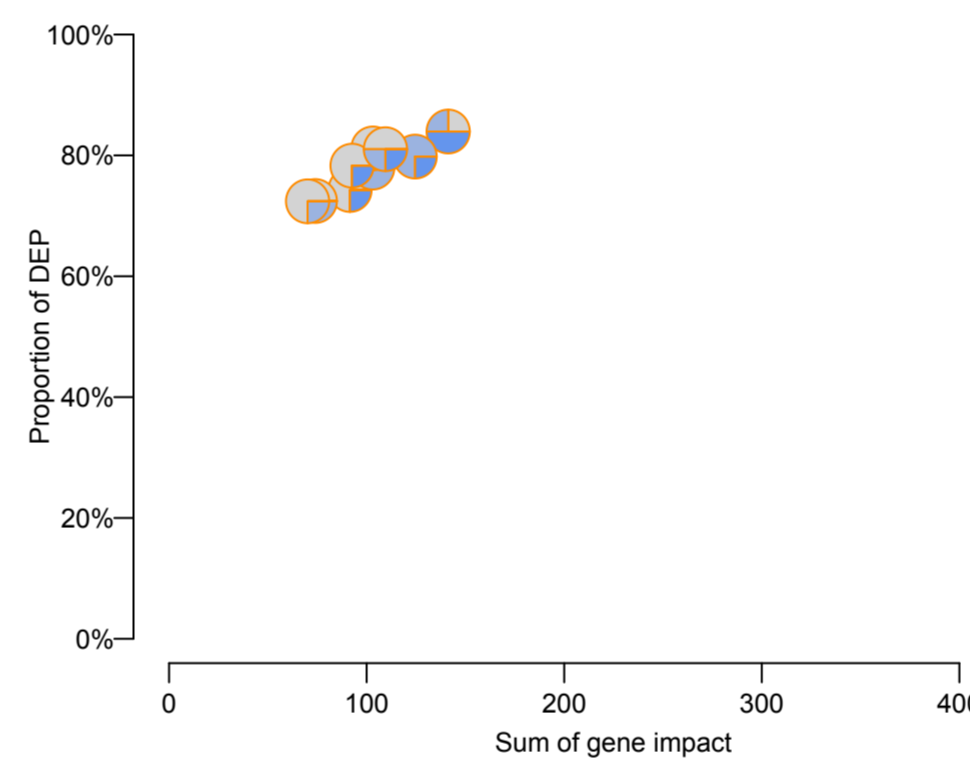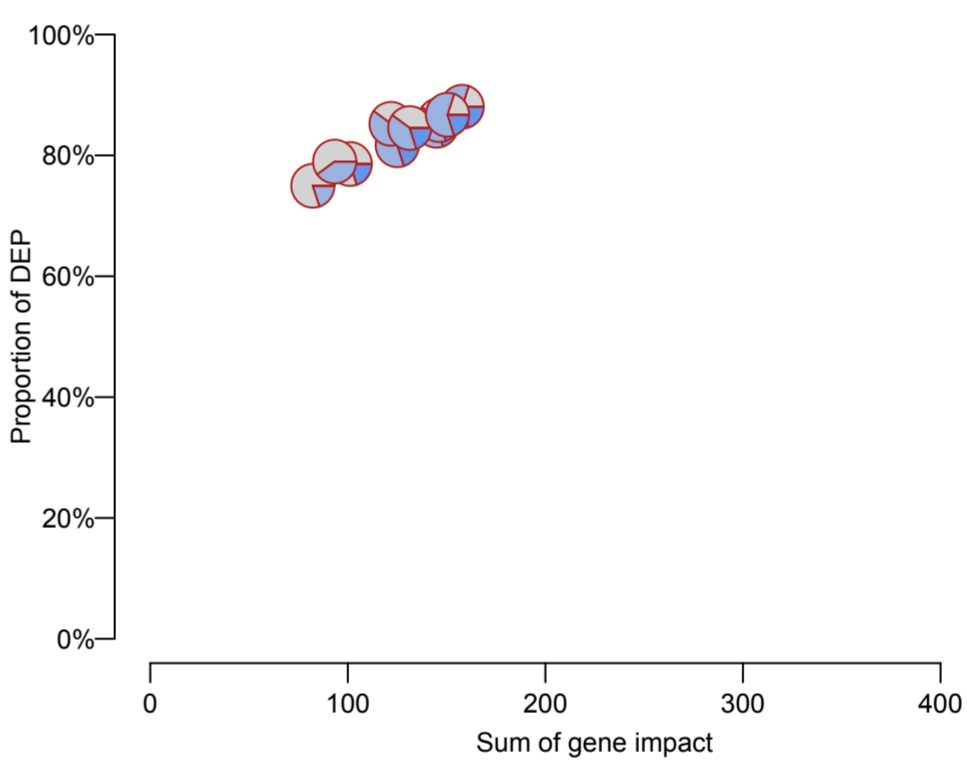

**TopologyGSA**

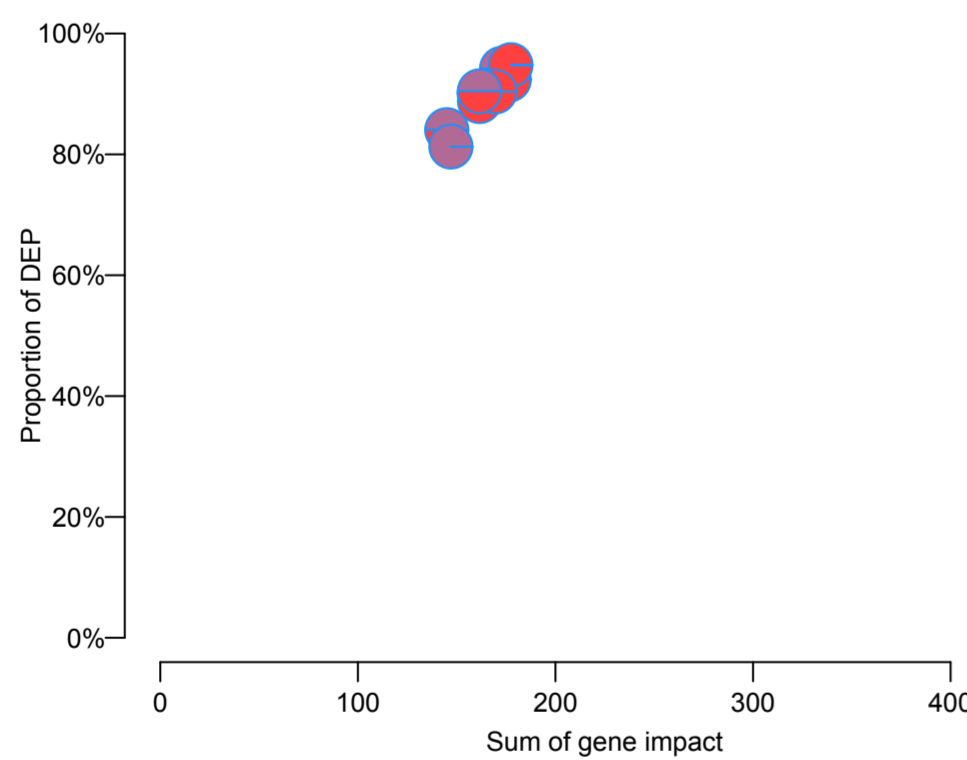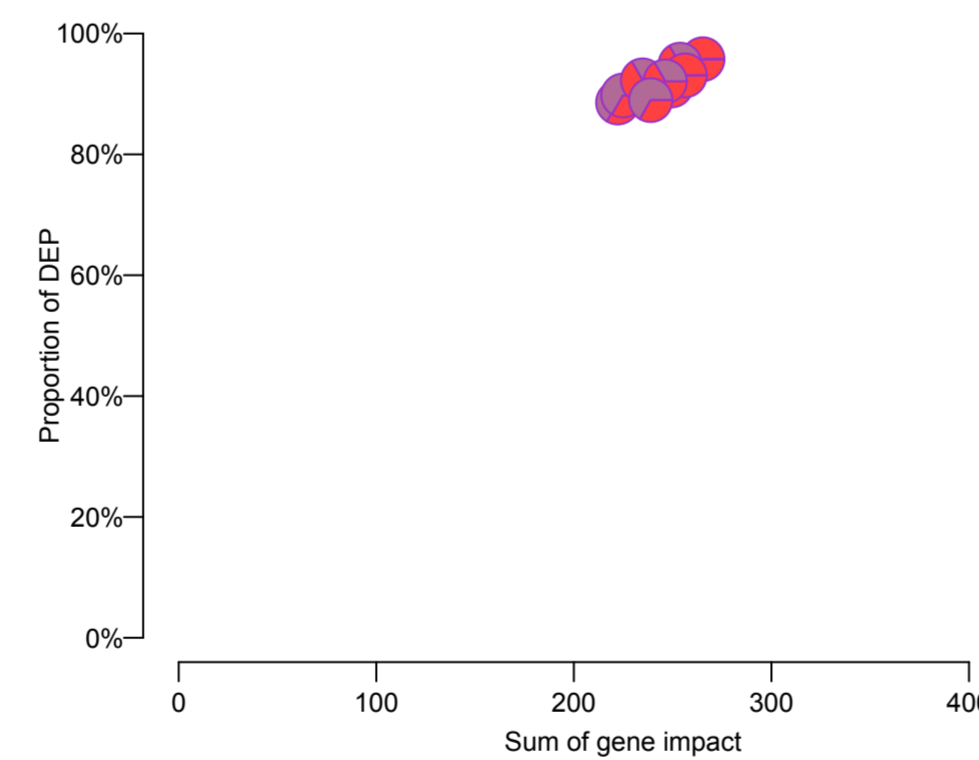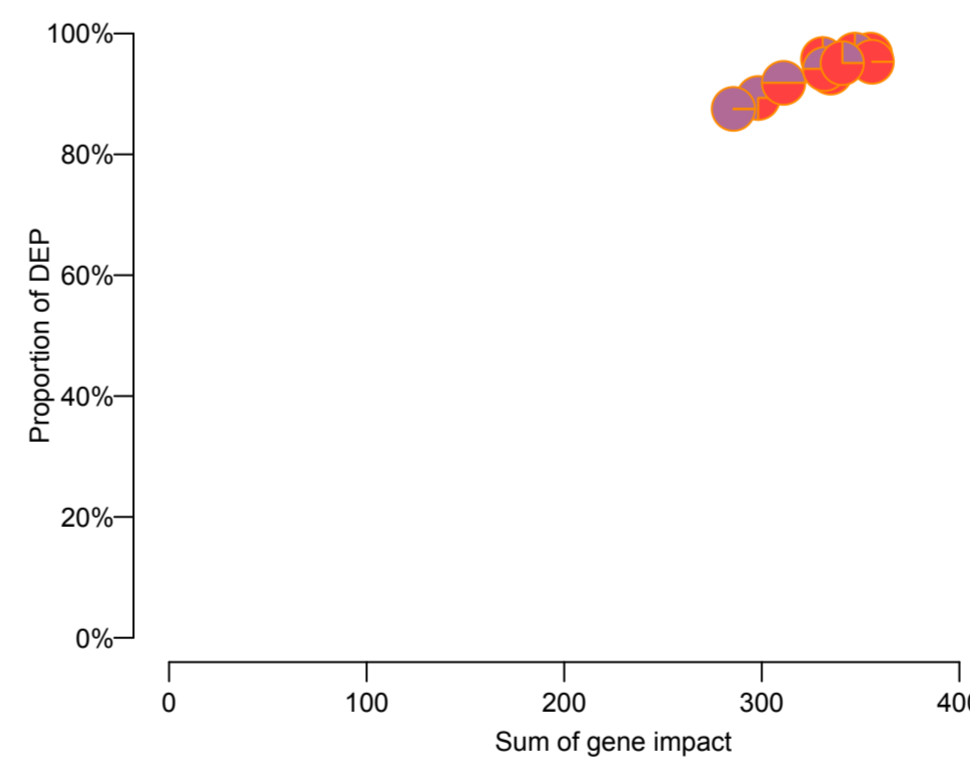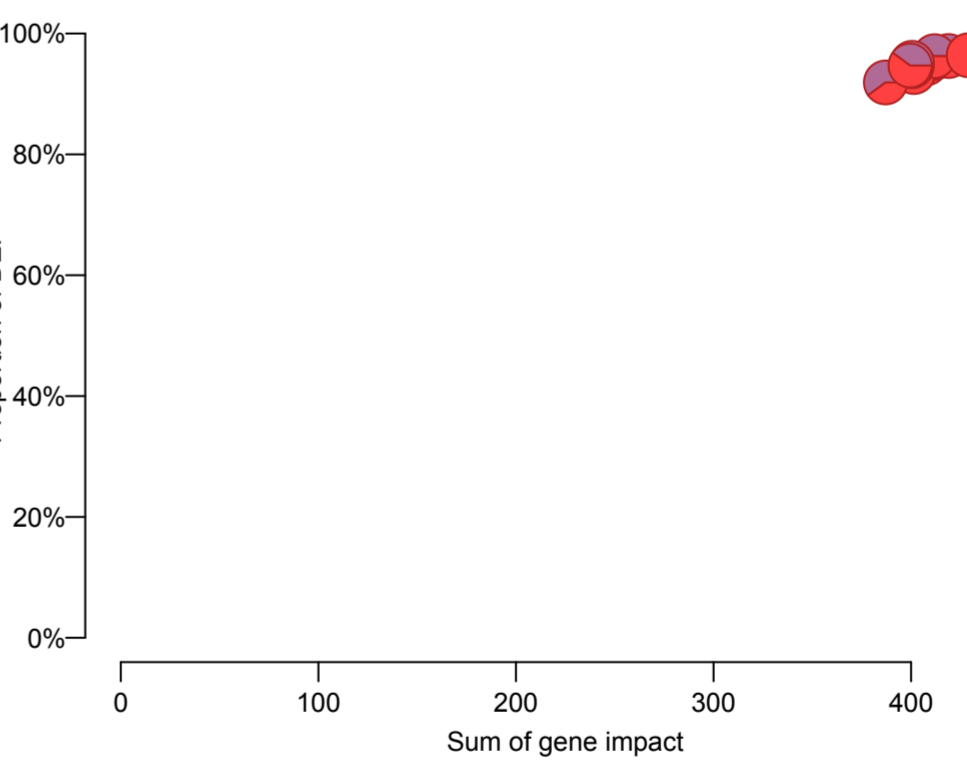

**clipper**

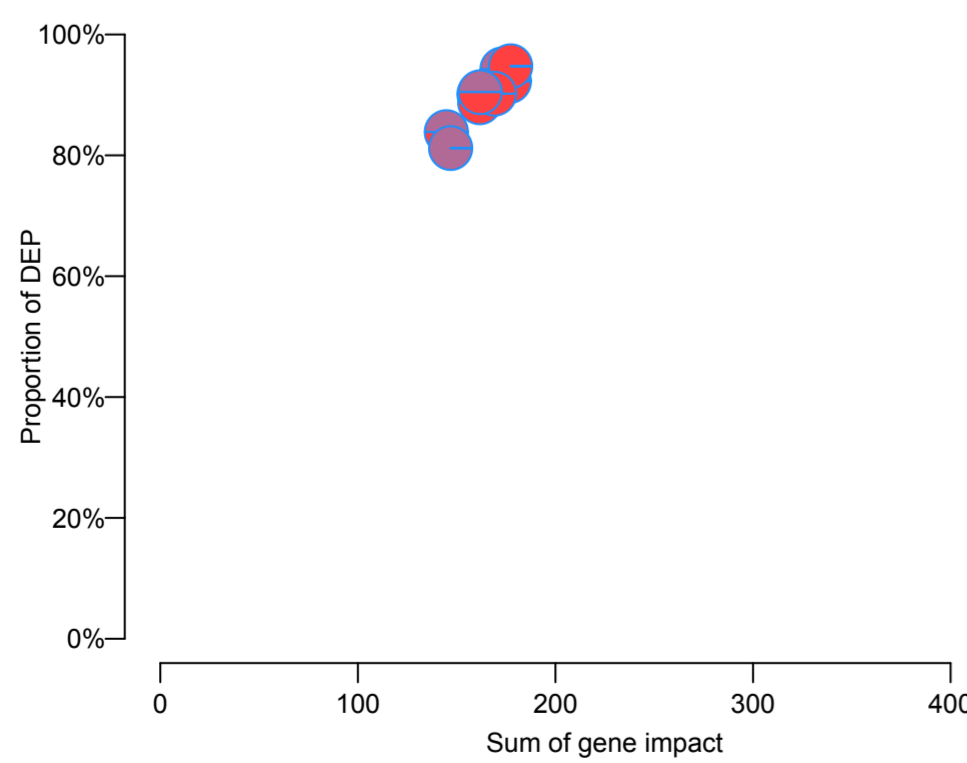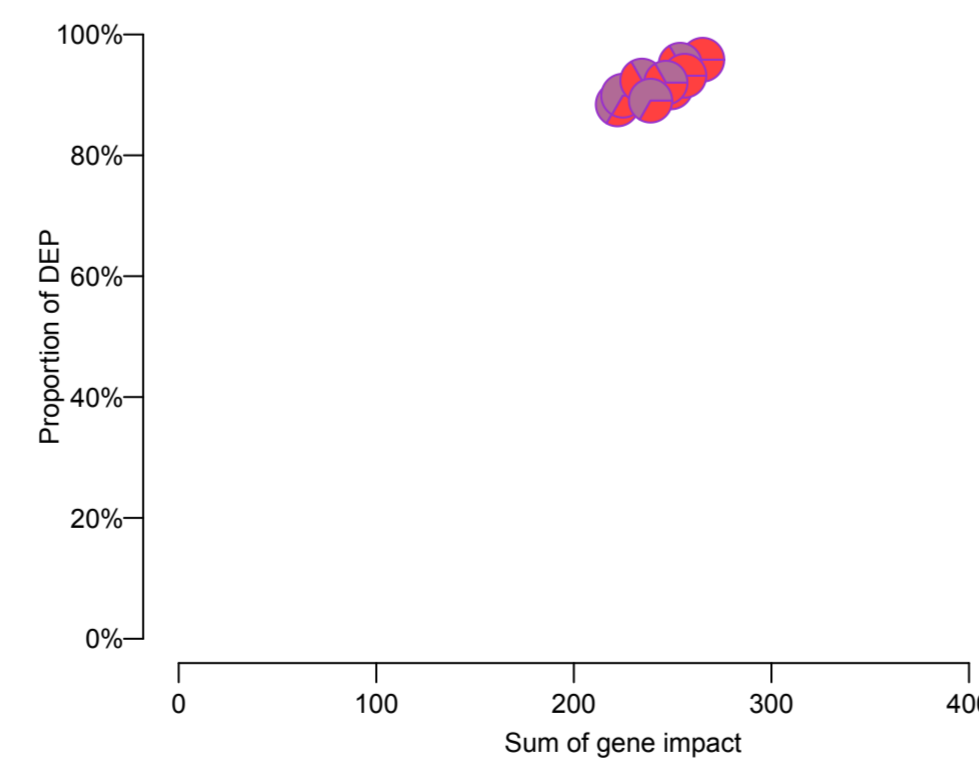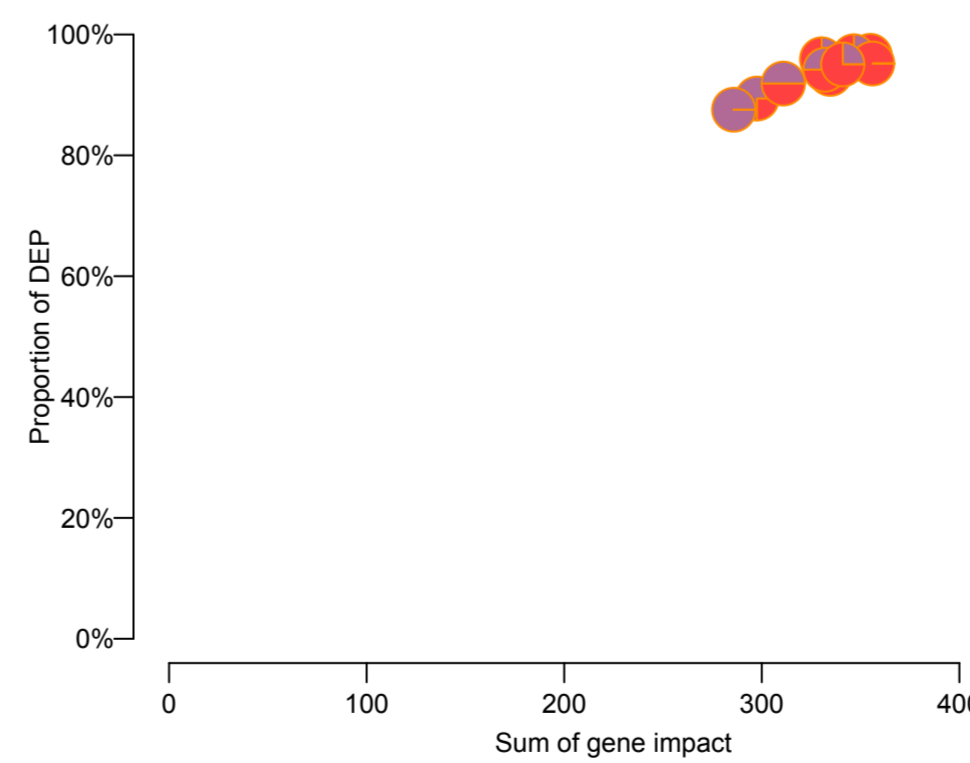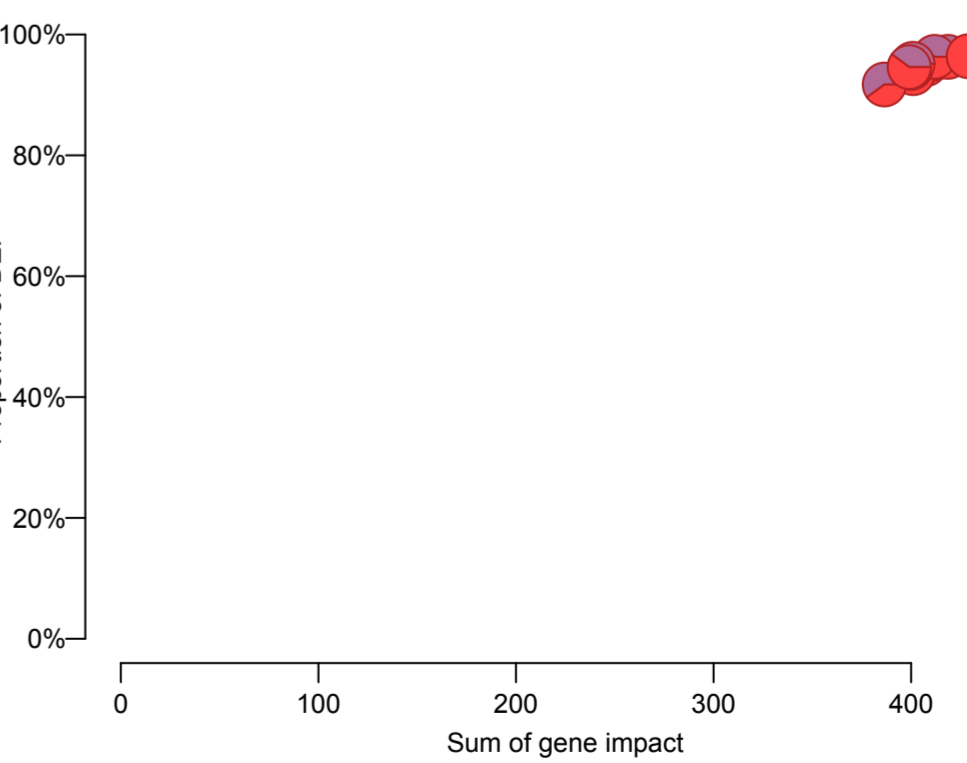

**DEGraph**

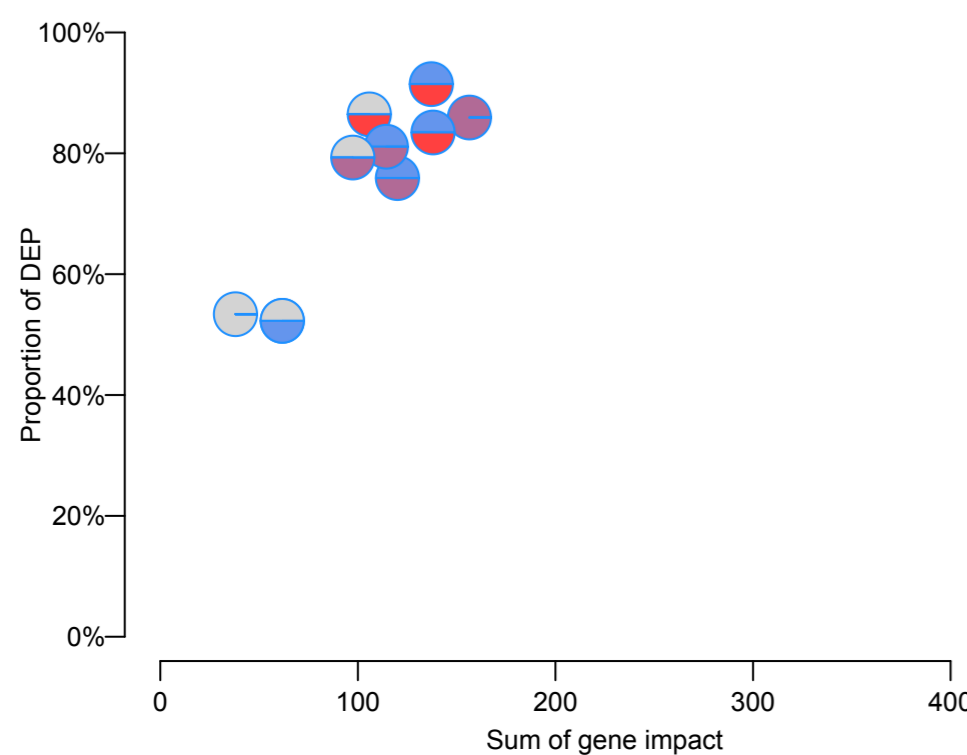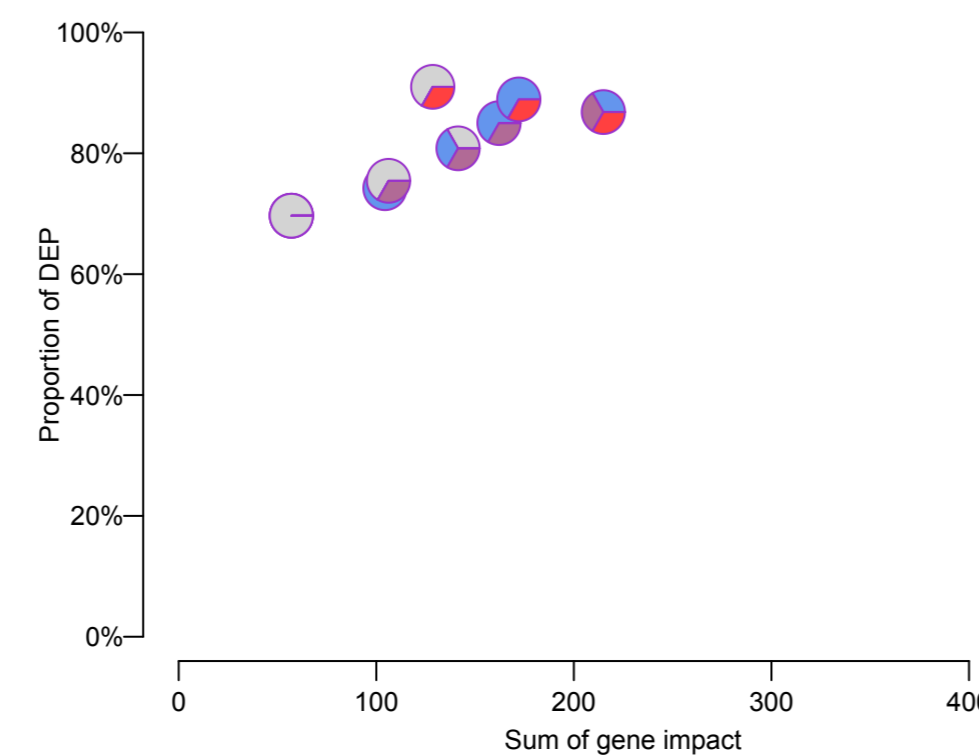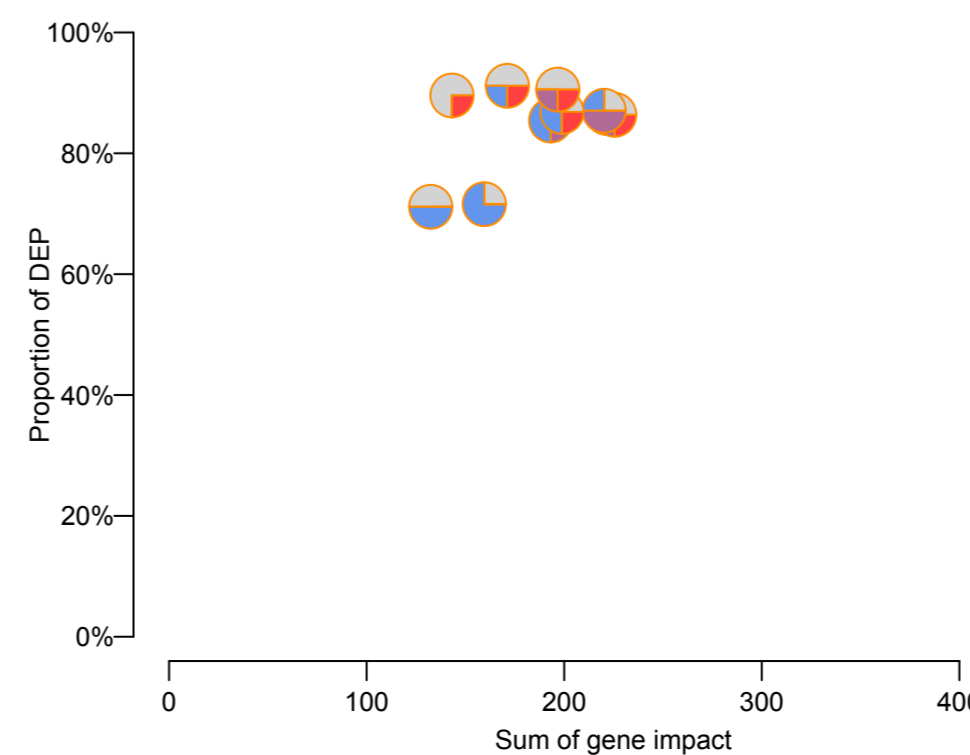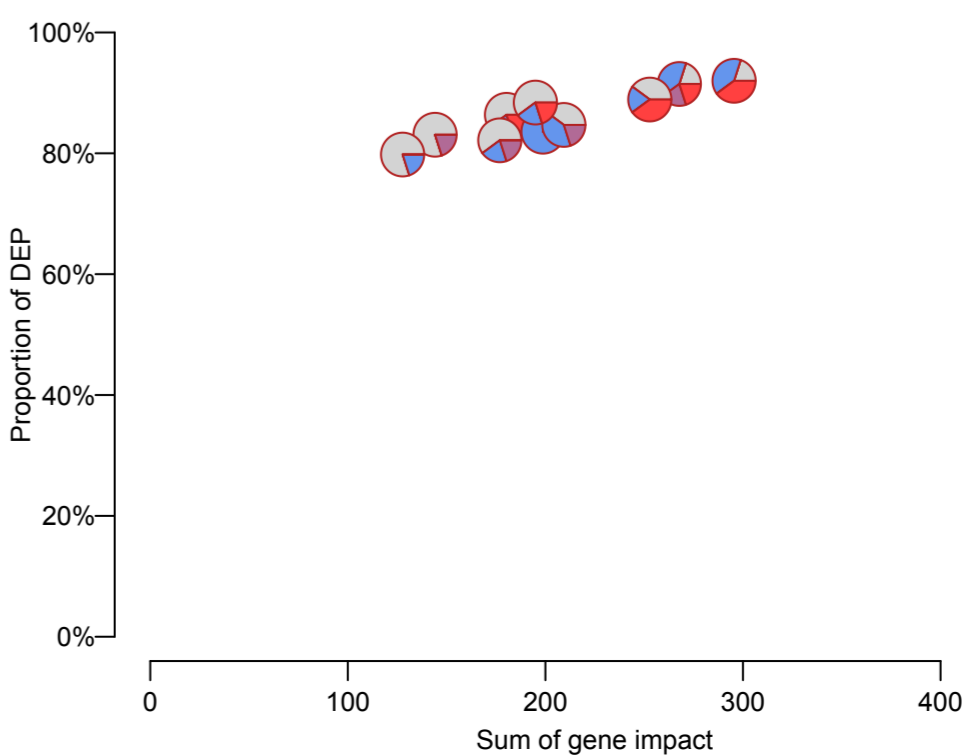

Supplement: S7 Fig — Sets of 2, 3, 4 and 5 genes were randomly selected from Non-small cell lung cancer pathway. Each circle represents one of those sets. Expression of genes in the set was modified with increments of 0.1 to 2 with step size 0.1 in 200 simulated datasets. Border color indicates number of genes in the set. Vertical axis shows combined influence of the genes (proportion of differentially expressed pathways across all increments and datasets). Horizontal axis corresponds to sum of the influence of individual genes. Pie color (from grey to blue and red) represents the influence of a single gene (see Experiment 3 for details). (PDF) [file pone.0191154.s008.pdf]

# Summarization of the Experiment 5 - CePa

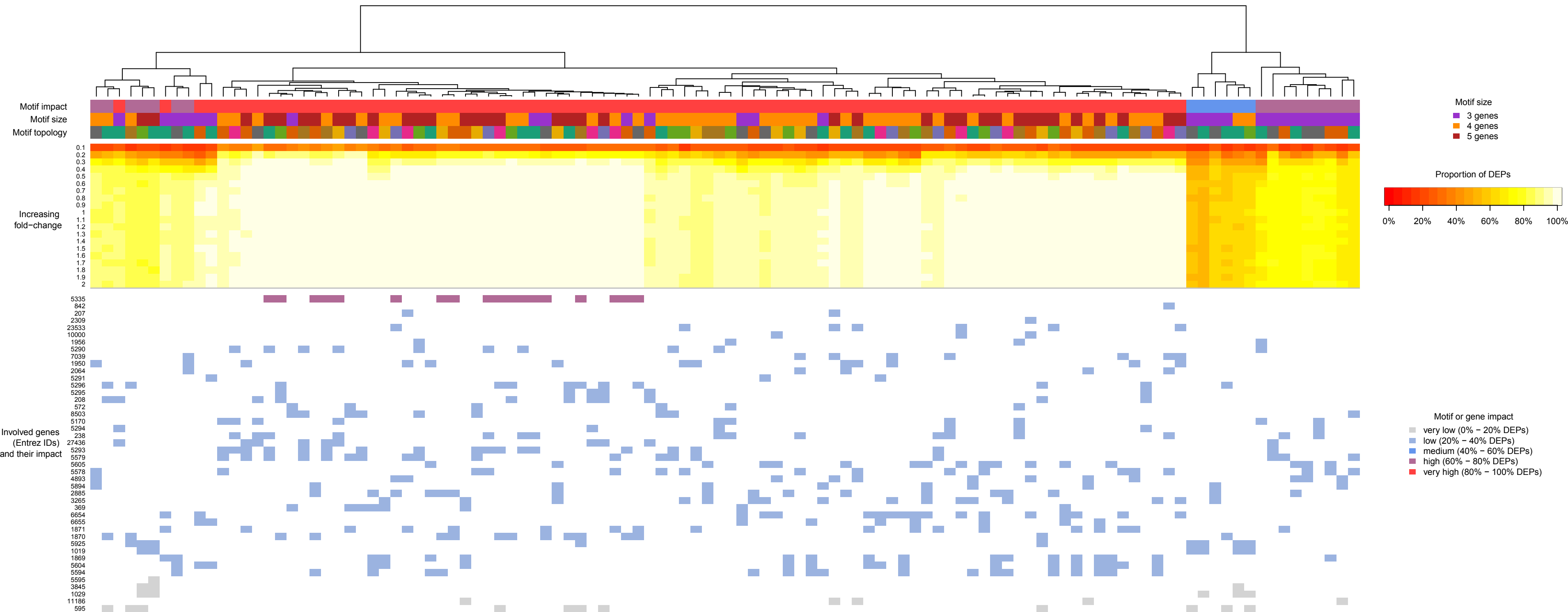

Supplement: S8 Fig — Heatmaps of the proportion of DEPs for all compared methods. (ZIP) [file pone.0191154.s009.zip › S9 Fig/motifs_CePa.pdf]

# Summarization of the Experiment 5 - Clipper

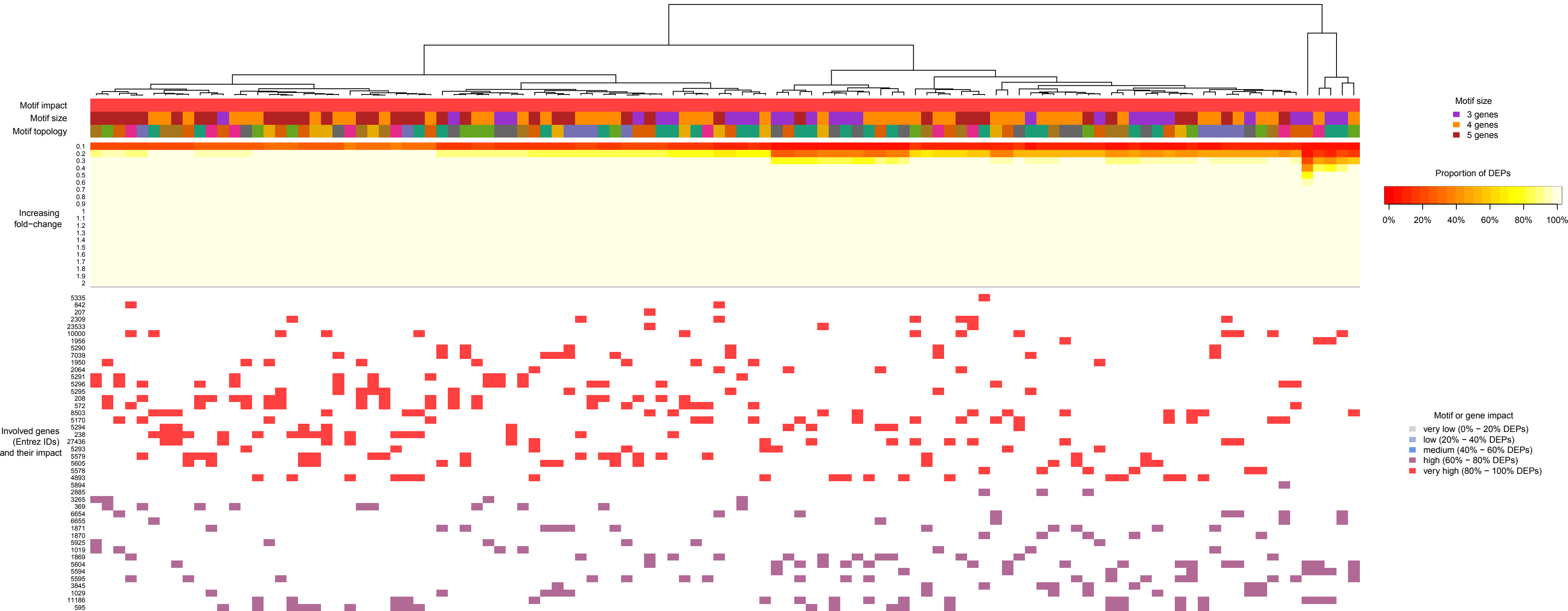

Supplement: S8 Fig — Heatmaps of the proportion of DEPs for all compared methods. (ZIP) [file pone.0191154.s009.zip › S9 Fig/motifs_clipper.pdf]

# Summarization of the Experiment 5 - DEGraph

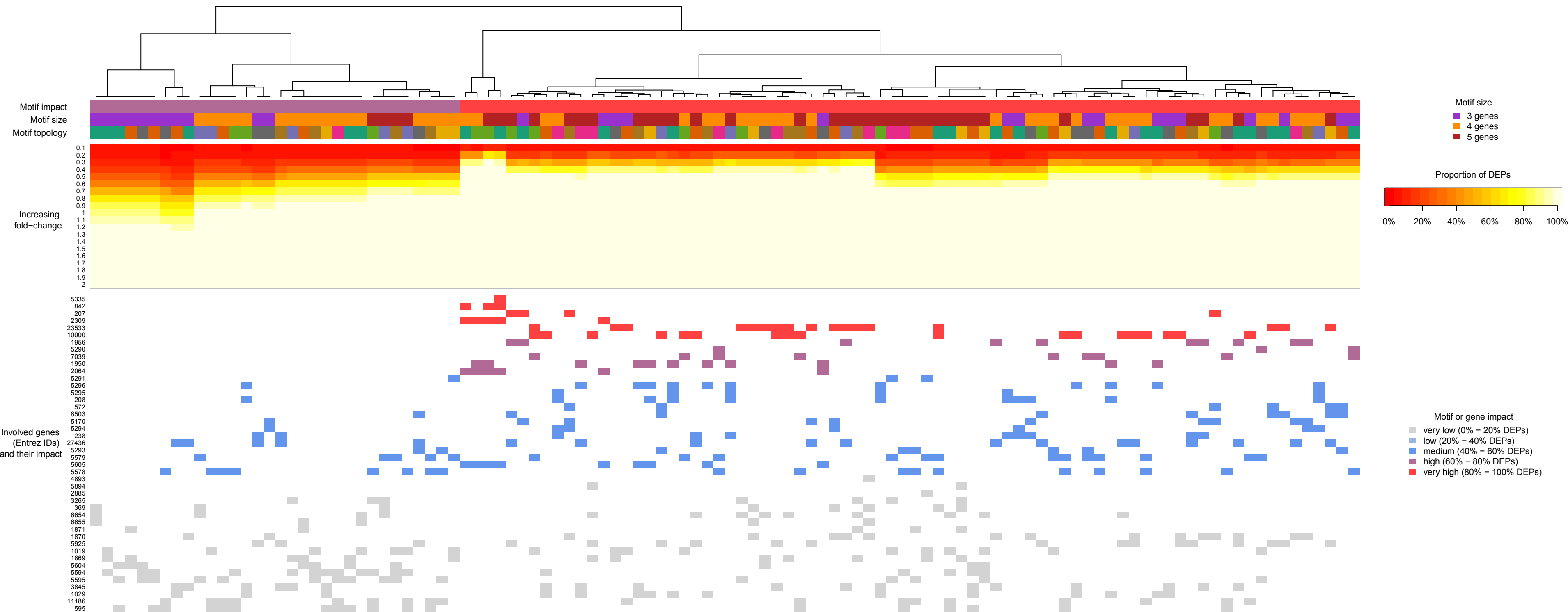

Supplement: S8 Fig — Heatmaps of the proportion of DEPs for all compared methods. (ZIP) [file pone.0191154.s009.zip › S9 Fig/motifs_DEGraph.pdf]

# Summarization of the Experiment 5 - PRS

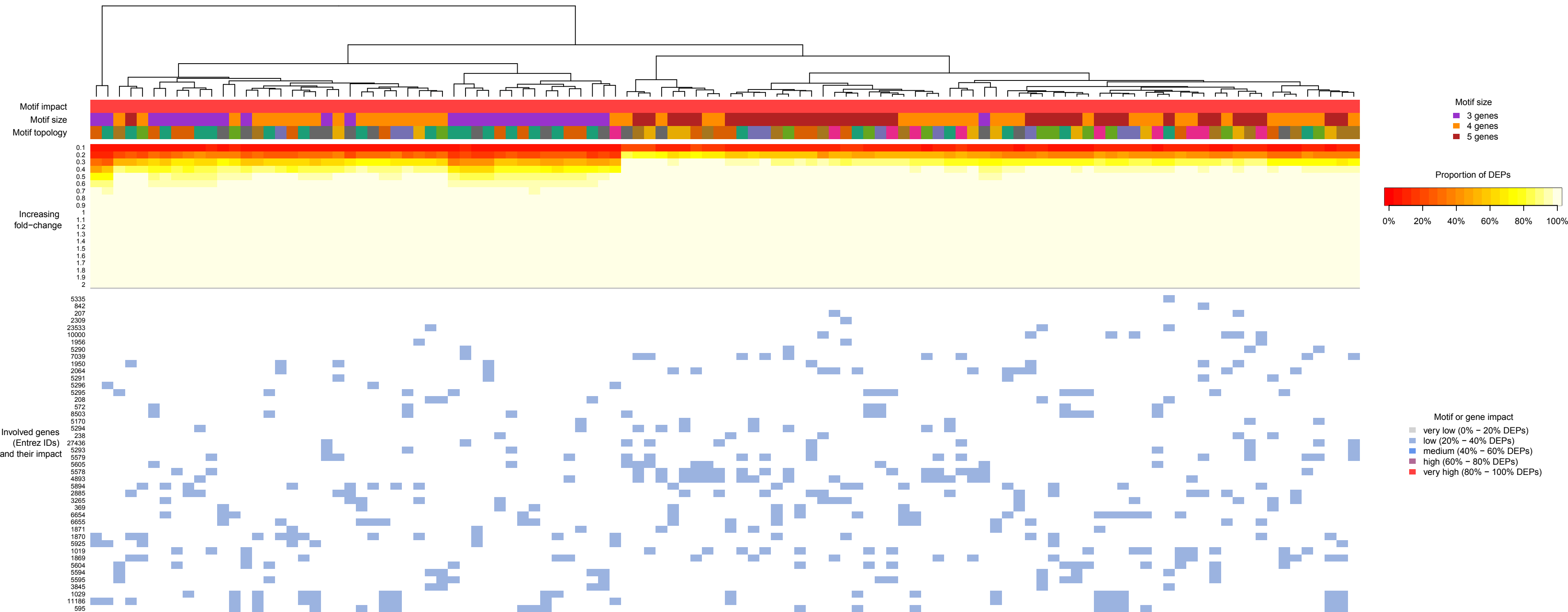

Supplement: S8 Fig — Heatmaps of the proportion of DEPs for all compared methods. (ZIP) [file pone.0191154.s009.zip › S9 Fig/motifs_PRS.pdf]

# Summarization of the Experiment 5 - SPIA

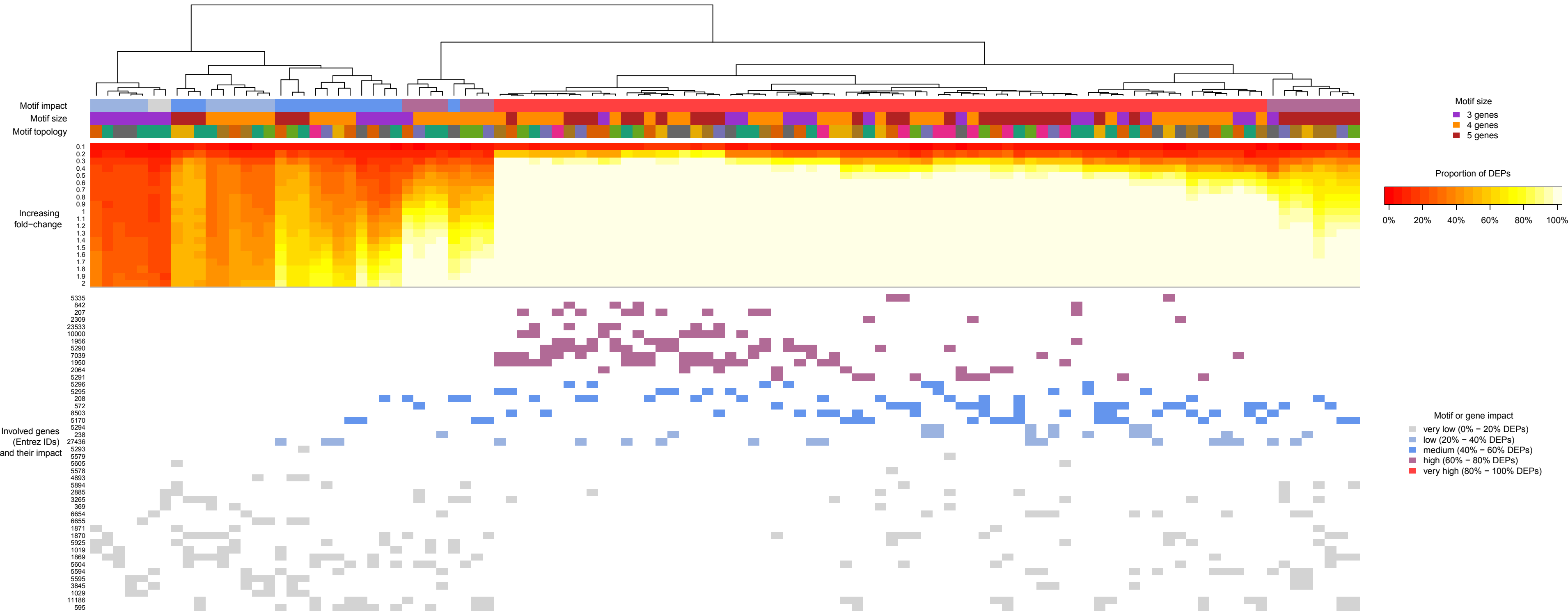

Supplement: S8 Fig — Heatmaps of the proportion of DEPs for all compared methods. (ZIP) [file pone.0191154.s009.zip › S9 Fig/motifs_SPIA.pdf]

# Summarization of the Experiment 5 - TAPPA

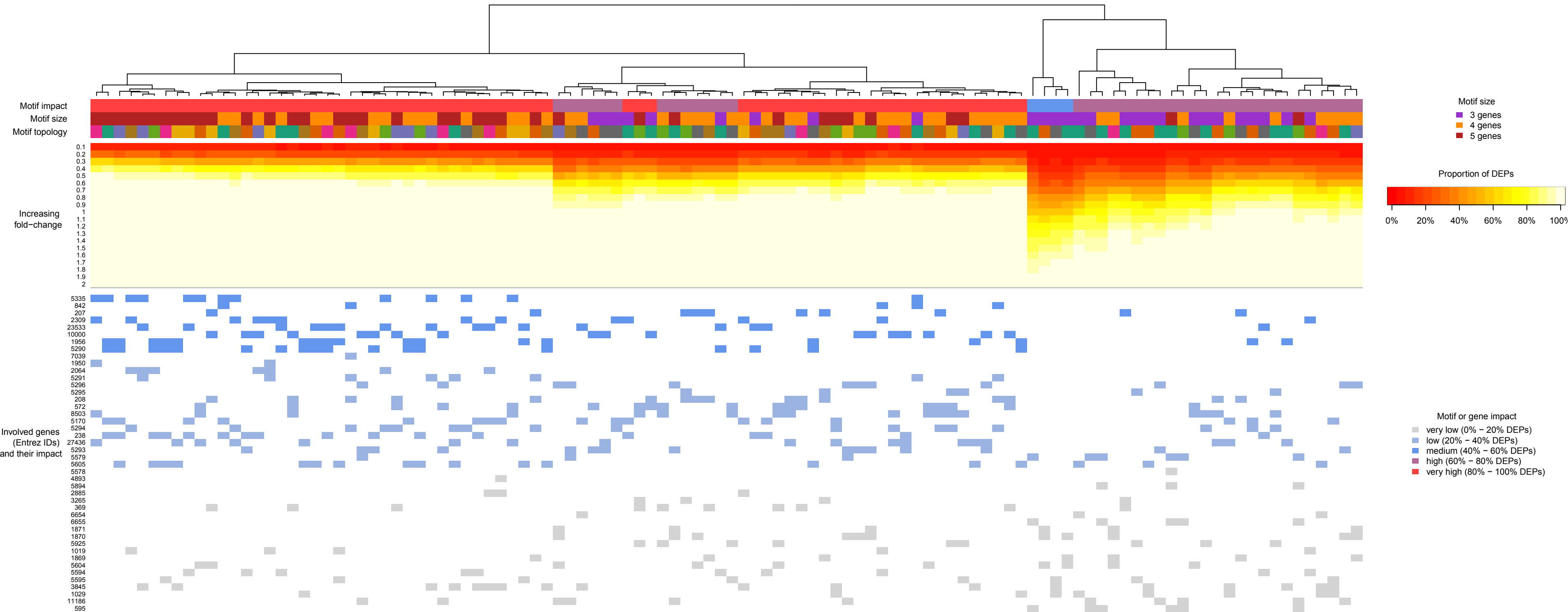

Supplement: S8 Fig — Heatmaps of the proportion of DEPs for all compared methods. (ZIP) [file pone.0191154.s009.zip › S9 Fig/motifs_TAPPA.pdf]

# Summarization of the Experiment 5 - TopologyGSA

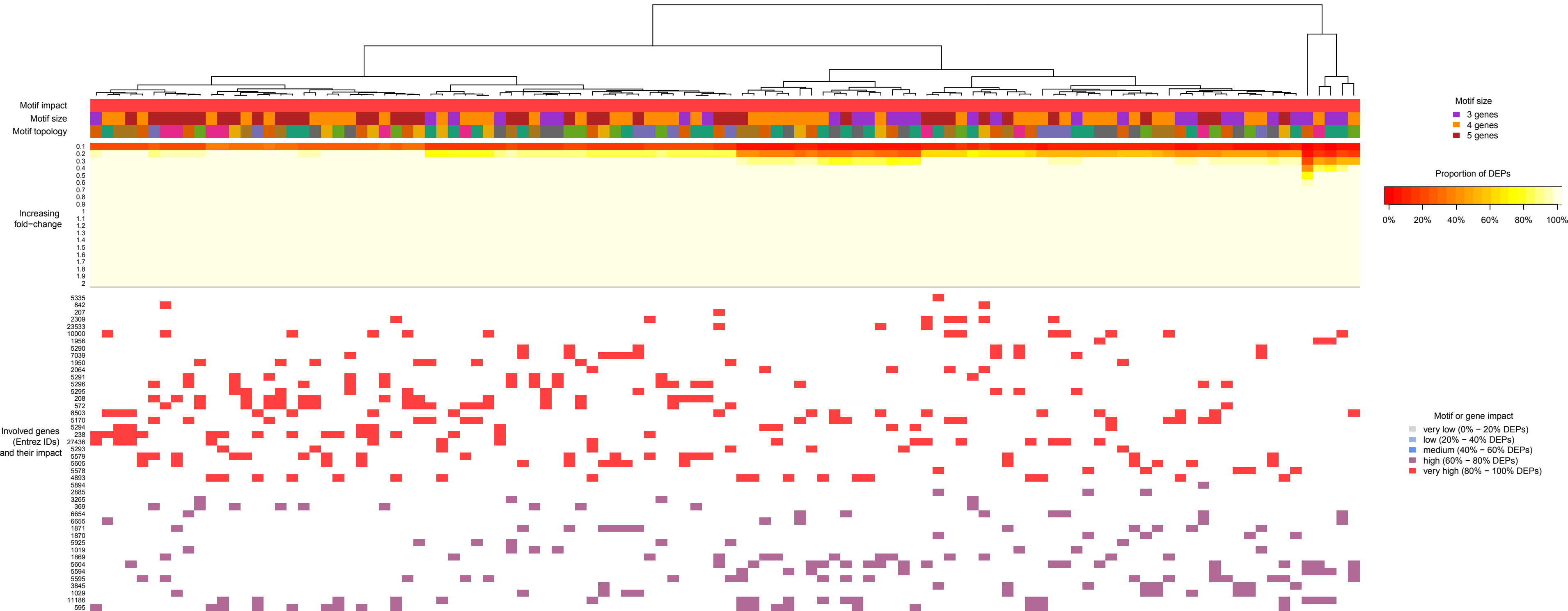

Supplement: S8 Fig — Heatmaps of the proportion of DEPs for all compared methods. (ZIP) [file pone.0191154.s009.zip › S9 Fig/motifs_TopologyGSA.pdf]

# P-values and ranks of the target pathways - Disease-Control Data Collection details

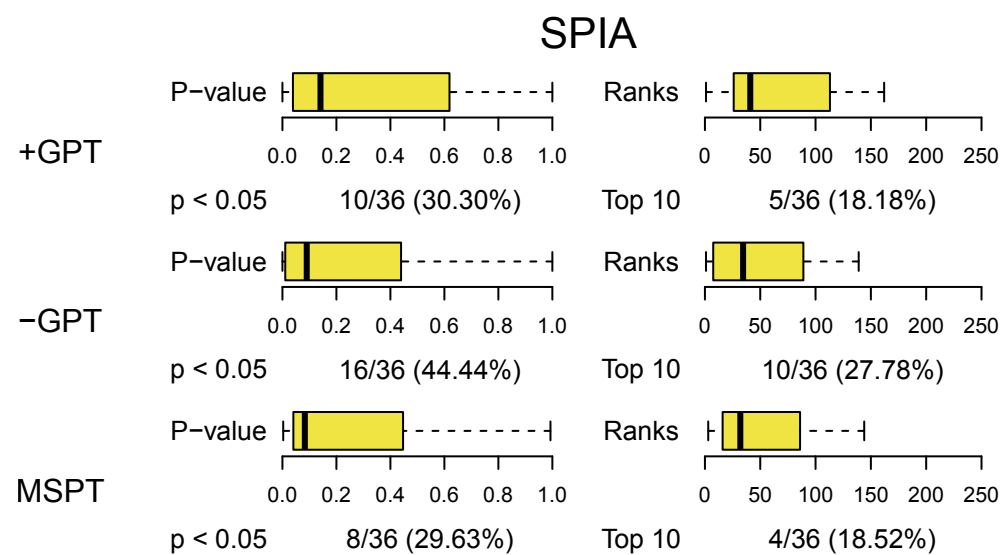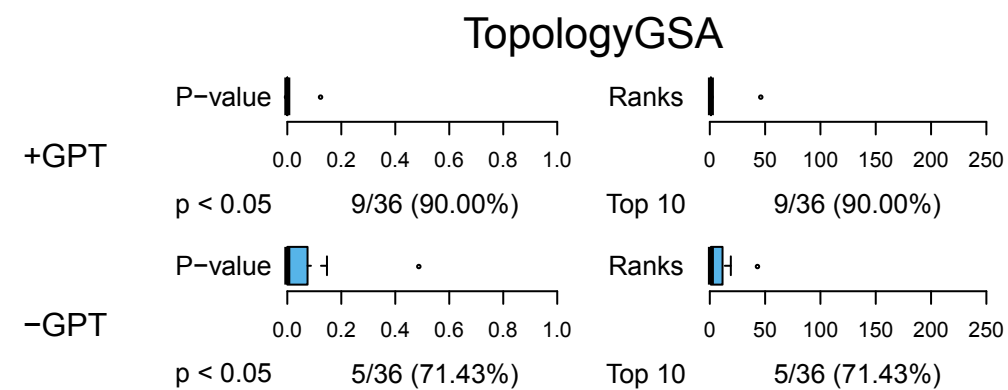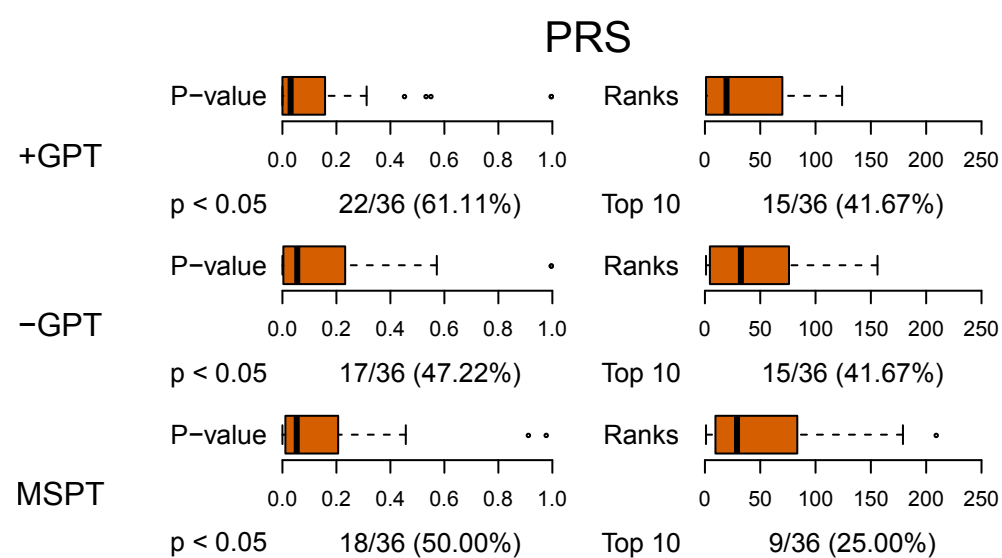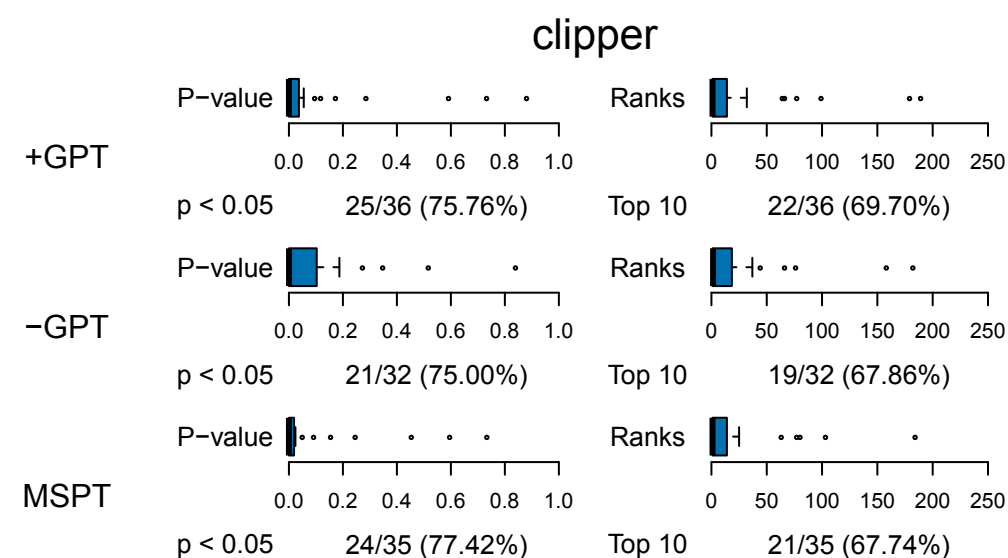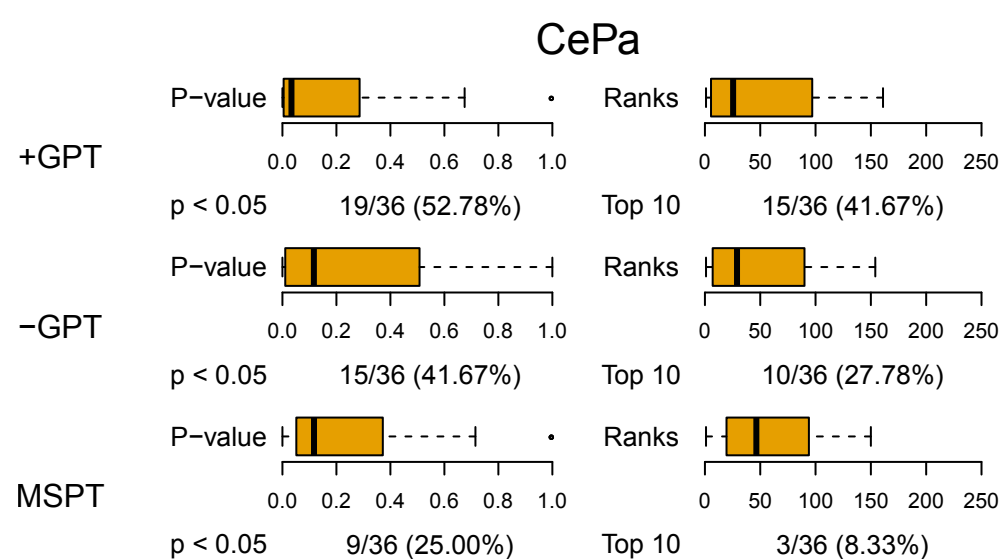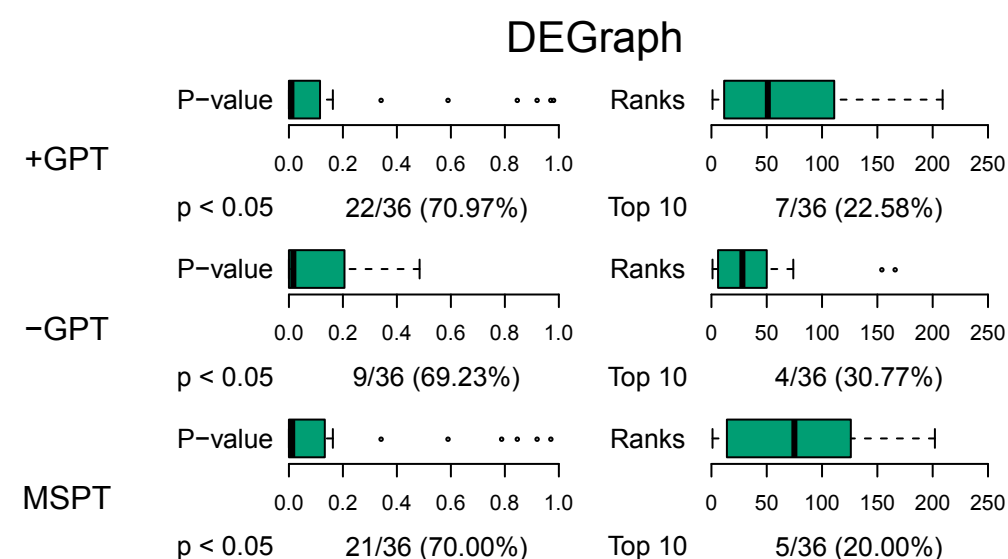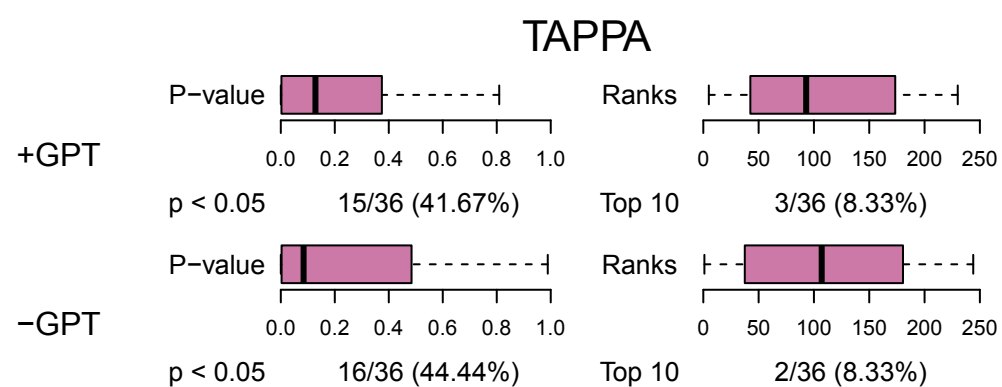

Supplement: S9 Fig — Boxplots of p-values and rank of the estrogen receptor-containing pathways in Disease-Control Data Collection. Ranks are based on p-values. Pathway with the lowest p-value has rank 1. All pathways with the same p-value recieved same rank. The rank was incremented by one between subsequent p-values. (PDF) [file pone.0191154.s010.pdf]

**A**

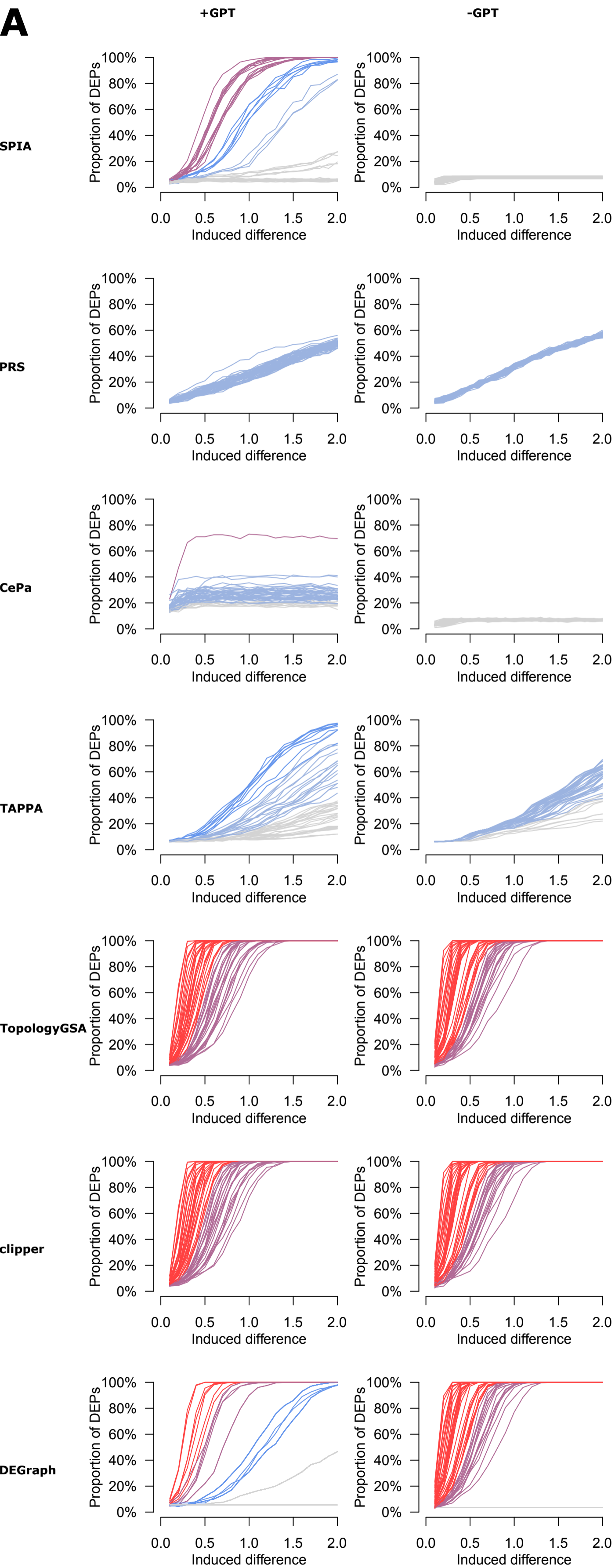

**B**

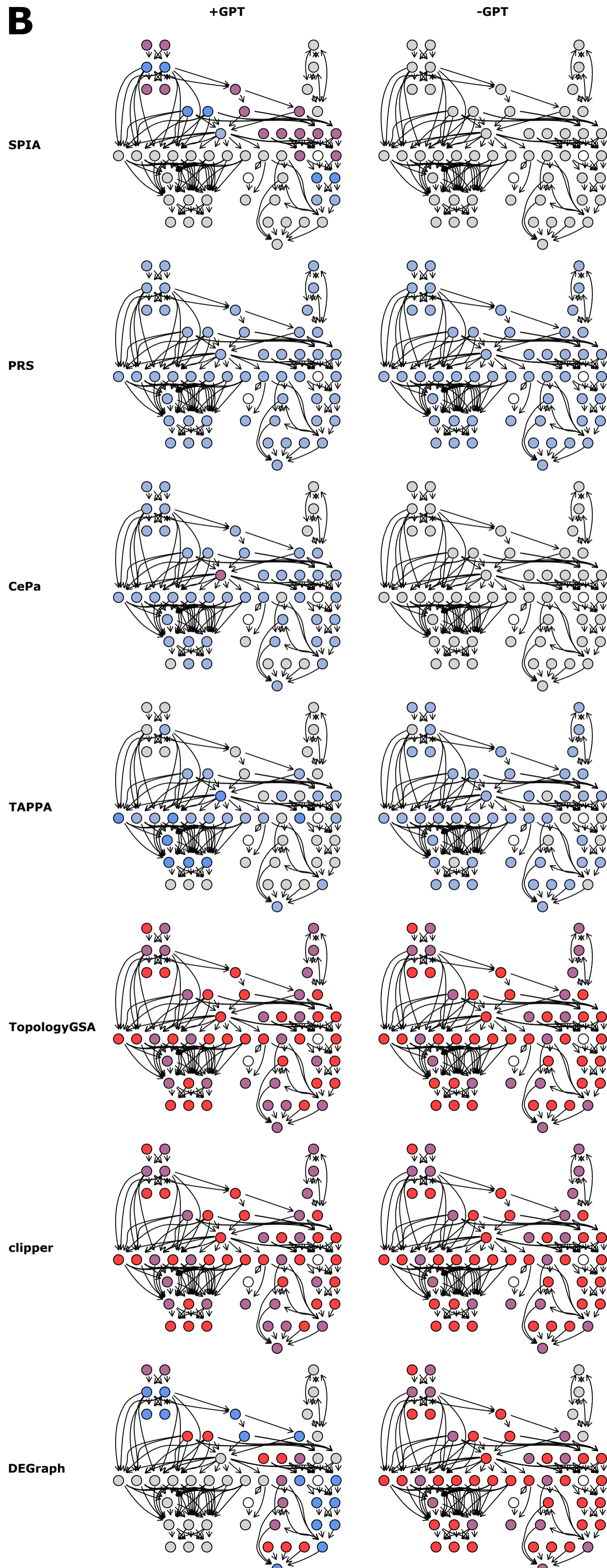

Supplement: S12 Fig — (A) Proportion of differentially expressed pathways for different genes and the difference in expression induced between groups. (B) Dependence of the proportion of differentially expressed pathways on the difference in the gene position. In the non-topological variants of the methods (-GPT) we observed reduced proportion of differentially expressed pathways and loss of its dependence on gene postion in all methods except TopologySGA an Clipper. (PDF) [file pone.0191154.s013.pdf]
