# Supplementary material for: A critical comparison of topology-based pathway analysis methods
Source: PLoS One. 2018 Jan 25;13(1):e0191154. doi: 10.1371/journal.pone.0191154 (PMC5784953; doi:10.1371/journal.pone.0191154)

Summarization of the Experiment 3

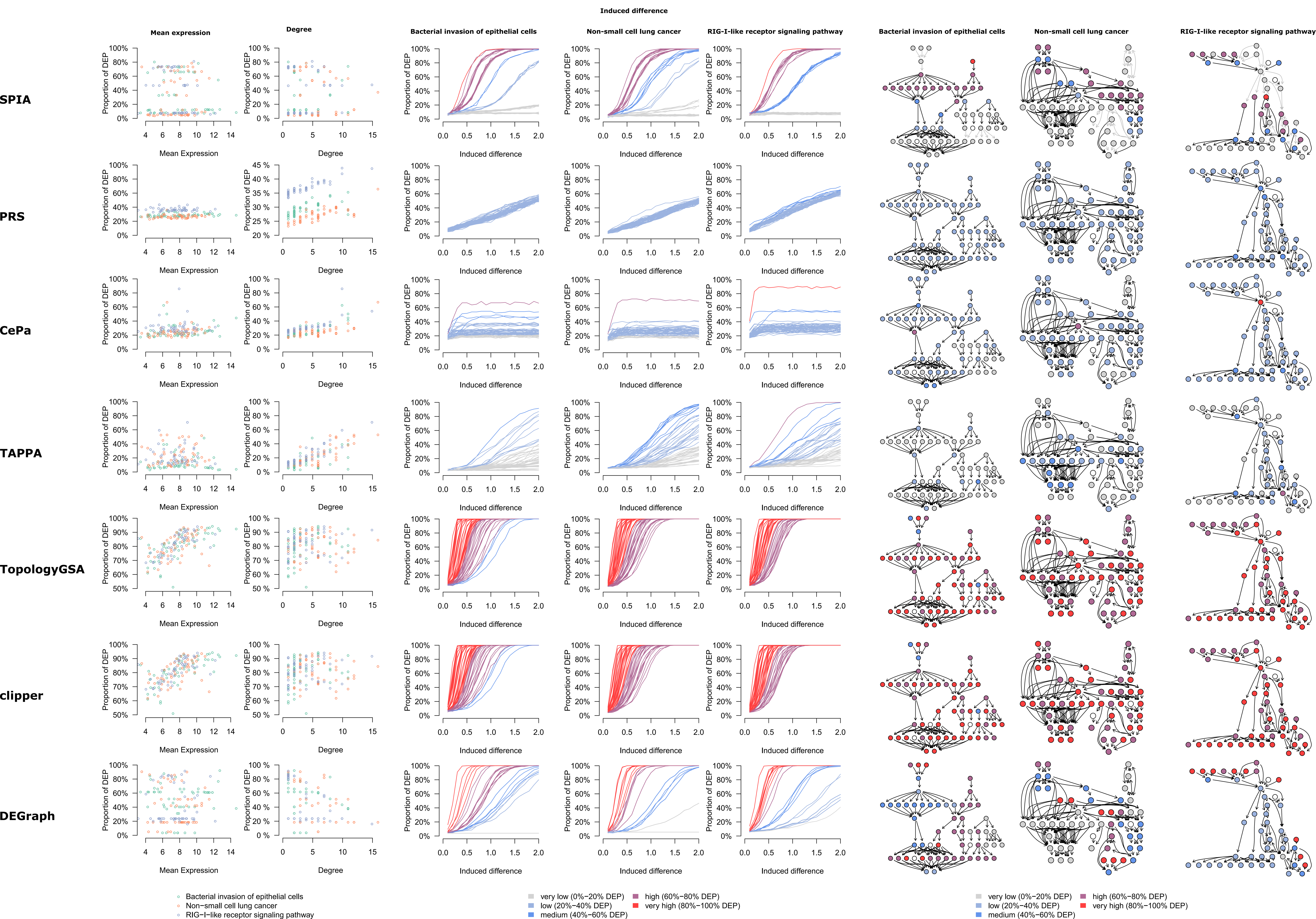

Supplement: S6 Fig — Dependence of the proportion of DEPs on the difference in expression induced between groups, the gene mean expression and its postion. In SPIA, neutral interactions were drawn in grey. (PDF) [file pone.0191154.s007.pdf]
