# Supplementary material for: A critical comparison of topology-based pathway analysis methods
Source: PLoS One. 2018 Jan 25;13(1):e0191154. doi: 10.1371/journal.pone.0191154 (PMC5784953; doi:10.1371/journal.pone.0191154)

# P-values and ranks of the target pathways - Breast Cancer Data Collection details

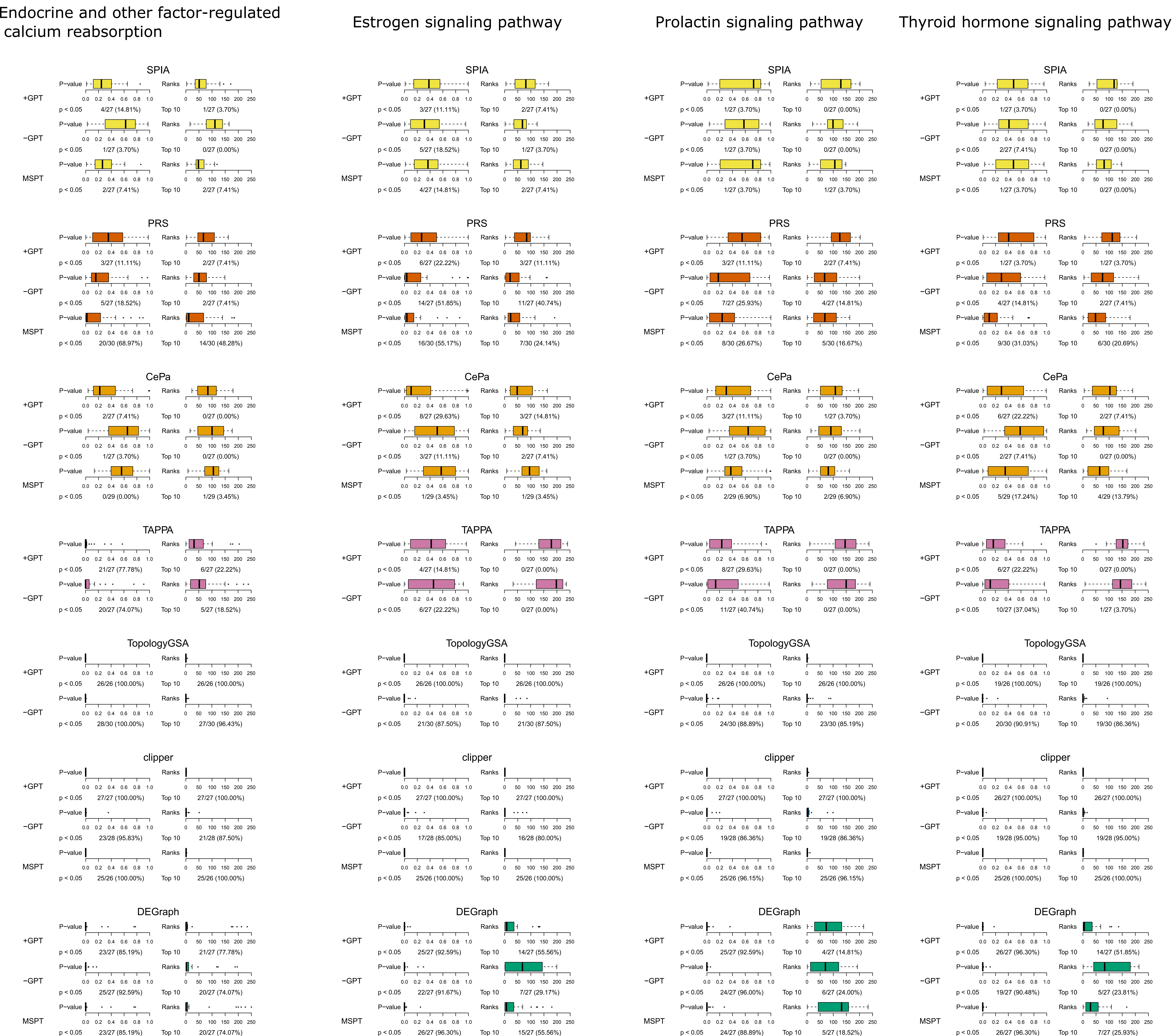

Supplement: S10 Fig — Boxplots of p-values and rank of the estrogen receptor-containing pathways in Breast Cancer Data Collection. Ranks are based on p-values. Pathway with the lowest p-value has rank 1. All pathways with the same p-value recieved same rank. The rank was incremented by one between subsequent p-values. (PDF) [file pone.0191154.s011.pdf]
