# Supplementary material for: A critical comparison of topology-based pathway analysis methods
Source: PLoS One. 2018 Jan 25;13(1):e0191154. doi: 10.1371/journal.pone.0191154 (PMC5784953; doi:10.1371/journal.pone.0191154)
Supplement: S11 Fig — Heatmaps of p-values of the overexpressed oncogene-containing pathways in Gene Overexpression Data Collection. Pathways are ordered by the number of methods in which they are differentially expressed (p < 0.05). (PDF) [file pone.0191154.s012.pdf]

# P-values of the target pathways - Gene Overexpression Data Collection details

c-Myc

DEGraph	SPIA	PRS	CePa	TAPPA	
0.045	0.027	0.005	0.001	0.005	Acute myeloid leukemia
–	0.001	0.005	<0.001	<0.001	Thyroid hormone signaling pathway
0.008	0.023	0.014	0.023	0.070	Thyroid cancer
–	0.001	0.008	<0.001	0.002	Small cell lung cancer
0.007	0.053	0.001	0.002	0.014	Colorectal cancer
0.064	0.017	0.022	0.002	0.011	Chronic myeloid leukemia
0.058	0.040	0.027	0.001	0.013	Central carbon metabolism in cancer
–	0.085	0.129	0.017	0.040	Hepatitis B
0.665	0.011	0.069	0.026	0.532	ErbB signaling pathway
0.079	0.098	0.092	0.009	0.003	Epstein−Barr virus infection
0.406	0.051	0.035	0.060	0.005	Endometrial cancer
–	0.227	0.178	0.022	0.399	Wnt signaling pathway
0.050	0.202	0.118	0.028	0.189	TGF−beta signaling pathway
–	0.253	0.298	0.061	0.004	Signaling pathways regulating pluripotency of stem cells
–	0.398	0.873	0.263	0.332	Cell cycle

H-Ras

DEGraph	SPIA	PRS	CePa	TAPPA	
<0.001	0.026	0.015	0.006	0.976	Thyroid cancer
–	0.005	0.023	<0.001	<0.001	T cell receptor signaling pathway
0.008	0.013	0.008	0.007	0.053	AGE−RAGE signaling pathway in diabetic complications
<0.001	0.012	0.107	0.018	0.699	VEGF signaling pathway
–	0.021	0.007	0.037	0.761	Signaling pathways regulating pluripotency of stem cells
0.011	0.068	0.024	0.015	0.973	Prolactin signaling pathway
–	0.006	0.022	0.004	0.815	Hepatitis B
0.117	0.005	0.267	0.036	0.005	Estrogen signaling pathway
<0.001	0.032	0.073	0.034	0.539	Bladder cancer
0.006	0.012	0.118	0.016	0.449	B cell receptor signaling pathway
–	<0.001	0.090	0.022	0.007	Apoptosis
0.001	0.253	0.294	0.027	0.440	Serotonergic synapse
–	0.019	0.129	0.017	0.835	Neurotrophin signaling pathway
<0.001	0.276	0.745	0.347	0.018	Longevity regulating pathway − multiple species
0.032	0.190	0.680	0.054	0.034	Longevity regulating pathway
0.044	0.018	0.188	0.112	0.853	GnRH signaling pathway
0.025	0.112	0.080	0.039	0.209	Acute myeloid leukemia
–	0.802	0.291	0.006	0.089	Tight junction
–	0.186	0.326	0.235	0.003	Thyroid hormone signaling pathway
0.108	0.133	0.271	0.015	0.065	Prostate cancer
<0.001	0.104	0.651	0.395	0.218	Non−small cell lung cancer
–	0.239	0.310	0.194	0.044	Natural killer cell mediated cytotoxicity
–	0.505	0.216	0.162	0.008	Melanoma
–	0.046	0.164	0.140	0.794	Melanogenesis
0.002	0.607	0.552	0.184	0.309	Long−term potentiation
0.030	0.602	0.561	0.426	0.218	Long−term depression
–	0.165	0.204	0.069	0.031	Insulin signaling pathway
<0.001	0.914	0.476	0.283	0.584	Glioma
0.006	0.871	0.818	0.240	0.215	Fc epsilon RI signaling pathway
0.102	<0.001	0.578	0.191	0.357	ErbB signaling pathway
0.001	0.186	0.149	0.080	0.090	Chronic myeloid leukemia
<0.001	0.267	0.215	0.113	0.114	Choline metabolism in cancer
–	0.397	0.153	0.081	0.096	Sphingolipid signaling pathway
–	0.147	0.326	0.246	0.674	Phospholipase D signaling pathway
–	0.316	0.114	0.090	0.059	Hepatitis C
–	0.365	0.622	0.212	0.944	Gap junction
–	0.166	0.346	0.101	0.889	FoxO signaling pathway
0.132	0.342	0.172	0.165	0.763	Endometrial cancer
–	0.111	0.263	0.072	0.352	Cholinergic synapse
0.080	0.596	0.766	0.386	0.681	Central carbon metabolism in cancer

c-Src

DEGraph	SPIA	PRS	CePa	TAPPA	
0.124	0.003	0.002	0.002	0.039	Epithelial cell signaling in Helicobacter pylori infection
–	0.039	0.252	0.383	0.007	Estrogen signaling pathway
0.015	0.181	0.144	0.103	0.050	Endocytosis
0.143	0.530	0.009	0.011	0.602	Bacterial invasion of epithelial cells
–	NaN	0.004	0.002	0.719	Adherens junction
0.022	0.073	0.655	0.234	0.466	VEGF signaling pathway
0.042	0.938	0.440	0.161	0.380	Prolactin signaling pathway
–	<0.001	0.459	0.133	0.956	Platelet activation
–	<0.001	0.346	0.290	0.543	GnRH signaling pathway
–	0.023	0.206	0.186	0.202	Gap junction
–	0.057	0.625	0.489	0.807	Thyroid hormone signaling pathway
0.126	0.164	0.450	0.407	0.931	Inflammatory mediator regulation of TRP channels
–	0.363	0.109	0.060	0.334	Hepatitis B
–	0.434	0.229	0.193	0.056	ErbB signaling pathway
